# Immunosuppression therapy for idiopathic membranous nephropathy: systematic review with network meta-analysis

**DOI:** 10.1007/s40620-022-01268-2

**Published:** 2022-02-23

**Authors:** Bhadran Bose, Edmund Y. M. Chung, Regina Hong, Giovanni F. M. Strippoli, David W. Johnson, Wen-ling Yang, Sunil V. Badve, Suetonia C. Palmer

**Affiliations:** 1 Australasian Kidney Trials Network, The University of Queensland, Queensland, Australia; 2 Department of Nephrology, Nepean Hospital, Kingswood, NSW 2747 Australia; 3 Centre for Kidney Research, Cochrane Kidney and Transplant, The Children’s Hospital at Westmead, Westmead, NSW Australia; 4 Department of Nephrology, St George Hospital, Sydney, Australia; 5 Department of Emergency and Organ Transplantation, University of Bari, Bari, Italy; 6 Sydney School of Public Health, The University of Sydney, Sydney, Australia; 7 Department of Nephrology, Princess Alexandra Hospital, Brisbane, Australia; 8 Translational Research Institute, Brisbane, Australia; 9 Department of Nephrology, Peking University Third Hospital, Beijing, China; 10 UNSW Medicine, The George Institute for Global Health, Sydney, Australia; 11 Department of Medicine, University of Otago, Christchurch, Christchurch, New Zealand

**Keywords:** Membranous nephropathy, Network meta-analysis, Cytotoxic agents, Calcineurin inhibitor, Rituximab

## Abstract

**Background:**

Idiopathic membranous nephropathy is a common cause of nephrotic syndrome in adults. The Kidney Disease Improving Global Outcomes guidelines recommend rituximab or cyclophosphamide and steroids, or calcineurin inhibitor-based therapy. However, there have been few or no head-to-head comparisons of the relative efficacy and safety of different immunosuppression regimens. We conducted a network meta-analysis to evaluate the comparative efficacy and safety of available immunosuppression strategies compared to cyclophosphamide in adults with idiopathic membranous nephropathy.

**Methods:**

We performed a systematic search of MEDLINE, Embase and CENTRAL for randomized controlled trials in the treatment of adults with idiopathic membranous nephropathy. The primary outcome was complete remission. Secondary outcomes were kidney failure, partial remission, estimated glomerular filtration rate, doubling of serum creatinine, proteinuria, serious adverse events, discontinuation of treatment, serious infection and bone marrow suppression.

**Results:**

Cyclophosphamide had uncertain effects on inducing complete remission when compared to rituximab (OR 0.35, CI 0.10–1.24, low certainty evidence), mycophenolate mofetil (OR 1.81, CI 0.69–4.71, low certainty), calcineurin inhibitor (OR 1.26, CI 0.61–2.63, low certainty) or steroid monotherapy (OR 2.31, CI 0.62–8.52, low certainty). Cyclophosphamide had a higher probability of inducing complete remission when compared to calcineurin inhibitor plus rituximab (OR 4.45, CI 1.04–19.10, low certainty). Compared to other immunosuppression strategies, there was limited evidence that cyclophosphamide had different effects on other pre-specified outcomes.

**Conclusions:**

The comparative effectiveness and safety of immunosuppression strategies compared to cyclophosphamide is uncertain in adults with idiopathic membranous nephropathy.

**Graphical abstract:**

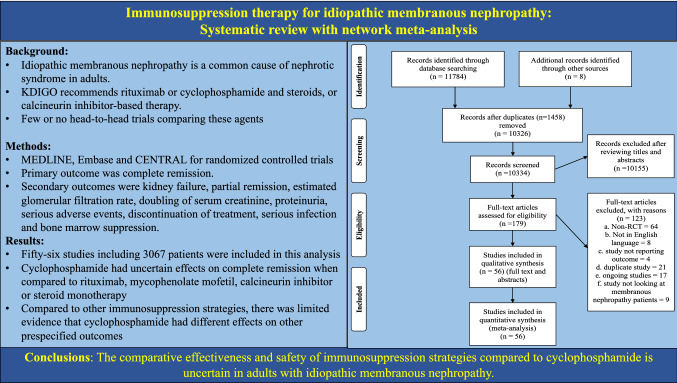

**Supplementary Information:**

The online version contains supplementary material available at 10.1007/s40620-022-01268-2.

## Introduction

Membranous nephropathy is a leading cause of idiopathic nephrotic syndrome in adults [1]. Approximately 35–47% of patients with persistent nephrotic syndrome progress to kidney failure within 10 years [2–4]. The 2021 update of the Kidney Disease Improving Global Outcomes (KDIGO) guidelines on glomerular disease makes a level 1B recommendation that patients with membranous nephropathy and at least one risk factor for disease progression (such as life-threatening nephrotic syndrome or rapid deterioration of kidney function) should be treated with “rituximab or cyclophosphamide and alternate month steroids for 6 months, or calcineurin inhibitor-based therapy for ≥ 6 months, with the choice of treatment depending on the risk estimate” [5]. For those at very high risk, cyclophosphamide and steroids are recommended. However, the optimal form of immunosuppression in membranous nephropathy has not been definitively established. Alkylating agents like cyclophosphamide frequently incur harm, including myelosuppression, infertility, bladder cancer, leukaemia, skin cancer and haemorrhagic cystitis [6–9]. Calcineurin inhibitors are effective in inducing remission, but there is nearly a 50% risk of relapse once they are stopped [10]. Rituximab alone or in combination with a calcineurin inhibitor has been shown to induce disease remission and incur fewer serious adverse events [11–14]. However, there have been few or no head-to-head trials of specific agents comparing their relative effectiveness and safety profiles in people with membranous nephropathy. Accordingly, a network meta-analysis of randomized trials may enable the comparative analysis of immunosuppression strategies against each other in the absence of head-to-head trials and may help better inform shared treatment decisions between clinicians and patients with membranous nephropathy.

We conducted a systematic review with network meta-analysis of randomized controlled trials comparing the benefits and harms of immunosuppression strategies compared to cyclophosphamide in adults with idiopathic membranous nephropathy.

## Materials and methods

We conducted this systematic review in accordance with the PRISMA (Preferred Reporting Items for Systematic Reviews and Meta-Analyses) extension statement for network analyses [15]. The study protocol was registered in PROSPERO (CRD 42018116241) prior to data extraction.

### Data sources and searches

Electronic searches of MEDLINE (from 1946 to the 23rd of July 2021), Embase (from 1974 to the 23rd of July 2021) and the CENTRAL (Cochrane Central Register of Controlled Trials) (issue 7 of 2021) were conducted without language restriction using the search strategy described in Supplement Table 1.

### Study selection

Parallel-group randomized controlled trials evaluating immunosuppression strategies in the treatment of adults with biopsy-proven idiopathic membranous nephropathy were eligible. Studies were eligible if there was follow-up of clinical outcomes for 6 months or longer.

Intervention strategies included cyclophosphamide, chlorambucil, rituximab, calcineurin inhibitors (cyclosporine or tacrolimus), calcineurin inhibitor plus rituximab, mycophenolate mofetil, azathioprine, ACTH, steroids alone, placebo or non-immunosuppressive therapy. Non-immunosuppressive therapy included treatment with antiproteinuric agents such as angiotensin-converting enzyme inhibitors or angiotensin receptor blockers.

Two reviewers (BB and RH) independently evaluated the title and abstract of all retrieved search records to determine potential eligibility. The same two reviewers reviewed any potentially eligible citations in full text and supplementary data. Any difference in assessments between reviewers were resolved by consensus and arbitration by a third author (SVB) if necessary.

### Data extraction and quality assessment

Two reviewers (BB and RH) independently extracted data into a pre-specified purpose-built database and adjudicated study risk of bias using the Cochrane risk of bias assessment tool [16]. Any disagreements were resolved via consultation with a third author (SVB). Extracted data included study design, population and intervention characteristics, risk of bias and outcome data. Corresponding authors were contacted by electronic mail to request missing data. We excluded studies that were not in the English language.

### Outcomes

The primary review outcome was complete remission. Secondary outcomes were partial remission, kidney failure (defined as commencement of dialysis), estimated glomerular filtration rate, doubling of serum creatinine, proteinuria, serious adverse events, discontinuation of treatment, serious infection, onset of diabetes mellitus and bone marrow suppression. Outcomes were used as defined by trial investigators.

### Data synthesis and analysis

To evaluate the assumption of transitivity (that the included studies were sufficiently similar with regard to design and trial population to form an analytical network), the clinical setting and methodological characteristics of included trials were evaluated to assess whether they were sufficiently similar and that a network meta-analysis was appropriate [17].

Treatment effects were estimated using random-effects pairwise meta-analysis [18]. Estimated treatment effects were summarized as an odds ratio (OR) with 95% confidence intervals (CI) for binary outcomes (complete remission, kidney failure, partial remission, serious adverse event, discontinuation of treatment, serious infection, diabetes mellitus, bone marrow suppression) or mean difference (MD) or standardized mean difference (SMD) for continuous outcomes (glomerular filtration rate and proteinuria). Statistical heterogeneity between studies was estimated using Chi square and the I^2^ test. I^2^ values over 25%, 50%, and 75% were considered to correspond to low, moderate and high levels of heterogeneity, respectively [19].

Treatment estimates were then calculated using random effects network meta-analysis using frequentist methods with treatment effects expressed relative to cyclophosphamide. The extent of heterogeneity in each formed network was evaluated by using the restricted maximum likelihood method to generate a common heterogeneity variance (tau [τ]) with an empirical distribution of heterogeneity variances, considering the range of expected treatment estimates (ORs and SMDs). Values of τ from 0.1 to 0.5 were low, 0.5–1.0 were considered fairly high, and greater than 1.0 represented fairly extreme heterogeneity [20].

To explore network inconsistency between direct and indirect evidence, a node-splitting approach was used. A global “design-by-treatment” approach was used to check the assumption of consistency [21]. Subgroup analysis was planned a priori on complete remission at 24 months, partial remission at 24 months, baseline degree of proteinuria (less than 4, 4–8 and more than 8 g/day), phospholipase A2 receptor antibody (positive or negative), kidney function (glomerular filtration rate less than or more than 60 mL/min/1.73 m^2^), age (less than or more than 60 years), sex, ethnicity (White, Black, Asian, Hispanic and others) and study duration (less than or more than 12 months). Small study effects (publication bias) in meta-analytical estimates of treatment effects on the primary outcomes were assessed using a comparison-adjusted funnel plot when there were enough data for observations (10 or more trials). Analysis was conducted in Stata, version 15 (StataCorp LP) using published Stata routines [22].

Certainty in the evidence was assessed using the Grading of Recommendations Assessment, Development and Evaluation (GRADE) process for network meta-analysis taking into consideration outcome-specific study limitations, indirectness of evidence, imprecision, transitivity, publication bias and consistency of direct and indirect treatment estimates [23].

## Results

### Selection and description of studies

**Fig. 1:**
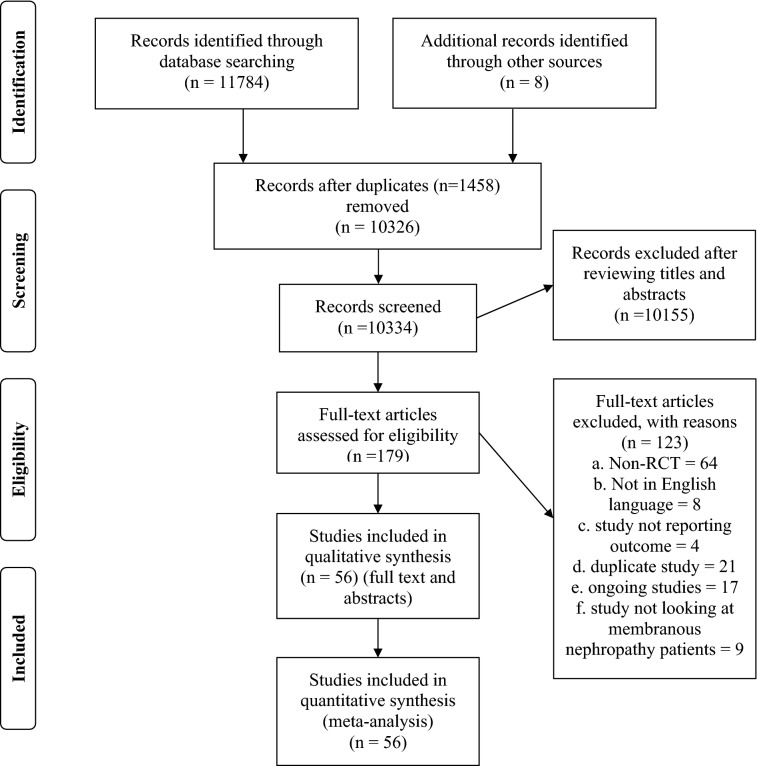
PRISMA flowchart

Fifty-six studies including 3067 patients proved eligible [10–14, 24–75] (Fig. 1; Supplementary Table 2). The studies had a median sample size of 53 participants (range 9–190). The mean study age ranged between 32 and 75.1 years (median 47.4 years). The mean study baseline creatinine ranged from 0.74 to 2.7 mg/dL (median 1.18 mg/dL) and the mean study 24-h urine protein excretion ranged between 1.64 and 12.8 g/day (median 7.86 g/day). Follow-up for clinical outcomes ranged from 6 months to 10 years (median 23.4 months). Of the 56 studies included in the analysis, 51 had 2 arms and 5 had three arms in the study design.

Cyclophosphamide was compared with steroid monotherapy (3 trials, 137 participants) [27, 41, 57], calcineurin inhibitor (4 trials, 299 participants) [34, 44, 64, 72], rituximab (1 trial, 74 participants) [14], calcineurin inhibitor plus rituximab (1 trial, 86 participants) [12], chlorambucil (3 trials, 145 participants) [29, 59, 65], mycophenolate mofetil (1 trial, 22 participants) [68], mizoribine (1 trial, 55 participants) [71], ACTH (1 trial, 32 participants) [60], non-immunosuppressive therapy (4 trials, 209 participants) [38, 47, 54, 70] and Chinese herbal medicine (1 trial, 190 participants) [35]. There were 3 trials with 59 participants, which compared different protocols of cyclophosphamide (early versus late initiation or intravenous versus oral) [37, 45]. Chlorambucil was compared with steroid monotherapy (1 trial, 20 participants) [26], mycophenolate mofetil (1 trial, 20 participants) [33] and non-immunosuppressive therapy (2 trials, 130 participants) [61, 62]. Calcineurin inhibitors were compared with rituximab (1 trial, 130 participant) [13], non-immunosuppressive therapy (3 trials, 95 participants) [32, 63, 74], steroid monotherapy (1 trial, 51 participants) [10] and azathioprine (1 trial, 23 participants) [55]. There were 7 trials with 290 participants, which compared calcineurin inhibitors against calcineurin inhibitor (such as different doses or cyclosporin versus tacrolimus) [48, 51, 52, 56, 67, 69, 75]. Steroid monotherapy was compared with placebo (2 trials, 203 participants) [31, 36] and non-immunosuppressive therapy (1 trial, 40 participants) [30]. Mycophenolate mofetil was also compared with non-immunosuppressive therapy (2 trials, 77 participants) [39, 40]. Mycophenolate mofetil was compared with a combination of calcineurin inhibitor and mycophenolate mofetil (1 trial, 20 participants) [73]. Rituximab was compared with non-immunosuppressive therapy (1 trial, 75 participants) [11]. Mizoribine was compared with steroid monotherapy (1 trial, 36 participants) [43] and also against different doses of mizoribine (1 trial, 37 participants) [66]. Azathioprine was compared with non-immunosuppressive therapy (2 trials, 23 participants) [24, 25]. Pentoxifylline was compared with placebo (1 trial, 18 participants) [28].

Of the 5 studies with 3 arms in the study design, one compared chlorambucil with steroid monotherapy and non-immunosuppressive therapy (60 participants) [42], one compared cyclophosphamide with calcineurin inhibitor and non-immunosuppressive therapy (28 participants) [50], one compared chlorambucil with calcineurin inhibitor and non-immunosuppressive therapy (108 participants) [46], one compared cyclophosphamide with mycophenolate mofetil and calcineurin inhibitor (90 participants) [58] and one compared cyclophosphamide with leflunomide and a combination of cyclophosphamide plus leflunomide (72 participants) [53].

In the cyclophosphamide group, all studies except one [38] used steroids along with cyclophosphamide. The definition of complete and partial remission was not uniform across all the studies (Supplementary Table 3).

### Risk of bias

Seventeen (29.3%) studies were adjudicated as being at low risk of bias in methods used to generate the random sequence and 12 (20.6%) studies were at low risk of bias in methods of allocation concealment (Supplementary Table 4). Only two (3.7%) studies reported blinding of participants and investigators. None of the studies reported blinding of outcome assessment. Twelve (20.6%) studies were adjudicated as being at low risk of incomplete outcomes and selective reporting.

### Effects of interventions

**Fig. 2:**
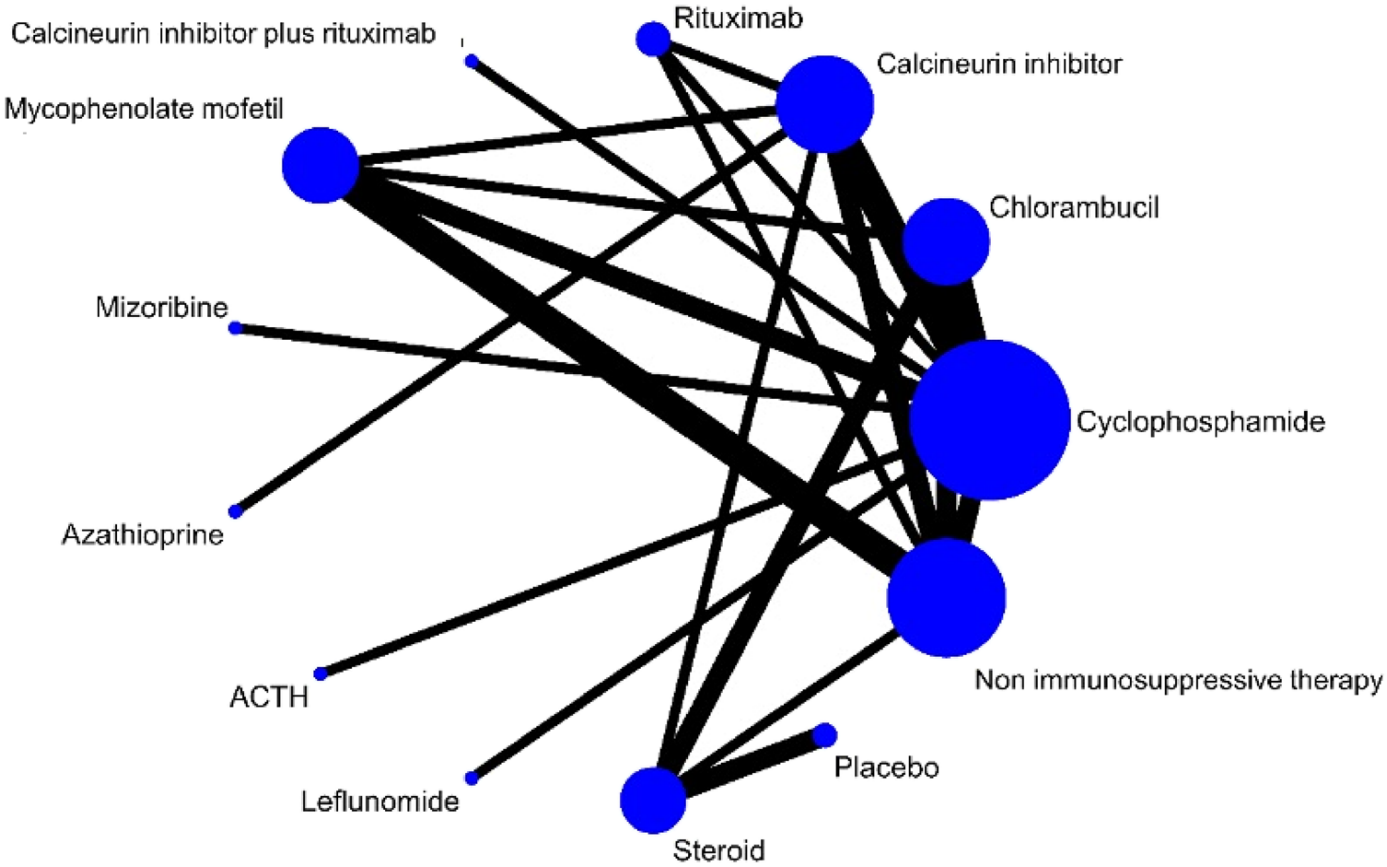
Network plots for effects of immunosuppression on disease complete and partial remission in idiopathic membranous nephropathy. The size of each node is proportional to the sample size and the width of the lines represents the number of each pairwise comparison. *ACTH* adrenocorticotropic hormone

Figure 2 and Supplementary Fig. 1 (1.1–1.9) show the formed evidence network for each outcome. There was no strong evidence for global network inconsistency (Supplementary Table 7). Direct and indirect estimates were generally coherent (Supplementary Table 5). Tables 1, 2 and Supplementary Table 8 present the network estimates for each strategy comparison for all outcomes.

**Table 1:** Network estimates (odds ratios and 95% CI) of effects of treatment on complete remission

Cyclophosphamide	0.89 (0.36, 2.16)	0.79 (0.38, 1.65)	2.87 (0.81, 10.22)	0.22 (0.05, 0.96)	0.55 (0.21, 1.44)	0.51 (0.10, 2.52)	0.53 (0.06, 4.72)	3.00 (0.46, 19.59)	0.24 (0.04, 1.52)	0.43 (0.12, 1.60)	0.21 (0.03, 1.39)	0.32 (0.15, 0.69)
1.13 (0.46, 2.75)	Chlorambucil	0.89 (0.32, 2.52)	3.24 (0.72, 14.55)	0.25 (0.05, 1.40)	0.62 (0.20, 1.90)	0.57 (0.09, 3.58)	0.60 (0.06, 6.00)	3.38 (0.42, 27.01)	0.27 (0.03, 2.10)	0.49 (0.15, 1.64)	0.23 (0.04, 1.47)	0.36 (0.14, 0.91)
1.26 (0.61, 2.63)	1.12 (0.40, 3.16)	Calcineurin inhibitor	3.63 (0.92, 14.40)	0.28 (0.06, 1.45)	0.70 (0.25, 1.95)	0.64 (0.11, 3.74)	0.67 (0.08, 5.26)	3.79 (0.51, 28.42)	0.30 (0.04, 2.20)	0.55 (0.14, 2.09)	0.26 (0.04, 1.79)	0.40 (0.17, 0.93)
0.35 (0.10, 1.24)	0.31 (0.07, 1.39)	0.28 (0.07, 1.09)	Rituximab	0.08 (0.01, 0.54)	0.19 (0.04, 0.88)	0.18 (0.02, 1.36)	0.18 (0.02, 2.20)	1.04 (0.11, 10.05)	0.08 (0.01, 0.78)	0.15 (0.03, 0.88)	0.07 (0.01, 0.68)	0.11 (0.03, 0.43)
4.45 (1.04, 19.10)	3.95 (0.71, 21.77)	3.52 (0.69, 17.99)	12.79 (1.85, 88.27)	Calcineurin inhibitor plus rituximab	2.46 (0.43, 14.06)	2.26 (0.26, 19.71)	2.35 (0.17, 32.63)	13.35 (1.24, 143.57)	1.06 (0.10, 11.17)	1.93 (0.27, 13.66)	0.92 (0.08, 10.10)	1.41 (0.27, 7.35)
1.81 (0.69, 4.71)	1.60 (0.53, 4.88)	1.43 (0.51, 4.01)	5.20 (1.14, 23.73)	0.41 (0.07, 2.32)	Mycophenolate mofetil	0.92 (0.14, 5.94)	0.95 (0.09, 9.59)	5.43 (0.66, 44.62)	0.43 (0.05, 3.46)	0.78 (0.18, 3.38)	0.37 (0.05, 2.79)	0.58 (0.22, 1.50)
1.97 (0.40, 9.75)	1.74 (0.28, 10.90)	1.56 (0.27, 9.05)	5.65 (0.73, 43.59)	0.44 (0.05, 3.85)	1.09 (0.17, 7.02)	Mizoribine	1.04 (0.07, 15.66)	5.90 (0.50, 69.50)	0.47 (0.04, 5.41)	0.85 (0.11, 6.73)	0.41 (0.03, 4.89)	0.63 (0.11, 3.69)
1.90 (0.21, 16.97)	1.68 (0.17, 16.96)	1.50 (0.19, 11.84)	5.45 (0.45, 65.23)	0.43 (0.03, 5.92)	1.05 (0.10, 10.53)	0.96 (0.06, 14.55)	Azathioprine	5.69 (0.32, 101.83)	0.45 (0.03, 7.95)	0.82 (0.07, 9.64)	0.39 (0.02, 6.60)	0.60 (0.06, 5.61)
0.33 (0.05, 2.18)	0.30 (0.04, 2.36)	0.26 (0.04, 1.98)	0.96 (0.10, 9.23)	0.07 (0.01, 0.81)	0.18 (0.02, 1.51)	0.17 (0.01, 2.00)	0.18 (0.01, 3.15)	ACTH	0.08 (0.01, 1.11)	0.14 (0.01, 1.42)	0.07 (0.00, 1.00)	0.11 (0.01, 0.81)
4.20 (0.66, 26.74)	3.72 (0.48, 29.06)	3.32 (0.45, 24.34)	12.07 (1.28, 113.85)	0.94 (0.09, 9.95)	2.32 (0.29, 18.66)	2.14 (0.18, 24.68)	2.22 (0.13, 39.04)	12.60 (0.90, 175.78)	Leflunomide	1.82 (0.19, 17.55)	0.87 (0.06, 12.34)	1.34 (0.18, 9.91)
2.31 (0.62, 8.52)	2.05 (0.61, 6.88)	1.83 (0.48, 6.97)	6.63 (1.14, 38.58)	0.52 (0.07, 3.67)	1.27 (0.30, 5.49)	1.17 (0.15, 9.27)	1.22 (0.10, 14.28)	6.92 (0.70, 68.11)	0.55 (0.06, 5.29)	Steroid	0.48 (0.12, 1.90)	0.73 (0.21, 2.59)
4.83 (0.72, 32.37)	4.29 (0.68, 26.93)	3.83 (0.56, 26.21)	13.89 (1.48, 130.29)	1.09 (0.10, 11.92)	2.67 (0.36, 19.93)	2.46 (0.20, 29.52)	2.55 (0.15, 42.93)	14.50 (1.00, 209.69)	1.15 (0.08, 16.35)	2.10 (0.53, 8.34)	Placebo	1.54 (0.24, 9.98)
3.14 (1.46, 6.79)	2.79 (1.10, 7.05)	2.49 (1.07, 5.78)	9.04 (2.31, 35.29)	0.71 (0.14, 3.67)	1.74 (0.67, 4.53)	1.60 (0.27, 9.45)	1.66 (0.18, 15.45)	9.43 (1.24, 71.68)	0.75 (0.10, 5.56)	1.36 (0.39, 4.82)	0.65 (0.10, 4.22)	Non immunosuppressive therapy

The table shows comparisons of complete remission among different treatment strategies. Data are odds ratio with 95% confidence interval within brackets. The table should be read from left to right. Risk estimate is for the column-defining treatment compared to the row-defining treatment. An odds ratio < 1 indicates the column treatment is associated with lower odds of complete remission than the row treatment

*ACTH* adrenocorticotropic hormone

**Table 2:** Network estimates (odds ratios and 95% CI) of effects of treatment on serious adverse events

Cyclophosphamide	1.76 (0.45, 6.83)	0.68 (0.25, 1.87)	0.53 (0.15, 1.86)	0.71 (0.11, 4.64)	0.40 (0.11, 1.49)	0.69 (0.10, 4.84)	1.10 (0.15, 8.40)	0.31 (0.03, 3.44)	0.30 (0.10, 0.92)
0.57 (0.15, 2.20)	Chlorambucil	0.39 (0.10, 1.48)	0.30 (0.06, 1.52)	0.40 (0.04, 4.09)	0.23 (0.05, 1.16)	0.39 (0.04, 4.20)	0.63 (0.05, 7.19)	0.17 (0.01, 2.82)	0.17 (0.04, 0.67)
1.46 (0.54, 3.98)	2.57 (0.67, 9.81)	Calcineurin inhibitor	0.78 (0.24, 2.54)	1.04 (0.12, 8.72)	0.59 (0.17, 2.09)	1.00 (0.11, 9.02)	1.61 (0.17, 15.51)	0.45 (0.03, 6.07)	0.43 (0.16, 1.17)
1.87 (0.54, 6.51)	3.29 (0.66, 16.50)	1.28 (0.39, 4.17)	Rituximab	1.33 (0.14, 12.65)	0.75 (0.16, 3.53)	1.29 (0.13, 13.03)	2.07 (0.19, 22.34)	0.57 (0.04, 8.64)	0.56 (0.16, 1.97)
1.41 (0.22, 9.23)	2.48 (0.24, 25.19)	0.97 (0.11, 8.12)	0.75 (0.08, 7.19)	Calcineurin inhibitor plus rituximab	0.57 (0.06, 5.60)	0.97 (0.06, 14.56)	1.56 (0.10, 24.72)	0.43 (0.02, 9.25)	0.42 (0.05, 3.75)
2.48 (0.67, 9.13)	4.36 (0.86, 22.03)	1.70 (0.48, 6.02)	1.33 (0.28, 6.21)	1.76 (0.18, 17.31)	Mycophenolate mofetil	1.70 (0.16, 17.82)	2.74 (0.25, 30.52)	0.76 (0.05, 11.81)	0.74 (0.22, 2.51)
1.45 (0.21, 10.25)	2.56 (0.24, 27.60)	1.00 (0.11, 8.94)	0.78 (0.08, 7.89)	1.03 (0.07, 15.50)	0.59 (0.06, 6.14)	Mizoribine	1.61 (0.10, 26.83)	0.45 (0.02, 9.99)	0.43 (0.05, 4.12)
0.91 (0.12, 6.88)	1.59 (0.14, 18.28)	0.62 (0.06, 5.96)	0.48 (0.04, 5.24)	0.64 (0.04, 10.19)	0.37 (0.03, 4.07)	0.62 (0.04, 10.39)	Steroid	0.28 (0.07, 1.03)	0.27 (0.03, 2.74)
3.25 (0.97, 13.36)	5.73 (0.36, 92.38)	2.23 (0.16, 30.10)	1.74 (0.12, 26.14)	2.31 (0.11, 49.22)	1.31 (0.08, 20.34)	2.24 (0.10, 49.3)	3.59 (0.97, 13.36)	Placebo	0.97 (0.07, 13.48)
3.36 (1.09, 10.35)	5.92 (1.50, 23.39)	2.30 (0.85, 6.19)	1.80 (0.51, 6.36)	2.38 (0.27, 21.29)	1.36 (0.40, 4.60)	2.31 (0.24, 21.98)	3.71 (0.36, 37.75)	1.03 (0.07, 14.37)	Non immunosuppressive therapy

The table shows comparisons of serious adverse events among different treatment strategies. Data are odds ratio with 95% confidence interval within brackets. Odds ratio higher than one favour the column-defining treatment. The table should be read from left to right. Risk estimate is for the column-defining treatment compared to the row-defining treatment. An odds ratio < 1 indicates the column treatment is associated with lower odds of serious adverse events than the row treatment

#### Complete remission

Twenty-six studies including 1475 patients reported complete remission (Fig. 2) [10, 12, 14, 26, 31, 33, 36, 39, 40, 42, 47, 50, 53–55, 57, 58, 60–64, 68, 71, 73, 74]. It was uncertain whether cyclophosphamide had different odds of inducing complete remission when compared with chlorambucil (OR 1.13, CI 0.46–2.75, low certainty), rituximab (OR 0.35, CI 0.10–1.24, low certainty), mycophenolate mofetil (OR 1.81, CI 0.69–4.71, low certainty), calcineurin inhibitors (OR 1.26, CI 0.61–2.63, low certainty), steroid monotherapy (OR 2.31, CI 0.62–8.52, low certainty) (Table 1). Cyclophosphamide was probably more effective at inducing complete remission than non-immunosuppressive therapy (OR 3.14, CI 1.46–6.79, moderate certainty) and calcineurin inhibitor plus rituximab (OR 4.45, CI 1.04–19.10, low certainty).

#### Partial remission

Twenty-six studies including 1354 patients reported partial remission (Fig. 2) [10, 12, 14, 26, 31, 33, 36, 39, 40, 42, 47, 50, 53–55, 57, 58, 60–64, 68, 71, 73, 74]. Whether cyclophosphamide had important different effects on partial remission compared with other immunosuppression strategies was uncertain due to imprecise estimates leading to low or very low-certainty estimates (Supplementary Table 8.2). However, cyclophosphamide was probably more effective in inducing partial remission than non-immunosuppressive therapy (OR 2.17, CI 1.06–4.45, moderate certainty).

#### Progression to kidney failure and doubling of serum creatinine

Fifteen studies including 1014 patients reported progression to kidney failure (Supplementary Fig. 1.2) [12, 14, 31, 32, 36, 41, 44, 46, 47, 57, 58, 60, 62, 70, 73]. There was no evidence of differences between the different treatment strategies in generally low or very low-certainty evidence as there were only few events (Supplementary Table 8.4). Cyclophosphamide probably had a higher odds of progression to kidney failure compared to chlorambucil (OR 4.99, CI 1.06–23.56, low certainty).

Ten studies including 528 patients reported doubling of serum creatinine (Supplement Fig. 1.3) [10, 29, 36, 44, 51, 57, 58, 63, 65, 70]. Whether there were differences in doubling of serum creatinine between cyclophosphamide and other immunosuppression strategies or non-immunosuppressive therapy care was uncertain (Supplementary Table 8.5).

#### Glomerular filtration rate

Four studies including 131 patients reported end of treatment glomerular filtration rate (Supplementary Fig. 1.4) [34, 45, 50, 68]. It was uncertain whether any treatment strategies had different effects on glomerular filtration rate (low certainty) (Supplementary Table 8.6).

#### Proteinuria

Fourteen studies including 573 patients reported end of treatment proteinuria (Supplementary Fig. 1.5) [24–26, 30, 32, 34, 41, 44, 50, 55, 58, 63, 64, 71]. There was uncertainty whether treatment strategies had different effects on proteinuria (Supplementary Table 8.7).

#### Serious adverse events

Eighteen studies including 1318 patients reported serious adverse events (Supplementary Fig. 1.6) [11–14, 30, 31, 34, 39, 40, 42, 44, 46, 57, 58, 63, 70, 71, 73]. Cyclophosphamide probably caused more serious adverse events than non-immunosuppressive therapy (OR 3.36, CI 1.09–10.35, moderate certainty) (Table 2). It was uncertain whether cyclophosphamide had different risks of serious adverse events compared to other treatment strategies.

#### Discontinuation of treatment

Eighteen studies including 1090 patients reported discontinuation of treatment due to adverse events (Supplementary Fig. 1.7) [13, 24, 25, 30, 31, 33, 34, 38–40, 42, 55, 57, 61, 63, 64, 70, 72]. There was no difference in treatment discontinuation from cyclophosphamide when compared to other treatment strategies except chlorambucil (OR 0.17, CI 0.05–0.54, moderate certainty) (Supplementary Table 8.8).

#### Serious infection

Twenty studies including 1338 patients reported serious infection (Supplementary Fig. 1.8) [11–14, 33, 34, 39, 44, 46, 47, 50, 55, 57, 58, 62–64, 71–73]. It was uncertain that any treatment strategies had different effects on serious infection (low certainty) (Supplementary Table 8.9).

#### Bone marrow suppression

Sixteen studies including 840 patients reported bone marrow suppression (Supplement Fig. 1.9) [11, 12, 14, 25, 33, 38, 39, 44, 46, 50, 55, 58, 60, 62, 64, 71]. Cyclophosphamide may have incurred more bone marrow suppression than mycophenolate mofetil (OR 8.93, CI 1.08–73.69, low certainty) and ACTH (OR 25.19, CI 7.39–85.82, low certainty). Whether cyclophosphamide had different effects on bone marrow suppression compared to chlorambucil, calcineurin inhibitor, rituximab, calcineurin inhibitor plus rituximab, mizoribine, azathioprine placebo and non-immunosuppressive therapy was uncertain (Supplementary Table 8.10).

### Subgroup and sensitivity analyses

We conducted a subgroup analysis of complete and partial remission at 6 months. However, analyses for other pre-specified subgroups was not conducted as there were limited studies assigning and reporting those subgroups.

#### Complete remission at 6 months

Twelve studies including 915 patients reported complete remission at 6 months (Supplementary Fig. 1.1) [10, 12–14, 31, 36, 55, 58, 63, 64, 71, 72]. It was uncertain whether cyclophosphamide had different odds of inducing complete remission at 6 months when compared to all other treatment strategies (Supplementary Table 8.1).

#### Partial remission at 6 months

Eleven studies including 784 patients reported partial remission at 6 months (Supplementary Fig. 1.1) [10, 12–14, 36, 55, 58, 63, 64, 71, 72]. Cyclophosphamide may have induced more partial remission at 6 months when compared to steroids (OR 13.57, CI 2.34–78.59, low certainty), placebo (OR 21.11, CI 1.69–263.35, low certainty) and non-immunosuppressive therapy (OR 24.36, CI 2.08–284.87, low certainty) (Supplementary Table 8.3).

## Discussion

Our network meta-analysis demonstrates that, in adults with idiopathic membranous nephropathy, it is uncertain whether rituximab, mycophenolate mofetil, calcineurin inhibitor or steroid monotherapy have different effects on inducing complete disease remission or preventing kidney failure compared to cyclophosphamide. There were few robust data for treatment-related adverse events and a range of other safety and efficacy outcomes. Additionally, the comparative effects of cyclophosphamide and other immunosuppression strategies on surrogate kidney outcomes including doubling of serum creatinine, proteinuria and estimated glomerular filtration rate were uncertain and require evaluation in further randomized trials. Notably, due to the lack of properly powered head-to-head trials, comparative data for cyclophoshamide and rituximab were largely limited to indirect meta-analysis.

Our study differs from a previous network meta-analysis on idiopathic membranous nephropathy published in 2019, which compared 13 immunosuppressive agents against non-immunosuppressive therapy in 48 trials involving 2736 participants [76]. This study showed that most treatment strategies, except for leflunomide, mizoribine and steroid monotherapy, were significantly more likely to result in total remission compared with non-immunosuppressive therapies. Since the publication of this network meta-analysis, which included randomized controlled trials reported up to the 1st of February 2018, several key additional randomized controlled trials such as MENTOR, STARMEN and RICYCLO have been published [12–14]. These trials have provided more data for comparing rituximab, which is a treatment of much interest among researchers in the field of membranous nephropathy.

The latest KDIGO Guideline published in 2021 makes a strong (level 1B) recommendation for the use of either rituximab or cyclophosphamide and steroids for 6 months or tacrolimus-based treatment for at least 6 months in patients with idiopathic membranous nephropathy and at least one risk factor for disease progression [5]. For those at very high risk, cyclophosphamide and steroids are recommended [5]. This recommendation is not supported by the present network meta-analysis which demonstrated uncertainty in the comparative effects of cyclophosphamide with other immunosuppression therapies, including rituximab, in the treatment of idiopathic membranous nephropathy. Until higher certainty evidence is generated, it is not unreasonable to treat patients at higher risk of disease progression with cyclophosphamide-, rituximab- or tacrolimus-based immunosuppression in line with KDIGO recommendations, but clinicians should recognize that the GRADE assessment of the evidence underpinning this recommendation is closer to 2C than 1B.

The present analysis is based on a highly sensitive systematic literature search and has been conducted and reported using recommended methodologies including GRADE assessments of evidence certainty. Importantly, it has highlighted that the evidence for immunosuppressive regimens in membranous nephropathy is far less certain than is suggested by the level 1B recommendation made by the KDIGO guidelines. The search for contributing trials was updated to include newer agents such as rituximab. However, the analysis has limitations. The studies were of variable methodological quality such that a minority were deemed as being at low risk of bias. There was also considerable heterogeneity with respect to participant characteristics (e.g. baseline proteinuria and kidney function), background treatment prior to intervention, interventions (e.g. cyclophosphamide versus chlorambucil, oral versus intravenous, cyclic versus continuous), outcome definitions (particularly complete and partial remission Supplementary Table 3), follow-up periods (6 months–10 years) and study design. There was heterogeneity in the baseline phospholipase A2 receptor antibody. However, the capacity to explore potential sources of heterogeneity due to these factors was limited by statistical power and the number of reported studies. The degree of heterogeneity also precluded analysis of treatment effects according to the risk of progression. Although the total number of trials was large, individual treatment to treatment comparisons involved small numbers of trials with small numbers of patients, mostly open-label trials with high or unclear risks of bias, few event rates, wide confidence intervals and greatly reduced precision of estimates. These limitations appreciably reduced the certainty of evidence.

This network meta-analysis has included studies over the past 50 years. As a result, there were significant differences in the treatment and baseline characteristics of these patients. The older studies with cyclophosphamide and chlorambucil discouraged the use of renin–angiotensin–aldosterone system (RAAS) blockade [47, 62]. This would have had a significant impact on the rate of spontaneous remission. However, modern-day studies with rituximab used RAAS blockade and allowed time for spontaneous remission before initiating immunosuppressive therapy. Similarly, the characteristics of patients in recent studies have been more complex with older age, greater frailty and worse kidney dysfunction. Most of the older studies had younger patients with relatively preserved kidney function. These era effects challenge the comparison of traditional cyclophosphamide agent-based therapy against rituximab-based treatment and make it difficult to interpret the results of this network meta-analysis.

Future trials in membranous nephropathy should include standardized inclusion criteria, particularly with respect to the risk of progressive disease, degree of proteinuria and level of kidney function, and standardized definitions of complete and partial remission. Trial investigators should additionally consider using anti-PLA2R antibodies as a criterion for stratification. To aid decision-making, trial follow-up should include robust measures of patient-important outcomes including kidney failure and death.

In conclusion, the effects of immunosuppression strategies compared to cyclophosphamide are uncertain in the treatment of adults with idiopathic membranous nephropathy. Given the potential harms of cyclophosphamide, head-to-head trials combined with the exploration of patient preferences about the benefits and harms of treatment strategies could better inform decision-making.

## Supplementary Information

Below is the link to the electronic supplementary material.

Supplementary file1 (DOCX 7254 kb)
