# Supplementary material for: Immunosuppression therapy for idiopathic membranous nephropathy: systematic review with network meta-analysis
Source: J Nephrol. 2022 Feb 23;35(4):1159–70. doi: 10.1007/s40620-022-01268-2 (PMC9107446; doi:10.1007/s40620-022-01268-2)
Supplement: Supplementary file 1 — Supplementary file1 (DOCX 7254 kb) [file 40620_2022_1268_MOESM1_ESM.docx]

# Title page

# Title: Immunosuppression therapy for idiopathic membranous nephropathy: A systematic review and network meta-analysis

# Online Supplement

## Supplementary Methods. Summary of statistical Analysis

First, pairwise random effects meta-analysis was conducted by synthesizing data from trials comparing at least two different immunosuppressive strategies, placebo, standard therapy or no additional therapy. Using a random effects model, different studies were assumed to assess different yet related treatment effects. The relative treatment effects of the competing interventions were estimated using odds ratios for the dichotomous outcomes (complete remission, partial remission, complete or partial remission, end-stage kidney disease, serious adverse events, discontinuation of treatment, serious infection, onset of diabetes mellitus, bone marrow suppression) and mean difference for continuous outcomes (estimated glomerular filtration rate, serum creatinine, proteinuria).

A mean difference equal to zero meant that the compared treatment strategies may have similar effects. An odds ratio below 1 indicated that the treatment was associated with a lower odds of the outcome.

Network meta-analysis was conducted to generate indirect and mixed (direct and indirect) treatment estimates to compare different immunosuppression strategies. Network meta-analysis was used to synthesize direct evidence (from head-to-head trial comparisons) and indirect evidence (estimating the relative effectiveness between pairs of interventions even if these have never been compared directly in trials ^1-3^.

Network meta-analysis was then used in the ranking of interventions.

A key assumption of network meta-analysis is that of consistency; that one can learn about the relative effectiveness between the two compared interventions *indirectly.* Consistency implies that the distribution of effect modifiers is the same across treatment comparisons ^4^.For example, potential effect modifiers are similar across all included trials. The variables were all considered when assessing this assumption. Consistency implied that the studies comparing different drugs (for example, alkylating agents plus corticosteroids versus non-immunosuppressive therapy or calcineurin inhibitors versus rituximab) were similar in terms of patient and study characteristics.

Frequentist random effects network meta-analysis was performed in Stata using the mvmeta and network commands and self-prgrammed Stata routines available at <http://www.mtm.uoi.gr/index.php/stata-routines-for-network-meta-analysis> ^5,6^. The ranking probabilities for all treatments being at each possible rank for each intervention were estimated ^7^. The treatment hierarchy of the competing interventions was obtained using rankograms, the surface under the cumulative ranking curve (SUCRA) and mean ranks ^5,8^. The SUCRA expressed as a percentage the efficacy or safety of each intervention relative to an imaginary intervention that was always the best without uncertainty. A SUCRA of 80% meant that the intervention of interest was associated with 80% of the effectiveness of this imaginary intervention.

In network meta-analysis, heterogeneity was assumed to be the same for all treatment comparisons within a single network. The restricted maximum likelihood (REML) method was used to estimate the heterogeneity assuming a common estimate for the heterogeneity variance across the different drug classes. The assessment of heterogeneity in the entire network was based on the magnitude of the heterogeneity variance parameter estimated from the network meta-analysis models. The heterogeneity variance was compared with the empirical distribution derived by Spiegelhalter et al ^9^.

Disagreement between direct and indirect evidence challenges the consistency assumption. To evaluate for the presence of inconsistency, the loop-specific approach was used to evaluate the difference between direct and indirect estimates for a specific comparison in the loop (consistency factor). A common heterogeneity estimate within each loop was assumed. The results of this approached were also presented graphically in a forest plot in which the ratio of odds ratios between the direct indirect evidence was plotted. A ratio with a 95% confidence interval excluding 1 indicated evidence of statistical inconsistency between a direct treatment and an indirect estimate derived from treatments within the same triangular or quadratic loop of evidence ^10,11^. To check the assumption of consistency in the entire network, the ‘design-by-treatment’ model was used as has been described by Higgins and colleagues ^4^. This method accounts for different potential sources of inconsistency that can occur when studies with different designs (two-arm trials versus three arm trials) give different results as well as differences between direct and indirect evidence. Using this method, the presence of any inconsistency from sources in the entire network was inferred based on a chi-square test.

**Meta-regression and sensitivity analyses.**

This systematic review was performed and reported according to the Preferred Reporting Items for Systematic Reviews and Meta-Analyses (PRISMA) statement incorporating network meta-analyses of health care interventions ^12^.

The certainty (confidence in evidence, quality of evidence) in estimates from the network meta-analysis was assessed using the Grading of Recommendations Assessment, Development and Evaluation (GRADE) working group ^13^.

## Supplementary Table 1 Electronic search strategies

| **Database** | **Search items** |
| --- | --- |
| EMBASE: 1974 to 23^rd^ July 2021 | 1. exp membranous glomerulonephritis/ or Primary membranous nephropathy.tw. or Primary membranous glomerulonephritis.tw. or Idiopathic membranous glomerulonephritis.tw. or Idiopathic membranous nephropathy.tw. or MGN.tw. or IMN.tw. or exp nephrotic syndrome/ or exp glomerulonephritis/ or Primary membranous glomerulonephropathy.tw. or exp glomerulopathy/ 2. Idiopathic membranous glomerulonephropathy.tw. or Membranous glomerulonephropathy.tw. or Membranous glomerulopathy.tw. or Primary membranous glomerulopathy.tw. or Idiopathic membranous glomerulopathy.tw. or exp proteinuria/ or exp phospholipase A2/ or exp phospholipase A2 receptor/ or PLA2R.tw. or PLA2R Antibody.tw. or PLA2R Ab.tw. 3. 1 or 2 4. exp cyclophosphamide/ or Cytoxan.tw. or Endoxan.tw. or exp chlorambucil/ or Leukeran.tw. or exp prednisone/ or exp corticosteroid/ or exp meprednisone/ or Ponticelli.tw. or exp methylprednisolone/ or exp prednisolone/ or Ponticelli regimen.tw. or Modified Ponticelli.tw. or Modified Ponticelli Regimen.tw. or exp tacrolimus/ or Prograf.tw. or exp cyclosporin/ or exp cyclosporine/ or exp cyclosporin A/ or Neoral.tw. or Ciclosporin.tw. or exp calcineurin inhibitor/ 5. exp mycophenolate mofetil/ or exp mycophenolic acid 2 morpholinoethyl ester/ or exp mycophenolic acid/ or MMF.tw. or Cellcept.tw. or Myfortic.tw. or exp immunosuppressive treatment/ or exp immunosuppressive agent/ or Adrenocorticotrophic hormone.tw. or exp corticotropin/ or ACTH.tw. or Acthar gel.tw. or exp rituximab/ or Mabthera.tw. or Rituxan.tw. or exp monoclonal antibody/ or exp CD20 antigen/ or exp CD20 antibody/ 6. 4 or 5 7. exp controlled clinical trial/ or exp clinical trial/ or exp controlled study/ or exp randomized controlled trial/ 8. 3 and 6 and 7 9. limit 8 to human |
| MEDLINE: 1946 to 23^rd^ July 2021 | 1. exp Glomerulonephritis, Membranous/ or Primary membranous nephropathy.tw. or Primary membranous glomerulonephritis.tw. or Idiopathic membranous glomerulonephritis.tw. or Idiopathic membranous nephropathy.tw. or MGN.tw. or IMN.tw. 2. Primary membranous glomerulonephropathy.tw. or Idiopathic membranous glomerulonephropathy.tw. or Membranous glomerulopathy.tw. or Primary membranous glomerulopathy.tw. or Idiopathic membranous glomerulopathy.tw. 3. exp GLOMERULONEPHRITIS/ or exp Nephrotic Syndrome/ or exp PROTEINURIA/ or exp Receptors, Phospholipase A2/ or PLA2R.tw. or PLA2R Antibody.tw. or PLA2R Ab.tw. 4. 1 or 2 or 3 5. exp CYCLOPHOSPHAMIDE/ or Cytoxan.tw. or Endoxan.tw. or exp CHLORAMBUCIL/ or Leukeran.tw. or Ponticelli.tw. or Ponticelli regimen.tw. or Modified Ponticelli.tw. or Modified Ponticelli regimen.tw. or exp PREDNISOLONE/ or exp Glucocorticoids/ 6. exp CYCLOSPORINE/ or Ciclosporin.tw. or Neoral.tw. or exp TACROLIMUS/ or Prograf.tw. or exp Calcineurin Inhibitors/ or exp Mycophenolic Acid/ or Mycophenolate sodium.tw. or MMF.tw. or Cellcept.tw. or Myfortic.tw. 7. exp Adrenocorticotropic Hormone/ or ACTH.tw. or Acthar gel.tw. or exp RITUXIMAB/ or Mabthera.tw. or Rituxan.tw. or exp Antibodies, Monoclonal/ or exp ANTIGENS, CD20/ or exp Immunosuppressive Agents/ 8. 5 or 6 or 7 9. exp Randomized Controlled Trial/ or exp Clinical Trial/ or exp Controlled Clinical Trial/ or exp Clinical Trials as Topic/ or exp Random Allocation/ or exp Double-Blind Method/ or exp Single-Blind Method/ 10. 4 and 8 and 9 11. limit 10 to humans |
| CENTRAL: issue 7, 2021 | 1. exp Glomerulonephritis, Membranous/ or Primary membranous nephropathy.tw. or Idiopathic membranous glomerulonephritis.tw. or Idiopathic membranous.tw. or glomerulonephropathy.tw. or Idiopathic membranous nephropathy.tw. or MGN.tw. or IMN.tw. or Membranous nephropathy.tw. or glomerulonephropathy.tw. or Membranous glomerulonephropathy.tw. or Membranous glomerulopathy.tw. 2. exp Glomerulonephritis/ or exp Proteinuria/ or exp Nephrotic Syndrome/ or exp Receptors, Phospholipase A2/ 3. 1 or 2 4. exp Cyclophosphamide/ or Cytoxan.tw. or Endoxan.tw. or exp Chlorambucil/ or Leukeran.tw. or Ponticelli.tw. or Ponticelli regimen.tw. or Modified Ponticelli.tw. or Modified Ponticelli regimen.tw. or exp Methylprednisolone/ or exp Prednisolone/ or exp Glucocorticoids/ or exp Tacrolimus/ or exp Cyclosporine/ or Ciclosporin.tw. or Neoral.tw. or Prograf.tw. or Calcineurin inhibitor.tw. 5. Mycophenolate mofetil.tw. or Mycophenolate sodium.tw. or exp Mycophenolic Acid/ or Cellcept.tw. or Myfortic.tw. or MMF.tw. or exp Adrenocorticotropic Hormone/ or ACTH.tw. or Acthar gel.tw. or Rituximab.tw. or Mabthera.tw. or Rituxan.tw. or exp Antibodies, Monoclonal/ or exp Antigens, CD20/ or exp Adrenal Cortex Hormones/ or exp Immunosuppressive Agents/ 6. 4 or 5 7. 3 and 6 |

## Supplementary Table 2: Characteristics of included studies

| **Study** | **Inclusion Criteria** | **N** | **Active Treatment** | **Control** | **Baseline kidney function** | **Baseline Proteinuria** | **Follow up** | **Age**  **(years, mean)** |
| --- | --- | --- | --- | --- | --- | --- | --- | --- |
| BMJ 1971 ^14^ | Any age  Proteinuria > 1g/24 hour  Creatinine clearance >20 mL/min/1.73 m^2^ | 14 | Azathioprine 2.5mg/kg/d PLUS prednisone 20mg/kg or 0.5mg/kg/day for 8 weeks | No drug | Creatinine clearance (mL/min/1.73 m^2^)  Active 65.8 + 20.5  Control 105.3 + 6.3 | 24 hour proteinuria (g/24 hour)  6.4 + 1.6 | 6 months | NA |
| Silverberg 1976 ^15^ | Age 18-70 years  Biopsy-proven idiopathic membranous nephropathy in last 6months  Proteinuria > 3g/24 hour  Creatinine clearance >50mL/min/1.73m^2^ | 9 | Azathioprine 2.5mg/kg/day | Placebo | Creatinine clearance (mL/min/1.73m^2^)  Active 95 + 37  Control 74 + 22  Serum creatinine (mg/dL)  Active 1.1 + 0.4  Control 1.5 + 0.5 | 24 hour proteinuria (g/24 hour)  Active 12.2 + 4.9  Control 9.1 + 5.9 | 12 months | Active 41  Control 45 |
| Coggins 1979 ^16^ | Age 16-65 years  Biopsy-proven idiopathic membranous nephropathy  Proteinuria >3.5g/24 hour  Creatinine clearance>60mL/min/1.73m^2^ | 72 | Prednisone 125mg/day (45 to 80Kg patient)  100mg (<45kg patient) initially for 8 weeks and then tapered based on response | Placebo | Serum creatinine (mg/dL)  Active 1.1 + 0.2  Control 1.0 + 0.2 | 24 hour proteinuria (g/24 hour)  Active 9.4 + 6  Control 8.3 + 4 | 36 months | NA |
| Badri 2013 ^17^ | Age 18-65 years  Biopsy proven membranous nephropathy  Proteinuria >500mg/day | 18 | Pentoxifylline 400mg orally two or three times daily for 6 months | Matching placebo for 6 months | eGFR (mL/min)  Active 71.2+30.6  Control 75.3+17.2 | 24 hour proteinuria (mg/24 hour)  Active 2450.5 (1150-7028)  Control 1648.5 (630-3000) | 6 months | Active 36.2  Control 40.8 |
| Cattran 2001 ^18^ | Age 18-70 years  Failed to achieve remission after 8 weeks prednisone at >1mg/kg/day  Proteinuria >3.5g/24 hour  Creatinine clearance>42mL/min/1.73m^2^  BP < 135/85 mmHg  Kidney biopsy within three years | 51 | Cyclosporine A 3.5 mg/kg/d in 2 divided doses. Dose adjusted for trough level 125-225 mg/L  PLUS  Prednisone 0.15mg/kg/d up to 15 mg. This was reduced after 26 weeks by thirds at 4-week intervals and was stopped after 8 weeks. | Placebo | Creatinine clearance (mL/min/1.73 m²)  Active 90 ± 27  Control 95 ± 37  Serum creatinine (mg/dL)  Active 1.3 ± 0.5  Control 1.1 ± 0.3 | 24 hour proteinuria (g/24 hour)  Active 9.7 ± 5.3  Control 8.8 ± 4.7 | 78 weeks | Active 47 ± 11  Control 49 ± 14 |
| Xu 2013 ^19^ | Age 18-80 years Biopsy-proven idiopathic membranous nephropathy with nephrotic syndrome;  No immunosuppressive treatment in the previous 6 months | 100 | Tacrolimus 0.1 mg/kg/day plus Prednisone 0.5 mg/kg/day | Cyclophosphamide 0.5-0.75g/m^2^/month for 9 months plus Prednisone 1 mg/kg/day | Serum creatinine (mg/dL)  Active 0.88 ± 0.26  Control 1.04 ± 0.53 | 24 hr proteinuria (g/24 hour)  Active 5.39 + 2.51  Control 5.10 + 2.20 | 18 months | Active 57.8 ± 14.8  Control 56.3 ± 13.2 |
| Howman 2013  ^20^ | Age 18-75 years  Biopsy-proven idiopathic membranous nephropathy  Serum or plasma creatinine <300 μmol/L together with >20% decline in excretory kidney function | 106 | Months 1, 3, 5: intravenous Methylprednisolone 1g/day for 3 consecutive days then oral prednisone 0.5 mg/kg/d for 28 days plus Months 2, 4, 6: Chlorambucil starting at 0.15 mg/kg/day  Cyclosporine A for 12 months starting at 5mg/kg/day dose adjusted to achieve a trough level of 100-200 ug/L | Supportive therapy alone | Creatinine clearance (mL/min/1.73 m^2^)  Chlorambucil 50 + 16  Cyclosporine A 49 + 18  Control 50 + 20 | 24 hour proteinuria (g/24 hour)  Chlorambucil 10.1 + 5.3  Cyclosporine A 6.8 + 4.7  Control 9.1 + 5.3 | Until primary endpoint achieved (further 20% reduction in kidney function from baseline)  OR minimum of 3 years If endpoint not reached | Chlorambucil 58 ± 12  Cyclosporine A 58 ± 11  Control 56 ± 16 |
| Naumovic 2011  ^21^ | Biopsy-proven idiopathic membranous nephropathy and nephrotic syndrome or kidney insufficiency | 23 | Cyclosporine A 3 mg/kg/d. Dose adjusted to achieve trough levels 80-100 ng/mL plus  prednisone 0.5 mg/kg/d for 8 weeks. Dose gradually reduced to 5-10mg/day, and remained unchanged until the end of the treatment. | Azathioprine 1.5-2 mg/kg for 6 months, then 50mg/day plus  prednisone 0.5 mg/kg/day for 8 weeks. Dose was gradually reduced to 5-10mg/d, and remained unchanged until the end of the treatment | Serum creatinine (umol/L)  Active 124.5 ± 75.9 Control 120.5 ± 46.5  Glomerular filtration rate (mL/min/1.73 m^2^)  Active 80.7 ± 27.5 Control 76.2 ± 31.3 | 24 hour Proteinuria (g/24 hour):  Active group 11.6 ± 4.7; Control group 7.0 ± 2.7 | 36 months | Active 39.2 ± 13.1  Control 47.5 ± 8.2 |
| Kosmadakis  2010  ^22^ | Biopsy-proven idiopathic membranous nephropathy with nephrotic syndrome for over 6 months and no apparent secondary cause of membranous nephropathy. | 28 | Cyclosporine A 3-3.5 mg/kg/d (adjusted for trough levels 100-120ug/L) plus oral methylprednisolone 12.5mg/kg/d for 9 months  Cyclosphosphamide 2 mg/kg/d  plus oral methylprednisolone 1.5 mg/kg/48 hours for 9 months | Lisinopril for 9 months (not prescribed to other two groups)  Low sodium diet, loop diuretics, anti-hypertensives if indicated | Glomerular filtration rate (mL/min/1.73m^2^)  Cyclosporine A 81.6 ± 8  Cyclophosphamide 51.5 ± 7 Lisinopril 65.7 ± 5.6 | 24 hour Proteinuria (g/24 hour)  Cyclosporine A 6.6 ± 1.0  Cyclophosphamide 7.0 ± 0.7  Lisinopril 5.2 ± 0.8 | 9 months | Cyclosporine A 50.5 ± 4.9  Cyclophosphamide 55.4 ± 2.8  Lisinopril 51.8 ± 5.4 |
| Chen 2010  ^23^ | Age 18-70 years; Biopsy-proven Idiopathic membranous nephropathy (stage I–III) with nephrotic syndrome Initial serum creatinine <221umol/L  No immunosuppressive treatment in previous 3 months | 73 | Tacrolimus 0.1 mg/kg/day plus prednisone 1mg/kg/d for 4 weeks, tapered gradually, and discontinued by 8 months. | Cyclophosphamide 100mg/d for 4 months then reduced by 50 mg/day plus oral prednisone 1mg/kg/d for 4 weeks, tapered gradually, and discontinued by 8months. | Serum creatinine (umol/L)  Active 75.7 ± 22.4  Control 85.0 ± 37.5  Glomerular filtration rate (mL/min/1.73 m^2^)  Active 105.5 ± 28.7  Control 97 ± 34.3 | Proteinuria (g/24 hour)  Active group 7.71 ± 3.93; Control group 7.28 ± 3.91 | 12 months | Active group 47.2±11.9; Control group 48.6±11.6 |
| Nayagam 2008  ^24^ | Adult patients  Biopsy-proven idiopathic membranous nephropathy with nephrotic syndrome.  Estimated glomerular filtration rate >60mL/min/1.73 m^2^ | 21 | Mycophenolate mofetil 2 g/day in 2 divided doses for 6 months plus prednisolone 0.5 mg/kg/d for 8-12 week. | Months 1, 3, 5: intravenous methylprednisolone 1g/day for 3 consecutive days then oral prednisone 0.5mg/kg/day for 27 days  Months 2, 4, 6: oral cyclophosphamide 2mg/kg/day for 30 days. | Estimated glomerular filtration rate (mL/min/1.73 m^2^)  Active 86 ± 12.6  Control 82 ± 11.8 | Urine protein:creatinine ratio (mg/mg)  Active 4.68 ± 1.82  Control 4.95 ± 1.65 | Active 18.2 months  Control 16.1 months | NA |
| Dussol 2008  ^25^ | Age >18 years; Biopsy-proven idiopathic membranous nephropathy;  Nephrotic syndrome and serum creatinine level< 2.26 mg/dL | 36 | Mycophenolate mofetil  250 mg/day, progressively increased by 250 mg every other day to 2 g/day for 12 months. | Supportive therapy  Renin-angiotensin blockers, statins, low-salt and low-protein diet, and diuretics in case of oedema | Serum creatinine (mg/dL)  Active 1.01 ± 0.34 Control 1.09 ± 0.39  Glomerular filtration rate (mL/min/1.73m^2^)  Active 92.1 ± 29.8 Control 80.7 ± 25.4 | 24 hour Proteinuria (g/24 hour)  Active group 6.2 ± 3.5); Control group 9.5 ± 5.8 | 12 months | Active 47.8 ± 15.2  Control 55.9 ± 15.2 |
| Praga 2007 ^26^ | Age 18-70 years  Biopsy-proven idiopathic membranous nephropathy  Nephrotic range proteinuria >3.5g/d;  Albumin <3g/dL; estimated glomerular filtration rate ≥50 mL/min/1.73m^2^ | 48 | Tacrolimus 0.05 mg/kg/d, divided into 2 daily doses at 12-h interval. Doses adjusted to achieve 12-h trough level 3-5 ng/mL. Treated for 12 months and then gradually tapered over 6 months. | Supportive therapy | Serum creatinine (mg/dL)  Active 0.98 ± 0.2  Control 1.1 ± 0.3  Estimated glomerular filtration rate (mL/min/1.73m^2^) Active 104 ± 26  Control 107 ± 63 | 24 hour Proteinuria (g/24 hour)  Active 7.2 ± 3.3  Control 8.4 ± 5.4 | 30 months | Active 43.7 ± 12.1  Control 50.1 ± 12.2 |
| Jha 2007 ^27^ | Age >16 years  Biopsy-proven idiopathic membranous nephropathy with nephrotic syndrome for at least 6 months | 93 | Months 1, 3, 5: IV methylprednisolone 1g/day for 3 consecutive days then oral prednisone 0.5 mg/kg/d for 27 days  Months 2, 4, 6: Oral cyclophosphamide 2mg/kg/day for one month | Supportive therapy (dietary sodium restriction, diuretics, and antihypertensive agents) | Serum creatinine (mg/dL)  Active 1.21 ± 0.31  Control 1.17 ± 0.22  Glomerular filtration rate (mL/min) Active 89 ± 26  Control 84 ± 22 | 24 hour Proteinuria (g/24 hour)  Active 6.11 ± 2.5  Control 5.91 ± 2.2 | 120 months | Active 38.0 ± 13.6  Control group 37.2 ± 12.4 |
| Chan 2007  ^28^ | Age 18–65 years  Biopsy-confirmed membranous nephropathy diagnosed in past 6 months Proteinuria of ≥3 g/day without spontaneous improvementSerum creatinine <300 umol/L; treatment-naive | 20 | Mycophenolate mofetil 1 g twice a day for 6 months plus oral prednisolone started at 0.8 mg/kg/day, then tapered by 5 mg/day for 6 months | Months 1, 3, 5: IV Methylprednisolone 1g/day for 3 consecutive days then oral prednisone 0.4mg/kg/day for 3 weeks then 0.2 mg/kg/d until end of month  Months 2, 4, 6: oral chlorambucil 0.2 mg/kg/day for one month. | Serum creatinine (µmol/L)  Active 103.3 ± 48.7  Control 85.7 ±31.8  Glomerular filtration rate (mL/min/1.73m^2^)  Active 87.1 ± 38.5; Control 101.8 ± 40.6 | 24 hour Proteinuria (g/24 hour)  5.7 ± 2.7 | 15 months | 49.5 ± 13.5 |
| Ponticelli  1998  ^29^ | Age 14-65 years  Biopsy-proven idiopathic membranous nephropathy with nephrotic syndrome  Plasma creatinine of <1.7mg/dL (or <150 umol/L) | 95 | Months 1, 3, 5: IV Methylprednisolone 1g/day on 3 consecutive days and then oral prednisone 0.4 mg/kg/day for 27 days  Months 2, 4, 6: oral cyclophosphamide 2.5mg/kg/day for one month | Months 1, 3,5: IV Methylprednisolone 1g/day on 3 consecutive days then oral prednisone 0.4mg/kg/d for 27 days.  Months 2, 4, 6: oral chlorambucil 0.2mg/kg/day for one month | Serum creatinine (mg/dL)  Cyclophosphamide 1.04 ± 0.27  Chlorambucil 1.06 ± 0.27 | 24 hour Proteinuria (g/24 hour)  Cyclophosphamide 6.85 + 3.51  Chlorambucil 7.96 + 5.19 | Median  Cyclophosphamide 42 months  Chlorambucil 36 months | Median and range  Cyclophosphamide (48, 17-55)  Chlorambucil (50, 18-65) |
| Branten 1998  ^30^ | Age ≥18 years  Patients with biopsy-proven idiopathic membranous nephropathy with nephrotic syndrome and deteriorating kidney function | 32 | Oral cyclophosphamide 1.5-2mg/kg/day for 1 year plus steroids in a comparable dose | Months 1, 3, 5: IV methylprednisolone 1g daily for 3 consecutive days, then oral prednisone 0.5mg/kg/day for 27 days  Months 2, 4, 6: Chlorambucil 0.15 mg/kg/day for one month | Serum creatinine (umol/L)  Cyclophosphamide 274 + 126  Chlorambucil 219 + 73  Glomerular filtration rate (mL/min/1.73 m^2^)  Cyclophosphamide 43 + 23  Chlorambucil 46 + 17 | 24 hour Proteinuria (g/24 hour)  Cyclophosphamide 11 + 5.3  Chlorambucil 9 + 2.6 | Median  Active 26 months  Control 38 months | Cyclophosphamide 53 + 14  Chlorambucil 51 + 12 |
| Austin 1996  ^31^ | Patients with idiopathic membranous nephropathy | 31 | Intravenous cyclophosphamide 0.5g/m² every other month  Prednisone 40mg/m² every other day for 2 months tapered to 10 mg/m² | Oral prednisone 40mg/m² every other day for 2 months tapered to 10 mg/m² | Glomerular filtration rate  Range (mL/min/1.73m^2^): 24-156 | NA | NA | NA |
| Ponticelli 1995  ^32^ | Adult patients  Biopsy-proven idiopathic membranous nephropathy Nephrotic syndrome | 81 | Months 1, 3, 5: Intravenous Methylprednisolone 1g daily for 3 consecutive days then oral prednisolone 0.5mg/kg/day given for 27 days.  Months 2, 4, 6: oral chlorambucil 0.2 mg/kg/day for one month. | No specific immunosuppressive treatment | Serum creatinine (umol/L)  Active 93.8 ± 21.5  Control 93.1 ± 25.3 | 24 hour Proteinuria (g/24 hour)  Active 6.18 ± 2.98  Control 5.30 ± 2.84 | 120 months | Median (range)  Active 43.5 (15-70)  Control 42 (16-74) |
| Cattran 1995  ^33^ | Age 18-65 years;  Recent (≤24 months) biopsy-proven membranous nephropathy.  Proteinuria ≥1g/day.  Declining kidney function and no spontaneous remission | 17 | Cyclosporine A  100 mg/mL initiated at 3.5 mg/kg/d taken in 2 divided doses, Adjusted to 12-hour trough level 110-170 ng/mL. | Placebo | Serum creatinine (umol/L)  Active 186 ± 65  Control 204 ± 81  Glomerular filtration rate (mL/min/1.73 m^2^)  Active 51 ± 20  Control 46 ± 16 | 24 hour Proteinuria (g/24 hour)  Active 11.5, 9-18  Control 12.8, 4-21 | 21 months | Median (range)  Active 44, 22-59  Control 40, 20-61 |
| Reichert 1994  ^34^ | Age≥18 years Biopsy-proven idiopathic membranous nephropathy with nephrotic syndrome and deteriorating kidney function | 18 | Intravenous cyclophosphamide 750 mg/m² once every month for 6 months  Intravenous methylprednisolone 1g/day for 3 consecutive days at the beginning of months 1, 3, 5 | Months 2, 4, 6: oral chlorambucil 0.15 mg/kg/d  Months 1, 3, 5: intrevenous methylprednisolone 1g/d for 3 consecutive days then PO prednisone 0.5mg/kg/d for 27 days | Serum creatinine (umol/L)  Active 218 + 85  Control 260 + 112 | 24 hour Proteinuria (g/24 hour)  Active 9.8 + 4.8  Control 8.5 + 2.5 | Median 15 months | Active (49,24-65)  Control (45, 31-65) |
| Falk 1992  ^35^ | Age 16-70 years.  Biopsy-proven progressive idiopathic membranous nephropathy with either deteriorating kidney function or persistent proteinuria associated with morbid complications | 26 | Intravenous cyclophosphamide 0.5g/m^2^ for 6 months in conjunction with a 3-day course of pulse methylprednisolone (7mg/kg) and then oral prednisolone 1mg/kg every other day for 2 months and then tapered 25%/dose/week over next 4 weeks | Oral prednisone 2mg/kg on alternate days for 8 weeks, and then tapered by 25%/dose/wk over 4 weeks | Serum creatinine (mg/dL)  Active group 2.3 ± 1.0; Control group 2.7 ± 1.6 | 24 hour Proteinuria (g/24 hour)  Active 12.4 ± 9.9  Control 11.1 ± 6.7 | Mean 29.2 ± 17.1 months  Active 31.9 ± 16.6 months  Control 26.5 ± 15 months | Active 43.3 ± 14.8  Control 46.0 ± 13.7 |
| Cameron 1990  ^36^ | Age 15-65 years  Biopsy-proven idiopathic membranous nephropathy (within last 12 months) with nephrotic syndrome | 103 | Prednisolone 125mg every alternate day for 8 weeks.  Patients >80 kg received 150 mg on alternative days | Placebo for 8 weeks | Serum creatinine (umol/L): Active group 114 ± 42; control group 115 ± 43  Glomerular filtration rate (mL/min/1.73m^2^): Active group 87 ± 30; control group 89 ± 34 | 24 hour Proteinuria (g/24 hour)  Active 10.8 ± 5.9  Control 10.4 ± 5.3 | 72 months | Active group 45 ± 11.6; control group 44 ± 12.1 |
| Cattran 1989  ^37^ | Adult patients Biopsy-proven idiopathic membranous nephropathy  Proteinuria >0.3g/day  Corrected Creatinine clearance of >0.25 mL/sec | 158 | Prednisone: 45 mg/m² in a single dose on alternate days for 6 months. | No specific treatment for 6 months | Serum creatinine (umol/L)  Active 120 ± 10  Control 103 ± 9  Creatinine clearance (mL/sec/1.73m^2^) Active 1.3 ± 0.08 Control 1.5 ± 0.08 | 24 hour Proteinuria (g/24 hour)  Active 6.9 ± 0.8  Control 5.2 ± 0.9 | 48 months | Median, range  Active 46, 18-77  Control 45, 16-83 |
| Donadio 1974  ^38^ | Adults  Biopsy-proven idiopathic membranous nephropathy with nephrotic syndrome | 22 | Oral cyclophosphamide  1.5 to 2.5mg/kg/d (mean: 1.8) for 1 year. | No treatment | Serum creatinine (mg/dL)  Active 1.2, 0.8-1.9  Control 1.1, 0.8-2.2  Glomerular filtration rate (mL/min/1.73m^2^)  Active 75, 44-117; Control 80.6, 33-112 | 24 hour Proteinuria (g/24 hour)  Active 7.8, 2-16.6  Control 7.6, 2-12.1 | 24 months | Active (males: 41, 25-74; females: 48.5, 40-59)  Control (males: 47.6, 34-69; females: 41, 26-65) |
| Murphy 1992  ^39^ | Age >18 years  Biopsy-proven idiopathic membranous nephropathy with proteinuria ≥0.5g/d | 40 | Cyclophosphamide maximum dosage of l.5 mg/kg/d for 6 months.  Dipyridamole and sodium warfarin therapy were continued for 2 years | Symptomatic treatment only | Serum creatinine (µmol/L)  Active 110 (50-280)  Control 90 (50-200) | 24 hour Proteinuria (g/24 hour)  Active 5.0 (0.9-13)  Control 3.9 (0.5-12) | 24 months | Active 47 (26-66)  Control 40 (18-65) |
| Ahmed 1994  ^40^ | Biopsy-proven idiopathic membranous nephropathy with nephrotic syndrome  Serum creatinine < 1.7 mg/dL | 20 | Months 1, 3, 5: intravenous methylprednisolone 1 g/day for 3 consecutive days then oral prednisone 0.5 mg/kg/day for 27 days  Months 2, 4, 6: Oral chlorambucil 0.2 mg/kg/d for 1 month | Oral prednisone 1-5 mg/kg/d for 8 weeks and then in tapering dose and finally withdrawal after 8 weeks | Serum creatinine (mg/dL)  Active group 1.35 ± 0.13 Control group 1.22 ± 0.16 | Proteinuria (g/24 hour)  Active 6.11 ± 1.86  Control 7.61 ± 1.99 | 15 months | Active group 32 ± 7; Control group 38 ± 14 |
| Pahari 1993 ^41^ | Biopsy-proven idiopathic membranous nephropathy  Proteinuria> 2g/day | 71 | Oral prednisone 4mg/kg/day for 3 consecutive days, then oral prednisone 0.5mg/kg/day for 27 days then oral cyclophosphamide 2mg/kg/d for 30 days. Ongoing alternating cycles for 1 year | Oral prednisone: 60 mg/day for 12 weeks | Serum creatinine (mg/dL) <2 | Proteinuria >2g/day | 36 months | Active 35 ± 16  Control 32 ± 20 |
| Tiller 1981  ^42^ | Biopsy-proven idiopathic membranous nephropathy and nephrotic syndrome | 54 | Cyclophosphamide l.5 mg/kg/day plus  dipyridamole 100mg four times a day and warfarin sodium therapy for 6 months | Symptomatic treatment | NA | 24 hour Proteinuria (g/24 hour)  Active 5  Control 4.2 | 36 months | NA |
| Kibriya 1994 ^43^ | Adults with biopsy proven membranous nephropathy  Urinary protein excretion >10g/day  Serum albumin <21g/L  Impaired kidney function (serum creatinine >133umol/L and or creatinine clearance <80mL/min/1.73m2)  Focal glomerulosclerosis  Interstitial fibrosis  Tubular atrophy  Arterial hypertension | 36 | Prednisolone 60mg/day for initial 2-4 weeks then 50mg/day for 2 weeks, 40mg/day for 2 weeks, and thereafter 60mg every alternate day for 2 weeks, thereafter according to clinical response  Low dose oral cyclophosphamide 0.75-1mg/kg orally for 30-60days and then the dose was reduced by one-third and was stopped when proteinuria was less than 3.5g/day | Prednisolone 60mg/day for initial 2-4 weeks then 50mg/day for 2 weeks, 40mg/day for 2 weeks, and thereafter 60mg every alternate day for 2 weeks, thereafter according to clinical response  High dose cyclophosphamide 8-10mg/kg intravenously monthly and if no response every 2 weeks. Discontinued if significant improved in proteinuria <5g/day | NA | 24 hour proteinuria  (g/24 hour)  Both groups together 11.3 + 5.36 | 36 months | 46.5 |
| Peng 2016  ^44^ | Age 18-75 years  Biopsy-proven membranous nephropathy  Persistent proteinuria (> 8g/d) after observation for at least 1 month  No previous immunosuppression | 90 | Tacrolimus 0.05 mg/kg/day in two divided doses. Trough levels 4-8 ng/mL for 6 months and then reduced to 2-4 ng/mL in subsequent 3 months. PLUS  Oral prednisone 0.5 mg/kg/day  Mycophenolate mofetil 1.5-2g/day in two doses plus prednisone 1 mg/kg/day for 9 months | Intravenous cyclophosphamide 750 mg/m^2^ once a month for 6 months, then reduced to 750 mg/m^2^ every 3 months. PLUS oral prednisone 1 mg/kg/day | Serum creatinine (umol/L)  Tacrolimus 82.4 + 13.6  Mycophenolate mofetil 78.7 + 13.8  Cyclophosphamide 78.4 + 13.8  Glomerular filtration rate (mL/min/1.73m^2^)  Tacrolimus 87.9 + 16.5  Mycophenolate mofetil 95.8 + 24.9  Cyclophosphamide 97.3 + 3.8 | 24 hour Proteinuria (g/24 hour)  Tacrolimus 11.7 + 3.2  Mycophenolate mofetil 11.2 + 3.7  Cyclophosphamide 11.9 + 1.5 | 9 months | Tacrolimus 43.9 ± 13.2  Mycophenolate mofetil 39.9 ± 14.3  Cyclophosphamide 40.8 ± 13.3 |
| Ramachandran 2016  ^45^ | Age 18-60 years  Biopsy proven idiopathic membranous nephropathy  Persistent Nephrotic syndrome  Serum creatinine <2.5mg/dL | 70 | Tacrolimus 0.1 mg/kg/d in two divided doses for one year. Aim trough levels 5-10ng/mL in first 6 months, 4-8 ng/mL in the next 6 months. PLUS  Oral prednisone 0.5 mg/kg/d for 6 months and was then tapered and stopped | Months 1, 3, 5: Intravenous methylprednisolone 1g/day for 3 consecutive days then oral prednisolone 0.5 mg/kg/day for 27 days  Months 2, 4, 6: oral cyclophosphamide 2 mg/kg/d for one month | Serum creatinine (mg/dL):  Active 0.90 ± 0.27  Control 0.91 ± 0.26  Glomerular filtration rate (mL/min/1.73m^2^):  Active 96.72 ± 27.13  Control 89.04 ± 27.63 | 24 hour Proteinuria (g/24 hour)  Active 6.76 ± 3.59  Control 5.44 ± 2.66 | 24 months | Active 38.66 ± 1.91  Control 40.80 ± 10.64 |
| Liu 2015  ^46^ | Biopsy-proven idiopathic membranous nephropathy  Nephrotic syndrome | 72 | Leflunomide plus steroids  Leflunomide plus cyclophosphamide plus steroids | Cyclophosphamide plus steroids | NA | NA | NA | NA |
| Omrani 2017  ^47^ | Age 15-70 years  Biopsy-proven idiopathic membranous nephropathy | 68 | Tacrolimus 0.05mg/kg/day plus low dose prednisone for 6 months | Cyclosporine 3-6mg/kg/day plus low dose prednisone for 6 months | Serum creatinine (mg/dL)  Active 1.3 + 0.7  Control 1.3 + 0.8  Creatinine clearance (%)  Active 77.2 + 21.8 Control 73.2 + 33.3 | 24 hour Proteinuria (g/24 hour)  Active group 3.9 + 1.1  Control group 3.9 + 1.5 | 6 months | Active 39.4 + 13.5  Control 36.2 + 14.3 |
| Yuan 2013  ^48^ | Age 16-69 years  Biopsy-proven idiopathic membranous nephropathy I-III  Nephrotic syndrome | 42 | Tacrolimus 0.05-0.08 mg/kg/d in two divided doses aiming trough level 5-8 ng/mL plus oral prednisone 30mg/d for 8 weeks and then tapered by 5mg every 4 weeks until a dose of 10mg/day reached for total of 24 months | Tacrolimus 0.05-0.08 mg/kg/d in two divided doses aiming trough level 5-8 ng/mL plus oral prednisone 30mg/d for 8 weeks and then tapered by 5mg every 4 weeks until a dose of 10mg/day reached for 6 months | Serum creatinine (umol/L)  Active 76.7 + 14.9  Control 72.6 + 15.8  Creatinine clearance (mL/min/1.73m^2^)  Active 94.4 + 16.4 Control 95.2 + 17.8 | 24 hour Proteinuria (g/24 hour)  Active 8.15 + 2.62  Control 9.07 + 2.73 | 24 months | Active 47.2 + 15.8  Control 55.4 + 13.7 |
| Sun 2014  ^49^ | Biopsy-proven idiopathic membranous nephropathy | 60 | Tacrolimus 0.05mg/kg/day in two divided dose aiming trough levels of 4-8 ng/mL for 3 months and slowly tapering PLUS Wuzhi capsules starting at 1 capsule three times a day PLUS  Prednisone 30mg/d initially for 8 weeks and then reduced by 5mg every 4 weeks until dose 10mg/day | Tacrolimus 0.05mg/kg/day in two divided dose aiming trough level 4-8 ng/mL for 3 months and slowly tapering plus prednisone 30mg/day initially for 8 weeks and then reduced by 5mg every 4 weeks until dose 10mg/day | Serum creatinine (umol/L)  Active 86.90 + 19.80  Control 88.25 + 22.57 | 24 hour Proteinuria (g/24 hour)  Active 11.32 + 3.68  Control 11.74 + 2.98 | 6 months | Active 40.15 + 10.05  Control 39.37 + 11.73 |
| Dede 2008  ^50^ | Age 20 - 56 yrs  Biopsy-proven idiopathic membranous nephropathy  Nephrotic range proteinuria | 33 | Pulse intravenous cyclophosphamide 500 mg/m^2^ per month for 6 months  Plus oral prednisone 0.5mg/kg/d for 8 weeks and then slowly tapered but for total of 1 year | Oral cyclophosphamide 1.5-2mg/kg/d for 6 months plus oral prednisone 0.5mg/kg/d for 8 weeks and then slowly tapered but for total of 1 year | Serum creatinine (mg/dL)  Active 0.9 + 0.2  Control 0.9 + 0.5 | Proteinuria (g/10mmol Creatinine)  Active 7.5 + 5.3  Control 10.3 + 6 | 12 months | Active 39 + 16  Control 34 + 9 |
| Gopal 2003  ^51^ | Idiopathic membranous nephropathy | 60 | Months 1, 3, 5: intravenous methylprednisolone 1 g/day for 3 consecutive days then oral prednisone 0.5 mg/kg/day for 27 days  Months 2, 4, 6: Oral chlorambucil 0.2 mg/kg/d for 1 month  Oral steroid | Angiotensin converting enzyme inhibitors | Serum creatinine (mg/dL) Chlorambucil 1.46 + 0.03  Steroid 1.4 + 0.01  Angiotensin converting enzyme inhibitor 1.36 + 0.01 | 24 hour Proteinuria (g/24 hour) Chlorambucil 3.6 + 0.7  Steroids 3.8 + 0.6  Angiotensin converting enzyme inhibitor 3.2 + 0.7 | 24 months | 32 + 12 |
| Hasegawa 2017 ^52^ | Biopsy prven membranous nephropathy  Age >65 yrs  Nephrotic syndrome | 36 | Prednisolone 30mg (tapering) plus mizoribine 150mg | Prednisolone 30mg (tapering) | Serum creatinine (mg/dl)  Active 0.93 + 0.21  Control 0.91 + 0.22 | Urine protein to creatinine ratio (g/g creatinine)  Active 6.79 + 3.51  Control 5.90 + 3.47 | 12 months | 73.3 |
| He 2013 ^53^ | Age 16-70 years  Biopsy proven idiopathic membranous nephropathy  Proteinuria >3.5g/day  Serum albumin <25g/L  Serum creatinine <133umol/L with creatinine clearance >60mL/min/1.73m^2^ | 56 | Tacrolimus 1mg/day for 1 week and then 1mg one day and 2mg the other. The dosage of 2mg was divided into 2 equal doses at 12 hours apart. Target trough level 2-4ng/mL. Treatment for 12 months  Oral prednisolone 1mg/kg/day (maximum 60mg/day) for 4 weeks. Then tapered gradually by 5mg every 2 weeks and then slowly over 12 months | Cyclophosphamide intravenously 750mg/m2 every 4 weeks for 24 weeks.  Oral prednisolone 1mg/kg/day (maximum 60mg/day) for 4 weeks. Then tapered gradually by 5mg every 2 weeks and then slowly over 12 months | Serum creatinine (umol/L)  Active 81.56+27.22  Control 82.45+26.36  eGFR (mL/min/1.73m^2^)  Active 77.35+28.38  Control 76.16+25.24 | 24 hour proteinuria (g/24 hour)  Active 6.76+2.33  Control 6.38+2.19 | 12 months | Active 45.4  Control 47.2 |
| Hofstra 2010 ^54^ | Age 18-75 years  Biopsy proven membranous nephropathy  Proteinuria >3.5g/day  Serum albumin< 35g/l  Normal kidney function  Urinary beta2-microglobulin >0.5ug/min  Urinary IgG >125mg/24hour | 29 | Early treatment immediately after randomization  Cyclophosphamide 1.5mg/kg/day for 12 months plus methylprednisolone 1mg on days 1, 2, 3, 60, 61, 61, 120, 121 and 122and oral prednisone 0.5mg/kg/day for 6 months and subsequently tapered by decreasing the dose by 5mg/week | Late treatment started when kidney function deteriorated  Cyclophosphamide 1.5mg/kg/day for 12 months plus methylprednisolone 1mg on days 1, 2, 3, 60, 61, 61, 120, 121 and 122and oral prednisone 0.5mg/kg/day for 6 months and subsequently tapered by decreasing the dose by 5mg/week | Serum creatinine (umol/L)  Active 94 (68-122)  Control 101 (75-126)  eGFR (mL/min/1.73m^2^)  Active 81 + 17  Control 76 + 13 | 24 hour Proteinuria (g/10mmol)  Active 9.6 (5.9-14.4)  Control 12 (5.6-17.2) | 72 months | Active 48  Control 49 |
| Saito 2014  ^55^ | Age 16 – 75 years  Biopsy-proven idiopathic membranous nephropathy  Proteinuria >3.5g/day and serum albumin <3.0g/dL  Prednisolone treatment for >4 weeks did not decrease proteinuria <1g/day  No prior treatment with cyclosporine microemulsion preconcentrate | 50 | Oral cyclosporine microemulsion preconcentrate 2-3mg/kg once a day plus prednisone for 48 weeks. Prednisone initially at 40mg/day and tapered gradually to <10mg/day by 48 weeks | Oral Cyclosporine microemulsion preconcentrate 1.5mg/kg twice a day plus prednisone for 48 weeks. Prednisone initially at 40mg/day and tapered gradually to <10mg/day by 48 weeks | Serum creatinine (mg/dL)  Active 0.8 (0.5 – 1.2)  Control 0.8 (0.6 – 1.6) | 24 hour Proteinuria (g/24 hour)  Active 3.5 (1.8-10)  Control 3.8 (1.0 – 6.5) | 48 weeks | Active 56  Control 57 |
| Dahan 2017  ^56^ | Age ≥18 years  idiopathic membranous nephropathy  Proteinuria >3.5g/day or urinary protein to creatinine ratio of >3500mg/g and serum albumin <30g/L for ≥6 months  Estimated glomerular filtration rate >30 mL/min/1.73m^2^ | 75 | Rituximab 375 mg/m^2^ on days 1 and 8. At the end of 6 months further immunosuppression was at the discretion of the treating nephrologist  plus non immunosuppressive antiproteinuric treatment | Nonimmunosuppressive antiproteinuric treatment for 6 months | Serum creatinine (umol/L)  Active 98.1 (73.4-122.9)  Control 91.1 (74.3-122) | Protein to creatinine ratio (mg/g)  Active 7680 (4584.3 – 10399)  Control 7195.1 (5363.1 – 8965.1) | 6 months and then post study follow up of 24 months | Active 53 (42-63)  Control 58.5 (43-64) |
| Fervenza 2019  ^57^ | Age 18-80 years  Biopsy-proven idiopathic membranous nephropathy  Proteinuria >5g/day and <50% decline in proteinuria despite at least 3 months of renin angiotensin aldosterone blockade  Stable 24 hour Creatinine clearance ≥ 40mL/min/1.73 m^2^ | 130 | Intravenous Rituximab 1000mg days 1 and 15. At 6 months further course of rituximab was administered if proteinuria was reduced from baseline ≥25% but there was no complete remission | Oral cyclosporine A 3.5mg/kg/day in two equally divided doses for 6 months aiming for trough levels 125-175 ng/mL. At 6 months. If complete remission was achieved cyclosporine A was tapered and discontinued over a 2 month period. If proteinuria was reduced ≥25%, cyclosporine A was continued for an additional 6 months. If proteinuria was reduced by <25% from baseline, patient was considered as treatment failure and cyclosporine A ceased at 6 months | Serum creatinine (mg/dL)  Active 1.3 + 0.4  Control 1.3 + 0.4 | 24 hour Proteinuria (g/24 hour)  Active 8.9 (6.8-12.3)  Control 8.9 (6.7-12.9) | 24 months | Active 51.9 + 12.6  Control 52.2 + 12.4 |
| Chen 2013  ^58^ | Age 18- 75 years  Biopsy-proven idiopathic membranous nephropathy  Proteinuria > 3.5g/day  Estimated glomerular filtration rate >30mL/min/1.73m^2^ | 190 | Shenqi particle  Shenqi particle 9.6g three times per day for 48 weeks | Intravenous cyclophosphamide 0.8-1g/m^2^ BSA once every month for 6 months and then once every 3 months for 6 months with a total dose of 9 -12 g/m^2^plus oral prednisolone 1mg/kg/d for 12 weeks and then tapered by 10mg every 2 weeks to 30mg/d, then by 5mg every 2 weeks to 20mg/d total of 48 weeks | Serum creatinine (umol/L)  Active 82 + 41.5  Control 77.1 + 23.6  Estimated glomerular filtration rate (mL/min/1.73 m^2^)  Active 84 + 27.4  Control 83.8 + 24.9 | 24 hour Proteinuria (g/24 hour)  Active 5.34 + 2.74  Control 5.33 + 2.47 | 48 weeks | Active 49 + 14  Control 53 + 12 |
| Dussol 2006  ^59^ | Idiopathic membranous nephropathy with nephrotic syndrome | 31 | Mycophenolate  mofetil 2g/day for 1 year plus symptomatic treatment | Symptomatic treatment (Angiotensin convertase enzyme inhibitor, diuretics, statins, low salt diet) | Serum creatinine (umol/L)  Both groups together 95 + 33 | Protein/creatinine ratio both groups together 0.74 | 12 months | 51 + 15 |
| Li 2017  ^60^ | Age 18-60 years  Biopsy-proven idiopathic membranous nephropathy with nephrotic syndrome  Persistent proteinuria >8g/day  Serum creatinine of <133umol/L | 31 | Tacrolimus 0.05-0.1 mg/kg/d in two divided doses aiming trough levels 5-10ng/mL for 6 months plus oral prednisone 0.5 mg/kg/d. Dose tapered slowly by 5mg per month down to a dosage of 10mg/d for the remainder of study | Cyclosporine A 3-5 mg/kg/day in two divided doses aiming for a trough level of 100-200ng/mL for 6 months plus oral prednisone 0.5mg/kg/d  Dose was tapered slowly by 5mg per month down to a dosage of 10mg/d for the remainder of study | Serum creatinine (umol/L) Active group 71.8 + 17.4  Control group 73.3 + 16.5 | 24 hour Proteinuria (g/24 hour)  Active 9.5 + 1.9  Control 9.7 + 2.5 | 6 months | Active 39.4 + 8.8  Control 42.8 + 8.1 |
| Ponticelli 1983  ^61^ | Idiopathic membranous nephropathy with nephrotic syndrome  Proteinuria >3.5g/day | 49 | Months 1, 3, 5: Intravenous methylprednisolone 1g daily for 3 consecutive days then oral prednisone 0.5mg/kg for 27 days  Months 2, 4, 6: oral chlorambucil 0.2mg/kg/d for one month. | Supportive therapy | Serum creatinine (mg/100mL)  Active 1.06 + 0.24  Control 1.10 + 0.31 | NA | 3 years | NA |
| Ponticelli 2006 ^62^ | Idiopathic membranous nephropathy with nephrotic syndrome  Proteinuria >3.5g/day  Serum albumin <2.5g/dL | 32 | Tetracosactide 1mg initially every other week to 2 injections every week for 1 year | Months 1, 3, 5: Intravenous methylprednisolone 1g daily for 3 consecutive days then oral prednisone 0.5mg/kg for 27 days  Months 2, 4, 6: oral chlorambucil 0.2mg/kg/d or oral cyclophosphamide 2.5mg/kg/day for one month. | Serum creatinine (mg/dL)  Active 1+0.36  Control 0.9+0.17 | 24 hour proteinuria (g/24 hour)  Active 6.7+2.8  Control 5.5+2.0 | 12 months | Active 48  Control 51.4 |
| Li 2015  ^63^ | Age >65 years  Biopsy-proven idiopathic membranous nephropathy Stage I and II  Proteinuria > 4g/day | 27 | Low dose cyclosporine A f 2mg/kg/day for 6 months. Aiming trough levels 80-120 ng/mL. | Low dose of cyclosporine A plus low dose steroids  cyclosporine A 2mg/kg/day aiming for trough levels 80-120ng/mL plus oral methylprednisone 0.4mg/kg/day, gradually decreased after 8-12 weeks. Total of 6 months | Serum creatinine (umol/L)  Active 98.8 + 15.1  Control 91.6 + 20.9  Estimated glomerular filtration rate (mL/min/1.73 m^2^)  Active 69.6 + 10.3  Control 70.9 + 11.9 | 24 hour Proteinuria (g/24 hour)  Active 7.2 + 3.4  Control 7.5 + 3.8 | 12 months | Active 75.1 + 8.2  Control 74.8 + 7.9 |
| Jurubita 2012  ^64^ | Idiopathic membranous nephropathy  Proteinuria of >8g/day | 18 | Cyclosporine A 2mg/kg/day plus  mycophenolate mofetil 1g/day plus oral prednisone 0.15mg/kg/d for 12 months | Cyclosporine A 5mg/kg/day plus oral prednisone 0.15mg/kg/day for 12 months | NA | 24 hour Proteinuria (g/24 hour)  Active 10.4 (8.4 to 14.9)  Control 10.26 (8 to 14.1) | 12 months | NA |
| Yao 2001 ^65^ | Biopsy proven membranous nephropathy | 30 | Cyclosporin A 5mg/kg/day tailed off over the first three months and maintained at 2mg/kg/day for 12 months | Captopril 37.5mg/day | NA | NA | 12 | NA |
| Saito 2017 ^66^ | Age 16-75 years  Biopsy proven membranous nephropathy  Urine protein >3.5g/day  Serum albumin <3g/dL  Total cholesterol >250mg/dL | 51 | Prednisolone 40mg/day and tapered gradually to <10mg/day at 48 weeks PLUS  Mizorbine 150mg once a day | Prednisolone 40mg/day and tapered gradually to <10mg/day at 48 weeks PLUS  Mizorbine 50mg three times a day | Serum creatinine (mg/dL)  Active 0.8 (0.5-1.3)  Control 0.9 (0.6–0 .4) | 24 hour proteinuria (g/24 hour)  Active 3.7 (1.0-7.5)  Control 3.3 (1.3-7.1) | 24 months | Active 60  Control 60 |
| Wang 2016 ^67^ | Age <55 years  Biopsy proven membranous nephropathy  24 hour urine protein excretion >3.5grams  Normal kidney function  No previous immunsuppression | 55 | Mizoribine 150mg orally once a day for 1 year and methylprednisolone 48mg for 12 weeks, then dose reduced to 4mg per week. At dose of 24mg dosing schedule was reduced to 4mg per two weeks. At 8mg the dose level wan maintained long term | Cyclophosphamide 1gm intravenously first dose and then 0.6mg every two weeks. When total 6grsm of cyclophosphamide was dosed, the dosing schedule was reduced to 1gm every two months plus asteroids as per active arm | Serum creatinine (umol/L)  Active 65.88 + 15.89  Control 68.93 + 29.42 | 24 hour proteinuria (g/24hour)  Active 8.25 + 3.37  Control 7.09 + 2.71 | 12 months | Active 42.5  Control 48.6 |
| Nikolopoulou 2019 ^68^ | Age 18-80 years  Biopsy proven idiopathic membranous nephropathy  Proteinuria >100mg/mmol on urinary protein to creatinine (uPCR)with serum albumin<35g/l or uPCR>300mg/mmol with normal serum albumin | 40 | Tacrolimus 2mg twice a day titrated to achieve blood level of 5-12ng/mL  plus mycophenolate mofetil 500mg twice a day titrated to achieve blood mycophenolic acid level of 1.5-3.0mg/L for 12 months | Tacrolimus 2mg twice a day titrated to achieve blood level of 5-12ng/mL for 12 months | Serum creatinine (mg/dL)  Active 0.8 (0.5–1.2)  Control 0.8 (0.5-1.4)  Estimate glomerular filtration rate (mLs/min/1.73m^2^)  Active 121 (63-201)  Control 109 (44-142) | Urinary protein to creatinine ratio (mg/mmol)  Active 756 (123-1784)  Control 704 (203-2159) | 150 weeks | NA |
| Fernandez-Juarez 2020 ^69^ | Age>18 years  Biopsy proven idiopathic membranous nephropathy  Estimated glomerular filtration rate >45mL/min/1.73m^2^  Proteinuria >4g/24hour with serum albumin<3.5g/dL | 86 | Sequential tacrolimus and rituximab.  Tacrolimus 0.05mg/kg/day, dose adjusted to blood level of 5-7ng/mL for 6 months. After 6 months tacrolimus slowly tapered and complete withdrawal at the end of month 9. Single dose of Rituximab 1gm intravenous administered at month 6 before the onset of tacrolimus dose reduction. | Months 1, 3, 5: Intravenous methylprednisolone 1g/day for 3 consecutive days then oral prednisolone 0.5 mg/kg/day for 27 days  Months 2, 4, 6: oral cyclophosphamide 2 mg/kg/d for one month | NA | NA | 24 months | NA |
| Scolari 2020 ^70^ | Biopsy proven idiopathic membranous nephropathy  Proteinuria >3.5g/24 hour  Estimated glomerular filtration rate >30mL/min/1.73m^2^ | 74 | Rituximab 1gm intravenous on days 1 and 15 | Months 1, 3, 5: Intravenous methylprednisolone 1g/day for 3 consecutive days then oral prednisolone 0.5 mg/kg/day for 27 days  Months 2, 4, 6: oral cyclophosphamide 2 mg/kg/d for one month | NA | NA | 24 months | NA |

## Supplementary Table 3: Definition of complete and partial remission in each trial

| Study | Complete remission | Partial remission |
| --- | --- | --- |
| Ahmed 1994^40^ | NA | NA |
| Austin 1996^31^ | NA | NA |
| Badri 2013^17^ | NA | NA |
| BMJ 1971^14^ | NA | NA |
| Branten 1998^30^ | Reduction in urinary protein-creatinine index to <0.2g/10mmol creatinine | Urinary protein-creatinine index of between 0.2 to 2grams/10mmol creatinine |
| Cameron 1990^36^ | NA | NA |
| Cattran 1989^37^ | Proteinuria of less than 0.3grams/day | Reduction in proteinuria by more than 50% from initial value and being <3g/day |
| Cattran 1995^33^ | Proteinuria of less than 0.3grams/day | Reduction in proteinuria by more than 50% from initial value and being <3g/day |
| Cattran 2001 ^18^ | Proteinuria <0.3grams/day plus stable kidney function | Proteinuria <3.5grams/day and 50% lower than baseline proteinuria plus stable kidney function |
| Chan 2007^28^ | Proteinuria <0.3grams/day with quiescent urinary sediment, normal serum albumin and stable serum creatinine within 15% of baseline value | Proteinuria reduction by at least 50% but remaining above 0.3grams/day, serum albumin not below 30g/L and stable creatinine |
| Chen 2010^23^ | Proteinuria <0.3grams/day plus stable kidney function | Proteinuria <3.5grams/day and 50% lower than baseline proteinuria plus stable kidney function |
| Chen 2013^58^ | Proteinuria <0.3grams/day | Proteinuria between 0.3 to 3.5grams/day and a 50% reduction from its peak value |
| Coggins 1979^16^ | Proteinuria of <0.2grams/day | Proteinuria of 0.21 to 2grams/day |
| Dahan 2017^56^ | Proteinuria of <0.5grams/day or < urine protein to creatinine ratio of 500mg/g creatinine | Proteinuria <3.5grams/day or urine protein to creatinine ratio of <3500mg/g creatinine with >50% reduction compared to baseline |
| Dede 2008^50^ | Proteinuria <0.2g/10mmol creatinine with normal or stable kidney function | Proteinuria 0.2-2.0g/10mmol creatinine with normal or stable kidney function |
| Donadio 1974^38^ | NA | NA |
| Dussol 2006^59^ | Proteinuria <0.3grams/day | Proteinuria <3grams/day with stable kidney function |
| Dussol 2008^25^ | Proteinuria of less than 0.3grams/day and normal kidney function (eGFR>60mL/min/1.73m^2^) | Proteinuria of 0.3 to 3grams/day and stable kidney function (eGFR>60mL/min/1.73m^2^) |
| Falk 1992^35^ | NA | NA |
| Fervenza 2019^57^ | Proteinuria <0.3grams/day and a serum albumin of at least 3.5g/dL | Reduction in proteinuria of at least 50% from baseline plus final proteinuria between 0.3 to 3.5grams/day regardless of creatinine clearance or the serum albumin |
| Fernandez-Juarez 2020^69^ | Proteinuria <0.3grams/day plus stable kidney function (eGFR>45mL/min/1.73m^2^) | Reduction in proteinuria >50% from baseline and value <3.5grams/day plus stable kidney function (eGFR>45mL/min/1.73m^2^) |
| Gopal 2003^51^ | NA | NA |
| Hasegawa 2017^52^ | Urine protein to creatinine ratio of <0.3 | Type 1 partial remission - urine protein to creatinine ratio of 0.3 to <1.0 and type 2 partial remission - urine protein to creatinine ratio 1.0 to <3.5 |
| He 2013^53^ | NA | NA |
| Hofstra 2010^54^ | Urine protein to creatinine index <0.2g/10mmol creatinine with improved or stabilized kidney function | Urine protein to creatinine index <2.0grams/10mmol creatinine with improved or stabilized kidney function |
| Howman 2013^20^ | NA | NA |
| Jha 2007 ^27^ | Proteinuria <0.2grams/day on at least three occasions | Proteinuria between 0.2 to 2grams/day or <50% of baseline along with normal serum creatinine |
| Jurubita 2012^64^ | Urine protein to creatinine ratio of <0.2gram/gram | Urine protein to creatinine <2gram/gram |
| Kibriya 1994^43^ | Proteinuria <0.2grams/day | Proteinuria between >0.2grams/day and <2.0grams/day |
| Kosmadakis 2010^22^ | Proteinuria <0.3grams/day | Proteinuria <3.5grams/day plus a reduction from its peak value |
| Li 2015^63^ | Proteinuria <0.3grams/day, serum albumin >35g/L and stable kidney function (15% increase from baseline) | Decrease in urine protein by more than 50% of baseline, proteinuria <3.5grams/day, stable kidney function |
| Li 2017^60^ | Proteinuria of less than 0.3grams/day and stable kidney function | Proteinuria of 0.3 to 3.5grams/day that had declined to <50% of the baseline value with a serum albumin concentration of at least 30g/L and a stable kidney function |
| Liu 2015^46^ | NA | NA |
| Murphy 1992^39^ | Proteinuria of <0.2grams/day | Fall in proteinuria to less than 3grams/day with rise in serum albumin to >30g/l |
| Naumovic 2011^21^ | Proteinuria of <0.2grams/day | Proteinuria of 0.2 to 3.5grams/day or a reduction of initial proteinuria by >50% |
| Nayagam 2008^24^ | Urine protein to creatinine ratio of <0.3 | Urine protein to creatinine ratio of 0.3 to 2 or <50% proteinuria of baseline with stable kidney function |
| Nikolopoulou 2019^68^ | Urine protein to creatinine ratio of <30mg/mmol | Decrease in urine protein to creatinine ratio by more than 50% but remained above 30mg/mmol and less than 300mg/mmol |
| Omrani 2017^47^ | NA | NA |
| Pahari 1993 ^41^ | Proteinuria <0.2grams/day | Proteinuria 0.2 to 2grams/day |
| Peng 2016^44^ | Proteinuria <0.3grams/day, normal serum albumin and stable kidney function | Proteinuria 0.3 to 3.5grams/day that had declined to <50% of the baseline with serum albumin of at least 30g/L and a stable kidney function |
| Ponticelli 1983^61^ | Proteinuria <0.2grams/day with normal kidney function | Proteinuria 0.2 to 2grams/day with normal kidney function |
| Ponticelli 1995^32^ | Proteinuria <0.2grams/day with a normal plasma creatinine concentrations | Proteinuria between 0.21 and 2grams/day with a normal plasma creatinine concentrations |
| Ponticelli 1998^29^ | Proteinuria <0.2grams/day for at least 1 week with a normal plasma creatinine | Proteinuria between 0.21 and 2grams/day for at least 1 week with a normal plasma creatinine |
| Ponticelli 2006^62^ | Proteinuria of less than 0.3grams/day with normal serum creatinine concentration | Decrease of proteinuria of at least 50% from baseline leading to proteinuria with less than 3.5grams/day with a normal serum creatinine concentration |
| Praga 2007 ^26^ | Proteinuria <0.5grams/day plus stable kidney function (eGFR>50mL/min/1.73m^2^) | Proteinuria <3.5grams/day and 50% lower than baseline proteinuria plus stable kidney function |
| Ramachandran 2016^45^ | Proteinuria <0.5grams/day with normal serum albumin and creatinine | Proteinuria 0.5 to <2grams/day or <50% of baseline with normal serum albumin and serum creatinine |
| Reichert 1994^34^ | Proteinuria less than 0.3grams/day | Proteinuria between 0.3 to 3grams/day |
| Saito 2014^55^ | Proteinuria of <0.3grams/day | Urine proteinuria of 0.3 to 3.5grams/day |
| Saito 2017^66^ | Proteinuria of <0.3grams/day | Urine proteinuria of 0.3 to 3.5grams/day |
| Scolari 2020^70^ | Proteinuria <0.3grams/day | Proteinuria at least 50% lower than the baseline and <3.5grams/day |
| Silverberg 1976 ^15^ | NA | NA |
| Sun 2014^49^ | Proteinuria <0.3grams/day with normal serum creatinine and albumin | Decrease in proteinuria by more than 50% but complete remission not achieved plus stable kidney function and serum albumin >30g/L |
| Tiller 1981^42^ | NA | NA |
| Wang 2016^67^ | Proteinuria of <0.5grams/day | Reduction in proteinuria by >50% |
| Xu 2013 ^19^ | Proteinuria 0.5grams/day with normal kidney function | Proteinuria of 0.5 to 3.5grams/day that had declined to <50% of the baseline value with stable kidney function |
| Yao 2001^65^ | NA | NA |
| Yuan 2013^48^ | Proteinuria <0.4grams/day with serum albumin >30g/l | Proteinuria between 0.4 to 2.9grams/day and decline in proteinuria over 50% to the basal level plus serum albumin >30g/l |

## NA: Not available

## Supplementary Table 4: Risk of bias assessment of included studies

| **Trial name** | **Sequence generation** | **Description of sequence generation** | **Allocation concealment** | **Description of allocation concealment** | **Blinding of participants and investigators** | **Blinding of participants and investigators** | **Blinding of outcome assessment** | **Blinding of outcome assessment** | **Incomplete outcome data** | **Selective reporting** |
| --- | --- | --- | --- | --- | --- | --- | --- | --- | --- | --- |
| BMJ 1971 | Unclear | Not reported | Unclear | Not reported | Unclear | Not reported | Unclear | Not reported | Unclear | Unclear |
| Canmedaj 1976 | Unclear | Not reported | Unclear | Not reported | Unclear | Not reported | Unclear | Not reported | Unclear | Unclear |
| Ahmed 1994 | Unclear | Not reported | Unclear | Not reported | Unclear | Not reported | Unclear | Not reported | Unclear | Unclear |
| Austin 1996 | Unclear | Not reported | Unclear | Not reported | Unclear | Not reported | Unclear | Not reported | Unclear | Unclear |
| Badri 2013 | Unclear | Not reported | Unclear | Not reported | Unclear | Not reported | Unclear | Not reported | Unclear | Unclear |
| Branten 1998 | Unclear | Not reported | Unclear | Not reported | Unclear | Not reported | Unclear | Not reported | Unclear | Unclear |
| Cameron 1990 | Low risk | Not reported | Low risk | Central | Low risk | Yes | Unclear | Not reported | Low risk | Low risk |
| Cattran 2001 | Low risk | Random number table | Low risk | Central | Low risk | Participant blinded | Unclear | Not reported | Low risk | Low risk |
| Cattran 1989 | Unclear | Not reported | Unclear | Not reported | High risk | Not blinded | Unclear | Not reported | Unclear | Unclear |
| Cattran 1995 | Unclear | Not reported | Unclear | Not reported | Unclear | Not reported | Unclear | Not reported | Unclear | Unclear |
| Chan 2007 | Unclear | Not reported | Unclear | Not reported | Unclear | Not reported | Unclear | Not reported | Unclear | Unclear |
| Chen 2010 | Low risk | Table of random numbers | Low risk | Opaque closed envelopes | Unclear | Not reported | Unclear | Not reported | Unclear | Unclear |
| Chen 2013 | Unclear | Not reported | Unclear | Not reported | Unclear | Not reported | Unclear | Not reported | Unclear | Unclear |
| Coggins 1979 | Unclear | Not reported | Unclear | Not reported | Unclear | Not reported | Unclear | Not reported | Unclear | Unclear |
| Dahan 2017 | Unclear | Not reported | Unclear | Not reported | Unclear | Not reported | Unclear | Not reported | Low risk | Low risk |
| Dede 2008 | Unclear | Not reported | Unclear | Not reported | Unclear | Not reported | Unclear | Not reported | Unclear | Unclear |
| Donadio 1974 | Low risk | Table of random numbers | Low risk | Table concealed by pathologists | High risk | No | Unclear | Not reported | Unclear | Unclear |
| Dussol 2008 | Low risk | Central | Unclear | Not reported | Unclear | Not reported | Unclear | Not reported | Unclear | Unclear |
| Dussol 2006 | Unclear | Not reported | Unclear | Not reported | Unclear | Not reported | Unclear | Not reported | Unclear | Unclear |
| Falk 1992 | Unclear | Not reported | Unclear | Not reported | Unclear | Not reported | Unclear | Not reported | Unclear | Unclear |
| Gopal 2003 | Unclear | Not reported | Unclear | Not reported | Unclear | Not reported | Unclear | Not reported | Unclear | Unclear |
| Hasegawa 2017 | Unclear | Not reported | Unclear | Not reported | Unclear | Not reported | Unclear | Not reported | Unclear | Unclear |
| He 2013 | Low risk | Random number table | Unclear | Not reported | Unclear | Not reported | Unclear | Not reported | Unclear | Unclear |
| Hofstra 2010 | Unclear | Not reported | Unclear | Not reported | Unclear | Not reported | Unclear | Not reported | Unclear | Unclear |
| Howman 2013 | Low risk | Random number table | Low risk | Central | High risk | Not blinded | Unclear | Not reported | Unclear | Unclear |
| Jha 2007 | Low risk | Random number table | Unclear | Not reported | Unclear | Not reported | Unclear | Not reported | Unclear | Unclear |
| Jurubita 2012 | Unclear | Not reported | Unclear | Not reported | Unclear | Not reported | Unclear | Not reported | Unclear | Unclear |
| Kosmadakis 2010 | Unclear | Not reported | Unclear | Not reported | Unclear | Not reported | Unclear | Not reported | Low risk | Low risk |
| Li 2015 | Unclear | Not reported | Unclear | Not reported | Unclear | Not reported | Unclear | Not reported | Unclear | Unclear |
| Li 2017 | Low risk | Table of random number | Unclear | Not reported | Unclear | Not reported | Unclear | Not reported | Unclear | Unclear |
| Liu 2015 | Unclear | Not reported | Unclear | Not reported | Unclear | Not reported | Unclear | Not reported | Unclear | Unclear |
| Murphy 1992 | Unclear | Not reported | Unclear | Not reported | Unclear | Not reported | Unclear | Not reported | Unclear | Unclear |
| Naumovic 2011 | Unclear | Not reported | Unclear | Not reported | Unclear | Not reported | Unclear | Not reported | Unclear | Unclear |
| Omrani 2017 | Unclear | Not reported | Unclear | Not reported | Unclear | Not reported | Unclear | Not reported | Unclear | Unclear |
| Pahari 1993 | Unclear | Not reported | Unclear | Not reported | Unclear | Not reported | Unclear | Not reported | Unclear | Unclear |
| Peng 2016 | Unclear | Not reported | Unclear | Not reported | Unclear | Not reported | Unclear | Not reported | Unclear | Unclear |
| Ponticelli 1998 | Low risk | Random order | Low risk | Central | Unclear | Not reported | Unclear | Not reported | Unclear | Unclear |
| Ponticelli 2006 | Low risk | Random Number | Low risk | Central | Unclear | Not reported | Unclear | Not reported | Unclear | Unclear |
| Ponticelli 1983 | Unclear | Not reported | Unclear | Not reported | High risk | Not masked to treatment | Unclear | Not reported | Low risk | Low risk |
| Ponticelli 1995 | Low risk | Random number tables | Low risk | Sealed envelope | High risk | Unblinded | Unclear | Not reported | Unclear | Unclear |
| Praga 2007 | Low risk | Random number tables | Low risk | Sequentially numbered opaque envelopes | Unclear | Not reported | Unclear | Not reported | Unclear | Unclear |
| Ramachandran 2017 | Unclear | Not reported | Unclear | Not reported | Unclear | Not reported | Unclear | Not reported | Unclear | Unclear |
| Reichert 1994 | Unclear | Not reported | Unclear | Not reported | High risk | Not masked to treatment | Unclear | Not reported | Low risk | Low risk |
| Saito 2017 | Unclear | Not reported | Unclear | Not reported | Unclear | Not reported | Unclear | Not reported | Unclear | Unclear |
| Saito 2014 | Unclear | Not reported | Unclear | Not reported | Unclear | Not reported | Unclear | Not reported | Low risk | Low risk |
| Senthil Nayagam 2008 | Unclear | Not reported | Unclear | Not reported | Unclear | Not reported | Unclear | Not reported | Unclear | Unclear |
| Sun 2014 | Unclear | Not reported | Unclear | Not reported | Unclear | Not reported | Unclear | Not reported | Unclear | Unclear |
| Tiller 1981 | Unclear | Not reported | Unclear | Not reported | Unclear | Not reported | Unclear | Not reported | Unclear | Unclear |
| Wang 2016 | Unclear | Not reported | Unclear | Not reported | Unclear | Not reported | Unclear | Not reported | Low risk | Low risk |
| Xu 2013 | Unclear | Not reported | Unclear | Not reported | Unclear | Not masked to treatment | Unclear | Not reported | Low risk | Low risk |
| Yao 2001 | Unclear | Not reported | Unclear | Not reported | Unclear | Not reported | Unclear | Not reported | Unclear | Unclear |
| Yuan 2013 | Low risk | Preprinted randomization table | Unclear | Not reported | Unclear | Not reported | Unclear | Not reported | Low risk | Low risk |
| Fervenza 2019 | Low risk | Random number | Low risk | Web-based | High risk | Unblinded | Unclear | Not reported | Low risk | Low risk |
| Nikolopoulou 2019 | Low risk | Ransom number | Low risk | Sealed envelope | Unclear | Not reported | Unclear | Not reported | Low risk | Unclear |
| Fernandez-Juarez 2020 | Unclear | Not reported | Unclear | Not reported | Unclear | Not reported | Unclear | Not reported | Unclear | Unclear |
| Delbarba 2020 | Unclear | Not reported | Unclear | Not reported | Unclear | Not reported | Unclear | Not reported | Unclear | Unclear |

## Supplementary Table 5: Assessment of agreement between direct and indirect source of evidence using side-splitting approach for all outcomes

| **Intervention** | **Comparison** | **Direct** | | **Indirect** | | **Difference** | | **P value** |
| --- | --- | --- | --- | --- | --- | --- | --- | --- |
|  |  | **Coefficient** | **SE** | **Coefficient** | **SE** | **Coefficient** | **SE** |  |
| **Complete remission** | |  |  |  |  |  |  |  |
| Cyclophosphamide | Chlorambucil | -0.79 | 0.61 | 0.72 | 0.67 | -1.51 | 0.90 | 0.09 |
| Cyclophosphamide | Calcineurin inhibitor | -0.002 | 0.47 | -0.78 | 0.73 | 0.78 | 0.88 | 0.37 |
| Cyclophosphamide | Rituximab | 0.29 | 0.77 | 2.32 | 1.01 | -2.04 | 1.27 | 0.11 |
| Cyclophosphamide | Steroid | . | . | . | . | . | . | . |
| Cyclophosphamide | Mycophenolate mofetil | 0.42 | 0.66 | -1.54 | 0.64 | 1.96 | 0.92 | 0.03 |
| Cyclophosphamide | Mizoribine | . | . | . | . | . | . | . |
| Cyclophosphamide | ACTH | . | . | . | . | . | . | . |
| Cyclophosphamide | Non immunosuppressive therapy | -0.68 | 0.64 | -1.43 | 0.50 | 0.74 | 0.81 | 0.36 |
| Cyclophosphamide | Leflunomide | . | . | . | . | . | . | . |
| Cyclophosphamide | Calcineurin inhibitor plus rituximab | . | . | . | . | . | . | . |
| Chlorambucil | Mycophenolate mofetil | -0.29 | 1.15 | -0.53 | 0.67 | 0.24 | 1.33 | 0.86 |
| Chlorambucil | Steroid | -0.97 | 0.73 | 0.04 | 1.29 | -1.00 | 1.50 | 0.50 |
| Chlorambucil | Non immunosuppressive therapy | -1.96 | 0.67 | -0.26 | 0.61 | -1.71 | 0.91 | 0.06 |
| Calcineurin inhibitor | Rituximab | 4.30 | 1.53 | 0.57 | 0.71 | 3.73 | 1.68 | 0.03 |
| Calcineurin inhibitor | Steroid | -0.53 | 1.40 | -0.63 | 0.80 | 0.10 | 1.61 | 0.95 |
| Calcineurin inhibitor | Mycophenolate mofetil | -1.61 | 0.69 | 0.45 | 0.58 | -2.06 | 0.90 | 0.02 |
| Calcineurin inhibitor | Azathioprine | -0.41 | 1.05 | 0.51 | 132.80 | -0.91 | 132.80 | 1.00 |
| Calcineurin inhibitor | Non immunosuppressive therapy | -0.89 | 0.71 | -0.92 | 0.57 | 0.03 | 0.91 | 0.97 |
| Rituximab | Non immunosuppressive therapy | -2.16 | 1.26 | -2.25 | 0.86 | 0.10 | 1.53 | 0.95 |
| Steroid | Non immunosuppressive therapy | -0.67 | 0.92 | 0.08 | 0.94 | -0.75 | 1.32 | 0.57 |
| Steroid | Placebo | -0.74 | 0.70 | 1.57 | 163.73 | -2.31 | 163.74 | 0.99 |
| Mycophenolate mofetil | Non immunosuppressive therapy | -0.18 | 0.74 | -0.88 | 0.69 | 0.70 | 1.01 | 0.49 |
| **Complete remission at 6 months** | |  |  |  |  |  |  |  |
| Cyclophosphamide | Calcineurin inhibitor | 0.59 | 0.47 | 1.55 | 2.07 | -0.96 | 2.12 | 0.65 |
| Cyclophosphamide | Rituximab | 0.43 | 1.12 | -0.53 | 1.81 | 0.96 | 2.13 | 0.65 |
| Cyclophosphamide | Mycophenolate mofetil | 0.31 | 0.73 | -2.80 | 1.23 | 3.10 | 1.46 | 0.03 |
| Cyclophosphamide | Mizoribine | . | . | . | . | . | . | . |
| Cyclophosphamide | Calcineurin inhibitor plus rituximab | . | . | . | . | . | . | . |
| Calcineurin inhibitor | Rituximab | -1.11 | 1.75 | -0.15 | 1.21 | -0.96 | 2.13 | 0.65 |
| Calcineurin inhibitor | Steroid | -0.53 | 1.37 | -0.53 | 113.41 | 0.007 | 113.42 | 1.00 |
| Calcineurin inhibitor | Mycophenolate mofetil | -1.46 | 0.62 | 1.65 | 1.40 | -3.10 | 1.46 | 0.03 |
| Calcineurin inhibitor | Azathioprine | 0.92 | 1.77 | -1.52 | 233.14 | 2.45 | 233.15 | 0.99 |
| Calcineurin inhibitor | Non immunosuppressive therapy | -0.29 | 1.11 | -1.28 | 193.36 | 0.99 | 193.36 | 1.00 |
| Steroid | Placebo | -0.66 | 0.91 | -0.32 | 250.20 | -0.34 | 250.20 | 1.00 |
| **Partial remission at 6 months** | |  |  |  |  |  |  |  |
| Cyclophosphamide | Calcineurin inhibitor | -0.33 | 0.49 | -0.06 | 1.13 | -0.27 | 1.23 | 0.83 |
| Cyclophosphamide | Rituximab | -0.65 | 0.83 | -0.92 | 0.91 | 0.27 | 1.23 | 0.83 |
| Cyclophosphamide | Mycophenolate mofetil | -0.21 | 0.77 | 1.28 | 1.59 | -1.48 | 1.77 | 0.40 |
| Cyclophosphamide | Mizobrine | . | . | . | . | . | . | . |
| Cyclophosphamide | Calcineurin inhibitor plus rituximab | . | . | . | . | . | . | . |
| Calcineurin inhibitor | Rituximab | -0.59 | 0.77 | -0.32 | 0.96 | -0.27 | 1.23 | 0.83 |
| Calcineurin inhibitor | Steroid | -2.31 | 0.82 | 0.38 | 81.74 | -2.69 | 81.75 | 0.97 |
| Calcineurin inhibitor | Mycophenolate mofetil | 0.64 | 0.76 | -0.84 | 1.60 | 1.48 | 1.77 | 0.40 |
| Calcineurin inhibitor | Azathioprine | 1.20 | 1.02 | 0.55 | 132.81 | 0.65 | 132.82 | 1.00 |
| Calcineurin inhibitor | Non immunosuppressive therapy | -2.89 | 1.20 | -0.52 | 139.02 | -2.37 | 139.02 | 0.99 |
| Steroid | Placebo | -0.44 | 0.92 | 5.20 | 213.65 | -5.64 | 213.65 | 0.98 |
| **Partial remission** | |  |  |  |  |  |  |  |
| Cyclophosphamide | Chlorambucil | -0.28 | 0.55 | -0.63 | 0.62 | 0.35 | 0.82 | 0.67 |
| Cyclophosphamide | Calcineurin inhibitor | -0.49 | 0.36 | 0.57 | 0.53 | -1.06 | 0.64 | 0.10 |
| Cyclophosphamide | Rituximab | -0.12 | 0.74 | -0.01 | 0.57 | -0.10 | 0.93 | 0.91 |
| Cyclophosphamide | Mycophenolate mofetil | -0.58 | 0.59 | 0.25 | 0.60 | -0.83 | 0.83 | 0.32 |
| Cyclophosphamide | Mizobrine | . | . | . | . | . | . | . |
| Cyclophosphamide | ACTH | . | . | . | . | . | . | . |
| Cyclophosphamide | Non immunosuppressive therapy | 0.02 | 0.32 | -1.12 | 0.35 | 1.15 | 0.47 | 0.02 |
| Cyclophosphamide | Calcineurin inhibitor plus rituximab | . | . | . | . | . | . | . |
| Chlorambucil | Mycophenolate mofetil | 0.13 | 1.06 | 0.28 | 0.58 | -0.14 | 1.21 | 0.91 |
| Chlorambucil | Steroids | 0.17 | 0.73 | -1.66 | 0.96 | 1.82 | 1.23 | 0.14 |
| Chlorambucil | Non immunosuppressive therapy | -0.44 | 0.62 | -0.32 | 0.58 | -0.12 | 0.85 | 0.89 |
| Calcineurin inhibitor | Rituximab | 0.27 | 0.67 | -0.09 | 0.64 | 0.36 | 0.93 | 0.70 |
| Calcineurin inhibitor | Steroid | -1.92 | 0.90 | 0.05 | 0.75 | -1.97 | 1.17 | 0.09 |
| Calcineurin inhibitor | Mycophenolate mofetil | 0.65 | 0.71 | -0.53 | 0.62 | 1.18 | 0.94 | 0.21 |
| Calcineurin inhibitor | Azathioprine | 0.88 | 0.97 | 0.17 | 126.61 | 0.71 | 126.62 | 1.00 |
| Calcineurin inhibitor | Non immunosuppressive therapy | -1.82 | 0.76 | -0.21 | 0.41 | -1.61 | 0.86 | 0.06 |
| Rituximab | Non immunosuppressive therapy | -0.61 | 0.71 | -0.89 | 0.65 | 0.28 | 0.96 | 0.77 |
| Steroid | Non immunosuppressive therapy | -0.37 | 0.95 | 0.47 | 0.78 | -0.85 | 1.24 | 0.49 |
| Steroid | Placebo | -0.44 | 0.92 | 1.81 | 213.49 | -2.26 | 213.49 | 1.00 |
| Mycopheonolate  mofetil | Non immunosuppressive therapy | -0.74 | 0.65 | -0.51 | 0.59 | -0.23 | 0.87 | 0.79 |
| **Kidney Failure** | |  |  |  |  |  |  |  |
| Cyclophosphamide | Chlorambucil | -1.86 | 1.25 | -1.44 | 1.02 | -0.42 | 1.62 | 0.80 |
| Cyclophosphamide | Calcineurin inhibitor | -0.02 | 1.43 | 0.50 | 0.89 | -0.52 | 1.68 | 0.76 |
| Cyclophosphamide | Rituximab | . | . | . | . | . | . | . |
| Cyclophosphamide | Steroid | 0.24 | 0.70 | 2.08 | 1.66 | -1.85 | 1.81 | 0.31 |
| Cyclophosphamide | Mycophenolate mofetil | -0.03 | 2.02 | 0.39 | 1.58 | -0.42 | 2.56 | 0.87 |
| Cyclophosphamide | ACTH | . | . | . | . | . | . | . |
| Cyclophosphamide | Non immunosuppressive therapy | 1.46 | 1.14 | -0.52 | 0.93 | 1.98 | 1.47 | 0.18 |
| Cyclophosphamide | Calcineurin plus rituximab | . | . | . | . | . | . | . |
| Chlorambucil | Calcineurin inhibitor | 2.28 | 1.04 | 1.53 | 1.25 | 0.74 | 1.74 | 0.67 |
| Chlorambucil | Non immunosuppressive therapy | 1.66 | 0.67 | 3.22 | 1.60 | -1.56 | 1.72 | 0.37 |
| Calcineurin inhibitor | Mycophenolate mofetil | 1.08e-11 | 2.02 | -0.19 | 1.50 | 0.19 | 2.51 | 0.94 |
| Calcineurin inhibitor | Non immunosuppressive therapy | -0.50 | 0.69 | 1.17 | 1.20 | -1.67 | 1.39 | 0.23 |
| Calcineurin inhibitor | Placebo | 2.08 | 1.27 | 0.23 | 1.28 | 1.85 | 1.81 | 0.31 |
| Steroid | Placebo | 0.76 | 0.64 | 2.61 | 1.69 | -1.85 | 1.81 | 0.31 |
| Mycophenolate mofetil | Non immunosuppressive therapy | 3.50e-12 | 1.45 | 0.12 | 1.83 | -0.12 | 2.34 | 0.96 |
| **Doubling of creatinine** | |  |  |  |  |  |  |  |
| Cyclophosphamide | Chlorambucil | . | . | . | . | . | . | . |
| Cyclophosphamide | Calcineurin inhibitor | -0.02 | 1.53 | 0.05 | 1.49 | -0.06 | 2.13 | 0.98 |
| Cyclophosphamide | Steroid | 2.06 | 1.59 | -0.41 | 1.60 | 2.47 | 2.26 | 0.27 |
| Cyclophosphamide | Mycophenolate mofetil | -0.03 | 2.15 | 0.09 | 4.43 | -0.13 | 4.92 | 0.98 |
| Cyclophosphamide | Non immunosuppressive therapy | 0.73 | 1.35 | 2.92 | 1.72 | -2.18 | 2.18 | 0.32 |
| Calcineurin inhibitor | Steroid | 0.21 | 1.12 | 2.68 | 1.96 | -2.47 | 2.26 | 0.27 |
| Calcineurin inhibitor | Mycophenolate mofetil | -8.23e-12 | 2.15 | -0.13 | 4.43 | 0.13 | 4.92 | 0.98 |
| Calcineurin inhibitor | Non immunosuppressive therapy | 2.27 | 1.24 | 0.09 | 1.80 | 2.18 | 2.19 | 0.32 |
| Steroid | Placebo | 0.26 | 0.82 | -1.67 | 124.83 | 1.93 | 124.83 | 0.99 |
| **Proteinuria** | |  |  |  |  |  |  |  |
| Cyclophosphamide | Chlorambucil | 0.42 | 4.09 | -0.36 | 9.63 | 0.78 | 10.46 | 0.94 |
| Cyclophosphamide | Calcineurin inhibitor | -0.78 | 1.33 | -1.21 | 5.91 | 0.43 | 6.02 | 0.94 |
| Cyclophosphamide | Steroid | -2.10 | 18.89 | 0.50 | 2.10 | -2.60 | 19.00 | 0.89 |
| Cyclophosphamide | Mycophenolate mofetil | 0.10 | 3.58 | -0.13 | 6.38 | 0.23 | 7.47 | 0.98 |
| Cyclophosphamide | Mizobrine | . | . | . | . | . | . | . |
| Cyclophosphamide | Non immunosuppressive therapy | 0.48 | 1.92 | -0.94 | 4.55 | 1.42 | 5.18 | 0.78 |
| Chlorambucil | Steroid mofetil | 0.80 | 9.39 | 0.02 | 4.62 | 0.78 | 10.46 | 0.94 |
| Calcineurin inhibitor | Mycophenolate mofetil | 0.80 | 3.29 | 1.03 | 6.84 | -0.23 | 7.47 | 0.98 |
| Calcineurin inhibitor | Azathioprine | -1.00 | 10.44 | -0.41 | 11.56 | -0.59 | 15.58 | 0.97 |
| Calcineurin inhibitor | Placebo | 4.70 | 14.40 | 1.19 | 2.19 | 3.51 | 14.56 | 0.81 |
| Calcineurin inhibitor | Non immunosuppressive therapy | 0.38 | 2.75 | 1.73 | 2.96 | -1.35 | 4.31 | 0.75 |
| Steroid | Placebo | 3.46e-11 | 0.65 | 0.006 | 9.22 | -0.006 | 9.24 | 1.00 |
| Azathioprine | Non immunosuppressive therapy | 4.70 | 16.79 | 0.94 | 8.77 | 3.76 | 18.94 | 0.84 |
| Azathioprine | Placebo | -1.10 | 15.52 | 3.06 | 9.04 | -4.16 | 17.96 | 0.82 |
| **Serious adverse events** | |  |  |  |  |  |  |  |
| Cyclophosphamide | Chlorambucil | 1.08 | 1.16 | 0.28 | 0.90 | 0.80 | 1.47 | 0.59 |
| Cyclophosphamide | Calcineurin inhibitor | -0.56 | 0.70 | -0.17 | 0.84 | -0.39 | 1.09 | 0.72 |
| Cyclophosphamide | Rituximab | 0.40 | 0.96 | -1.33 | 0.81 | 1.73 | 1.26 | 0.17 |
| Cyclophosphamide | Calcineurin inhibitor plus rituximab | . | . | . | . | . | . | . |
| Cyclophosphamide | Mycophenolate mofetil | -0.41 | 0.94 | -1.46 | 0.98 | 1.05 | 1.35 | 0.44 |
| Cyclophosphamide | Mizoribine | . | . | . | . | . | . | . |
| Cyclophosphamide | Steroid | 0.10 | 1.04 | 0.64 | 48.07 | -0.55 | 48.08 | 0.99 |
| Cyclophosphamide | Non immunosuppressive therapy | -2.58 | 0.97 | -0.63 | 0.58 | -1.95 | 1.13 | 0.09 |
| Chlorambucil | Calcineurin inhibitor | -0.50 | 0.96 | -1.54 | 1.06 | 1.04 | 1.43 | 0.47 |
| Chlorambucil | Non immunosuppressive therapy | -1.53 | 0.86 | -2.40 | 1.20 | 0.87 | 1.44 | 0.54 |
| Calcineurin inhibitor | Rituximab | -0.78 | 0.89 | 0.25 | 0.85 | -1.03 | 1.23 | 0.40 |
| Calcineurin inhibitor | Mycophenolate mofetil | -0.40 | 0.99 | -0.62 | 0.96 | 0.22 | 1.38 | 0.87 |
| Calcineurin inhibitor | Non immunosuppressive therapy | -0.54 | 0.72 | -1.25 | 0.80 | 0.71 | 1.07 | 0.51 |
| Rituximab | Non immunosuppressive therapy | -0.24 | 1.08 | -0.88 | 0.91 | 0.63 | 1.41 | 0.65 |
| Mycophenolate mofetil | Non immunosuppressive therapy | -0.02 | 0.84 | -0.75 | 0.97 | 0.73 | 1.26 | 0.56 |
| Steroid | Placebo | -1.28 | 0.67 | -0.19 | 96.17 | -1.09 | 96.17 | 0.99 |
| **Discontinuation** | |  |  |  |  |  |  |  |
| Cyclophosphamide | Chlorambucil | 1.08 | 0.85 | 2.51 | 0.84 | -1.43 | 1.20 | 0.23 |
| Cyclophosphamide | Calcineurin inhibitor | 0.72 | 0.45 | -0.24 | 1.32 | 0.96 | 1.40 | 0.49 |
| Cyclophosphamide | Steroid | 0.80 | 0.55 | -1.36 | 1.36 | 2.16 | 1.47 | 0.14 |
| Cyclophosphamide | Non immunosuppressive therapy | -0.26 | 0.54 | 0.23 | 0.86 | -0.49 | 1.02 | 0.63 |
| Chlorambucil | Mycophenolate mofetil | -1.40 | 1.70 | -1.05 | 1.12 | -0.35 | 2.04 | 0.86 |
| Chlorambucil | Steroid | -3.51 | 1.40 | -0.46 | 0.86 | -3.05 | 1.64 | 0.06 |
| Chlorambucil | Non immunosuppressive therapy | -2.34 | 0.92 | -1.54 | 0.87 | -0.80 | 1.28 | 0.54 |
| Calcineurin inhibitor | Rituximab | -1.86 | 0.80 | -1.89 | 210.89 | 0.03 | 210.89 | 1.00 |
| Calcineurin inhibitor | Azathioprine | -0.25 | 2.05 | -0.89 | 1.45 | 0.64 | 2.51 | 0.80 |
| Calcineurin inhibitor | Non immunosuppressive therapy | 0.09 | 1.45 | -0.90 | 0.64 | 0.98 | 1.59 | 0.54 |
| Steroid | Non immunosuppressive therapy | -2.91e-10 | 2.03 | -0.68 | 0.71 | 0.68 | 2.15 | 0.75 |
| Steroid | Placebo | 0.30 | 0.35 | -0.64 | 2.56 | 0.94 | 2.59 | 0.72 |
| Mycophenolate  Mofetil | Non immunosuppressive therapy | -0.83 | 0.91 | -0.48 | 1.82 | -0.35 | 2.04 | 0.86 |
| Azathioprine | Non immunosuppressive therapy | 0.66 | 1.73 | -0.66 | 1.57 | 1.33 | 2.34 | 0.57 |
| Azathioprine | Placebo | 0.20 | 2.10 | 1.14 | 1.51 | -0.94 | 2.59 | 0.72 |
| **Serious infection** | |  |  |  |  |  |  |  |
| Cyclophosphamide | Chlorambucil | 2.59 | 1.77 | -0.58 | 0.92 | 3.17 | 2.00 | 0.11 |
| Cyclophosphamide | Calcineurin inhibitor | -0.35 | 0.55 | 0.22 | 1.03 | -0.57 | 1.17 | 0.63 |
| Cyclophosphamide | Rituximab | -1.13 | 1.20 | 0.20 | 1.08 | -1.33 | 1.62 | 0.41 |
| Cyclophosphamide | Steroid | -1.79 | 1.87 | 0.37 | 100.63 | -2.16 | 100.65 | 0.98 |
| Cyclophosphamide | Mycophenolate mofetil | -0.18 | 1.24 | -0.41 | 1.12 | 0.59 | 1.67 | 0.72 |
| Cyclophosphamide | Mizobrine | . | . | . | . | . | . | . |
| Cyclophosphamide | Non immunosuppressive therapy | -0.62 | 0.93 | -0.35 | 0.92 | -0.26 | 1.33 | 0.84 |
| Cyclophosphamide | Calcineurin inhibitor plus rituximab | . | . | . | . | . | . | . |
| Chlorambucil | Calcineurin inhibitor | 1.05 | 1.17 | -1.44 | 1.06 | 2.50 | 1.57 | 0.11 |
| Chlorambucil | Mycophenolate mofetil | 0.27 | 1.51 | -0.63 | 1.25 | 0.91 | 1.96 | 0.64 |
| Chlorambucil | Non immunosuppressive therapy | -0.67 | 1.16 | -0.52 | 1.32 | -0.15 | 1.76 | 0.93 |
| Calcineurin inhibitor | Rituximab | 0.13 | 1.15 | -0.46 | 1.15 | 0.59 | 1.63 | 0.72 |
| Calcineurin inhibitor | Mycophenolate mofetil | 0.38 | 1.25 | -0.17 | 1.14 | 0.55 | 1.70 | 0.75 |
| Calcineurin inhibitor | Azathioprine | 1.52 | 1.91 | -0.13 | 194.63 | 1.65 | 194.65 | 0.99 |
| Calcineurin inhibitor | Non immunosuppressive therapy | -0.65 | 1.00 | 0.05 | 0.87 | -0.70 | 1.35 | 0.61 |
| Rituximab | Non immunosuppressive therapy | -1.15 | 1.96 | 0.20 | 1.02 | -1.35 | 2.21 | 0.54 |
| Mycophenolate  mofetil | Non-immunosuppressive therapy | 0.62 | 1.36 | -0.91 | 1.06 | 1.53 | 1.72 | 0.37 |
| **Bone marrow suppression** | |  |  |  |  |  |  |  |
| Cyclophosphamide | Chlorambucil | 2.10 | 1.10 | 1.43 | 0.82 | 0.68 | 1.37 | 0.62 |
| Cyclophosphamide | Calcineurin inhibitor | -1.89 | 0.78 | -1.78 | 1.04 | -0.12 | 1.31 | 0.93 |
| Cyclophosphamide | Rituximab | -2.63 | 1.49 | -2.14 | 2.13 | -0.48 | 2.60 | 0.85 |
| Cyclophosphamide | Mycophenolate mofetil | -1.03 | 1.61 | -1.95 | 1.15 | 0.92 | 1.94 | 0.64 |
| Cyclophosphamide | Mizobrine | . | . | . | . | . | . | . |
| Cyclophosphamide | ACTH | . | . | . | . | . | . | . |
| Cyclophosphamide | Non immunosuppressive therapy | -2.57 | 1.10 | -2.00 | 0.83 | -0.58 | 1.38 | 0.68 |
| Cyclophosphamide | Calcineurin inhibitor plus rituximab | . | . | . | . | . | . | . |
| Chlorambucil | Calcineurin inhibitor | -3.58 | 0.68 | -3.36 | 1.14 | -0.22 | 1.33 | 0.87 |
| Chlorambucil | Mycophenolate mofetil | -3.75 | 1.59 | -3.09 | 1.13 | -0.66 | 1.95 | 0.73 |
| Chlorambucil | Non immunosuppressive therapy | -3.66 | 0.69 | -4.53 | 1.20 | 0.87 | 1.39 | 0.53 |
| Calcineurin inhibitor | Mycophenolate mofetil | -2.76e-11 | 2.02 | 0.27 | 1.09 | -0.27 | 2.29 | 0.91 |
| Calcineurin inhibitor | Azathioprine | 2.61 | 1.55 | 2.03 | 88.31 | 0.58 | 88.33 | 1.00 |
| Calcineurin inhibtor | Non immunosuppressive therapy | -0.51 | 0.72 | 0.10 | 1.25 | -0.61 | 1,45 | 0.68 |
| Rituximab | Non immunosuppressive therapy | -0.03 | 2.01 | 0.46 | 1.64 | -0.48 | 2.60 | 0.85 |
| Mycophenolate  Mofetil | Non immunosuppressive therapy | -0.63 | 1.27 | -0.49 | 1.25 | -0.14 | 1.79 | 0.94 |
| Azathioprine | Placebo | 0.20 | 2.10 | -1.51 | 220.06 | 1.71 | 220.07 | 1.00 |

*Evidence about these contrasts comes from trials that directly compare them. Abbreviations: ACTH = adrenocorticotropic hormone. .=missing values indicated only direct evidence existed and inconsistency between direct and indirect evidence could not be assessed.

## Supplementary Table 6: Summary of confidence estimates in effects of immunosuppression agents on idiopathic membranous nephropathy

| Comparison |  | |  | | Cyclophosphamide vs. | | | | | | | | | |
| --- | --- | --- | --- | --- | --- | --- | --- | --- | --- | --- | --- | --- | --- | --- |
|  | ACTH | Azathioprine | | Chlorambucil | | Calcineurin inhibitor | Leflunomide | Mizoribine | Mycophenolate mofetil | Placebo | Rituximab | Calcineurin inhibitor plus rituximab | Non-immunosuppressive therapy | Steroid |
| **Direct evidence** |  |  | |  | |  |  |  |  |  |  |  |  |  |
| Risk of bias | Not serious |  | |  | | Not serious | Not serious |  | Not serious |  |  |  | Not serious | Not serious |
| Inconsistency | Not detected |  | |  | | Serious | Not detected |  | Not serious |  |  |  | Not serious | Not serious |
| Indirectness | Not serious |  | |  | | Not serious | Not serious |  | Not serious |  |  |  | Not serious | Not serious |
| Publication bias | Not detected |  | |  | | Not detected | Not detected |  | Not detected |  |  |  | Not serious | Not detected |
| Preliminary rating direct | High |  | |  | | Moderate | High |  | High |  |  |  | High | High |
| Contributes as much as indirect | Yes |  | |  | | Yes | No |  | Yes |  |  |  | Yes | Yes |
| Imprecision | Serious |  | |  | | Very serious | Very serious |  | Serious |  |  |  | Not serious | Serious |
| Final direct rating | Moderate |  | |  | | Very low | Low |  | Moderate |  |  |  | High | Moderate |
| **Indirect evidence** |  |  | |  | |  |  |  |  |  |  |  |  |  |
| Common comparator |  |  | |  | | Non-immunosuppressive therapy |  |  | Non-immunosuppressive therapy |  |  |  | Mycophenolate mofetil | Calcineurin inhibitor |
| Tnt1 vs. common comparator rating |  |  | |  | | High |  |  | High |  |  |  | Moderate | Very low |
| Tnt1 vs. common comparator rating |  |  | |  | | Low |  |  | Moderate |  |  |  | Moderate | Moderate |
| Lowest of the two |  |  | |  | | Low |  |  | Moderate |  |  |  | Moderate | Very low |
| Intransitivity |  |  | |  | | Not serious |  |  | Not serious |  |  |  | Not serious | Not serious |
| Preliminary rating indirect |  |  | |  | | Low |  |  | Moderate |  |  |  | Moderate | Very low |
| Imprecision |  |  | |  | | **Serious** |  |  | Serious |  |  |  | Serious | Serious |
| Final indirect rating |  |  | |  | | Low |  |  | Low |  |  |  | Low | Very low |
| **Network evidence** |  |  | |  | |  |  |  |  |  |  |  |  |  |
| Highest between direct and indirect | Moderate | NA | |  | | Low | Low | NA | Moderate | NA | NA | NA | High | Moderate |
| Incoherence | NA | NA | |  | | Not serious | NA | NA | Not serious | NA | NA | NA | Serious | Not serious |
| Imprecision | Serious | Very serious | |  | | Serious | Very serious | Very serious | Serious | Very serious | Serious | Serious | Not serious | Not serious |
| **Final network rating** | **Low** | **Low** | |  | | **Very low** | **Very low** | **Low** | **Low** | **Low** | **Low** | **Low** | **Moderate** | **Moderate** |

Abbreviations: ACTH = adrenocorticotropic hormone

## Supplementary Table 7: Estimated heterogeneity and global inconsistency of outcomes in networks

| **Outcome** | **Number of events** | **Number of trials** | **Number of participants** | **Heterogeneity tau** | **Heterogeneity assessment** | **Chi Square** | **P Value** |
| --- | --- | --- | --- | --- | --- | --- | --- |
| Complete remission | 289 | 28 | 1475 | 0.33 | Low | 25.39 | <0.01 |
| Complete remission at 6 months | 116 | 12 | 915 | 0.29 | Low | 5.04 | 0.08 |
| Partial remission | 166 | 26 | 1354 | 0.20 | Low | 7.48 | 0.76 |
| Partial remission at 6 months | 314 | 11 | 784 | 0.21 | Low | 0.39 | 0.82 |
| Kidney Failure | 23 | 15 | 1014 | 0 | Low | 2.46 | 0.87 |
| Doubling of serum creatinine | 55 | 7 | 431 | 0.32 | NA | 1.21 | 0.75 |
| End of treatment glomerular filtration rate | NA | 4 | 131 | NA | NA | NA | NA |
| End of treatment proteinuria | NA | 14 | 573 | 0.00 | NA | 0.49 | 1.00 |
| Serious adverse events | 178 | 18 | 1318 | 0.57 | Low | 5.92 | 0.66 |
| Discontinuation of treatment | 183 | 18 | 1090 | 0.02 | Low | 4.55 | 0.72 |
| Serious infection | 137 | 20 | 1338 | 2.66 | Low | 3.63 | 0.98 |
| Bone marrow suppression | 59 | 16 | 840 | 0 | Low | 3.24 | 0.95 |

NA – Not applicable

## Supplementary Table 8: Network meta-analysis estimates of comparative treatment association

### Supplementary Table 8.1 Network estimates (odds ratios and 95% CI) of effects of treatment on complete remission at 6 months

| Cyclophosphamide | 1.86 (0.79, 4.41) | 1.19 (0.19, 7.33) | 0.07 (0.00, 1.47) | 0.68 (0.14, 3.32) | 1.05 (0.18, 6.26) | 4.69 (0.13, 14.45) | 1.10 (0.07, 18.44) | 0.57 (0.02, 16.13) | 1.39 (0.13, 14.45) |
| --- | --- | --- | --- | --- | --- | --- | --- | --- | --- |
| 0.54 (0.23, 1.27) | Calcineurin inhibitor | 0.64 (0.09, 4.35) | 0.04 (0.00, 0.89) | 0.37 (0.08, 1.65) | 0.56 (0.08, 4.10) | 2.52 (0.08, 80.94) | 0.59 (0.04, 8.66) | 0.31 (0.01, 7.70) | 0.75 (0.08, 6.59) |
| 0.84 (0.14, 5.20) | 1.57 (0.23, 10.69) | Rituximab | 0.06 (0.00, 2.03) | 0.57 (0.05, 6.13) | 0.88 (0.07, 11.33) | 3.95 (0.07, 208.26) | 0.93 (0.03, 25.12) | 0.48 (0.01, 20.58) | 1.17 (0.06, 21.32) |
| 15.08 (0.68, 333.53) | 28.06 (1.13, 698.25) | 17.90 (0.49, 649.77) | Calcineurin inhibitor plus rituximab | 10.26 (0.32, 332.49) | 15.83 (0.44, 564.78) | 70.69 (0.62, 8006.52) | 16.58 (0.25, 1092.26) | 8.59 (0.09, 818.57) | 20.91 (0.43, 1016.15) |
| 1.47 (0.30, 7.17) | 2.74 (0.61, 12.35) | 1.74 (0.16, 18.65) | 0.10 (0.00, 3.16) | Mycophenolate mofetil | 1.54 (0.14, 16.81) | 6.89 (0.16, 302.88) | 1.62 (0.07, 35.13) | 0.84 (0.02, 29.37) | 2.04 (0.14, 28.86) |
| 0.95 (0.16, 5.68) | 1.77 (0.24, 12.87) | 1.13 (0.09, 14.48) | 0.06 (0.00, 2.25) | 0.65 (0.06, 7.05) | Mizoribine | 4.46 (0.08, 242.86) | 1.05 (0.04, 29.47) | 0.54 (0.01, 24.02) | 1.32 (0.07, 25.15) |
| 0.21 (0.01, 7.62) | 0.40 (0.01, 12.75) | 0.25 (0.00, 13.35) | 0.01 (0.00, 1.60) | 0.15 (0.00, 6.38) | 0.22 (0.00, 12.18) | Azathioprine | 0.23 (0.00, 18.85) | 0.12 (0.00, 13.86) | 0.30 (0.00, 17.80) |
| 0.91 (0.05, 15.25) | 1.69 (0.12, 24.79) | 1.08 (0.04, 29.27) | 0.06 (0.00, 3.97) | 0.62 (0.03, 13.45) | 0.96 (0.03, 26.88) | 4.26 (0.05, 342.76) | Steroids | 0.52 (0.09, 3.09) | 1.26 (0.04, 40.04) |
| 1.76 (0.06, 49.74) | 3.27 (0.13, 82.16) | 2.08 (0.05, 89.39) | 0.12 (0.00, 11.10) | 1.19 (0.03, 41.90) | 1.84 (0.04, 81.65) | 8.23 (0.07, 938.96) | 1.93 (0.32, 11.53) | Placebo | 2.43 (0.05, 119.35) |
| 0.72 (0.07, 7.52) | 1.34 (0.15, 11.87) | 0.86 (0.05, 15.63) | 0.05 (0.00, 2.33) | 0.49 (0.03, 6.95) | 0.76 (0.04, 14.43) | 3.38 (0.06, 203.56) | 0.79 (0.02, 25.19) | 0.41 (0.01, 20.14) | Non immunosuppressive therapy |

The table shows comparisons of complete remission at 6 months among different treatment strategies. Data are odds ratio with 95% confidence interval within bracket. The table should be read from left to right. Risk estimate is for the column-defining treatment compared to the row-defining treatment. An odds ratio <1 indicates the column treatment is associated with lower odds of proteinuria improvement than the row treatment.

### Supplementary Table 8.2. Network estimates (odds ratios and 95% CI) of effects of treatment on partial remission

| Cyclophosphamide | 0.66 (0.30, 1.47) | 0.87 (0.46, 1.63) | 0.96 (0.42, 2.21) | 1.59 (0.43, 5.84) | 0.84 (0.36, 1.97) | 0.52 (0.07, 4.16) | 2.09 (0.28, 15.53) | 0.60 (0.11, 3.16) | 0.40 (0.12, 1.31) | 0.26 (0.03, 2.23) | 0.46 (0.22, 0.94) |
| --- | --- | --- | --- | --- | --- | --- | --- | --- | --- | --- | --- |
| 1.51 (0.68, 3.35) | Chlorambucil | 1.31 (0.51, 3.37) | 1.45 (0.51, 4.15) | 2.40 (0.52, 11.04) | 1.27 (0.48, 3.37) | 0.79 (0.09, 7.28) | 3.15 (0.38, 26.40) | 0.91 (0.14, 5.73) | 0.60 (0.19, 1.92) | 0.39 (0.05, 3.32) | 0.69 (0.19, 1.92) |
| 1.15 (0.61, 2.16) | 0.76 (0.30, 1.96) | Calcineurin inhibitor | 1.10 (0.47, 2.58) | 1.83 (0.43, 7.76) | 0.97 (0.38, 2.45) | 0.60 (0.07, 5.25) | 2.40 (0.36, 16.12) | 0.69 (0.12, 4.08) | 0.46 (0.14, 1.52) | 0.30 (0.03, 2.58) | 0.53 (0.23, 1.21) |
| 1.04 (0.45, 2.40) | 0.69 (0.24, 1.97) | 0.91 (0.39, 2.12) | Rituximab | 1.66 (0.35, 7.76) | 0.88 (0.30, 2.56) | 0.55 (0.06, 5.09) | 2.17 (0.27, 17.49) | 0.62 (0.10, 4.01) | 0.42 (0.11, 1.58) | 0.27 (0.03, 2.53) | 0.48 (0.20, 1.16) |
| 0.63 (0.17, 2.30) | 0.42 (0.09, 1.91) | 0.55 (0.13, 2.31) | 0.60 (0.13, 2.82) | Calcineurin inhibitor plus rituximab | 0.53 (0.11, 2.50) | 0.33 (0.03, 3.79) | 1.31 (0.12, 14.30) | 0.38 (0.05, 3.11) | 0.25 (0.04, 1.46) | 0.16 (0.01, 2.01) | 0.29 (0.07, 1.27) |
| 1.18 (0.51, 2.76) | 0.79 (0.30, 2.08) | 1.03 (0.41, 2.61) | 1.14 (0.39, 3.31) | 1.89 (0.40, 8.89) | Mycophenolate mofetil | 0.62 (0.07, 5.82) | 2.47 (0.30, 20.58) | 0.71 (0.11, 4.59) | 0.47 (0.13, 1.78) | 0.31 (0.03, 2.86) | 0.55 (0.23, 1.27) |
| 1.91 (0.24, 15.16) | 1.27 (0.14, 11.65) | 1.66 (0.19, 14.48) | 1.83 (0.20, 17.11) | 3.04 (0.26, 35.08) | 1.61 (0.17, 15.10) | Mizoribine | 3.99 (0.22, 71.30) | 1.15 (0.08, 16.32) | 0.77 (0.07, 8.33) | 0.49 (0.02, 9.81) | 0.88 (0.10, 7.86) |
| 0.48 (0.06, 3.56) | 0.32 (0.04, 2.66) | 0.42 (0.06, 2.80) | 0.46 (0.06, 3.70) | 0.76 (0.07, 8.33) | 0.40 (0.05, 3.36) | 0.25 (0.01, 4.49) | Azathioprine | 0.29 (0.02, 3.89) | 0.19 (0.02, 1.82) | 0.12 (0.01, 2.20) | 0.22 (0.03, 1.76) |
| 1.67 (0.32, 8.79) | 1.10 (0.17, 6.99) | 1.45 (0.25, 8.58) | 1.60 (0.25, 10.28) | 2.66 (0.32, 21.89) | 1.41 (0.22, 9.08) | 0.87 (0.06, 12.44) | 3.48 (0.26, 47.12) | ACTH | 0.67 (0.09. 5.15) | 0.43 (0.03, 6.55) | 0.77 (0.13, 4.69) |
| 2.50 (0.76, 8.16) | 1.65 (0.52, 5.25) | 2.17 (0.66, 7.16) | 2.40 (0.63, 9.10) | 3.97 (0.68, 23.07) | 2.11 (0.58, 7.89) | 1.31 (0.12, 14.22) | 5.21 (0.55, 49.31) | 1.50 (0.19, 11.54) | Steroid | 0.64 (0.11, 3.91) | 1.15 (0.35, 3.73) |
| 3.88 (0.45, 33.63) | 2.57 (0.30, 21.93) | 3.38 (0.39, 29.39) | 3.73 (0.40, 35.18) | 6.18 (0.50, 76.84) | 3.28 (0.35, 30.67) | 2.03 (0.10, 40.53) | 8.10 (0.45, 144.75) | 2.33 (0.15, 35.54) | 1.56 (0.26, 9.46) | Placebo | 1.79 (0.21, 15.42) |
| 2.17 (1.06, 4.45) | 1.44 (0.65, 3.21) | 1.89 (0.83, 4.33) | 2.09 (0.87, 5.04) | 3.46 (0.79, 15.25) | 1.83 (0.79, 4.27) | 1.14 (0.13, 10.19) | 4.54 (0.57, 36.22) | 1.30 (0.21, 7.97) | 0.87 (0.27, 2.83) | 0.56 (0.06, 4.84) | Non immunosuppressive therapy |

Abbreviations: ACTH = adrenocorticotropic hormone

The table shows comparisons of partial remission among different treatment strategies. Data are odds ratio with 95% confidence interval within bracket. The table should be read from left to right. Risk estimate is for the column-defining treatment compared to the row-defining treatment. An odds ratio <1 indicates the column treatment is associated with lower odds of partial remission than the row treatment.

### Supplementary Table 8.3 Network estimates (odds ratios and 95% CI) of effects of treatment on partial remission at 6 months

| Cyclophosphamide | 0.74 (0.37, 1.48) | 0.45 (0.18, 1.17) | 0.52 (0.15, 1.79) | 1.08 (0.31, 3.80) | 0.81 (0.14, 4.64) | 2.46 (0.30, 20.52) | 0.07 (0.01, 0.43) | 0.05 (0.00, 0.59) | 0.04 (0.00, 0.48) |
| --- | --- | --- | --- | --- | --- | --- | --- | --- | --- |
| 1.35 (0.67, 2.72) | Calcineurin inhibitor | 0.62 (0.25, 1.53) | 0.70 (0.17, 2.91) | 1.46 (0.42, 5.12) | 1.09 (0.17, 7.18) | 3.33 (0.45, 24.68) | 0.10 (0.02, 0.50) | 0.06 (0.01, 0.73) | 0.06 (0.01, 0.59) |
| 2.20 (0.86, 5.64) | 1.62 (0.65, 4.04) | Rituximab | 1.14 (0.24, 5.41) | 2.37 (0.54, 10.43) | 1.78 (0.24, 12.95) | 5.41 (0.60, 48.84) | 0.16 (0.03, 1.03) | 0.10 (0.01, 1.39) | 0.09 (0.01, 1.13) |
| 1.93 (0.56, 6.69) | 1.43 (0.34, 5.93) | 0.88 (0.18, 4.18) | Calcineurin inhibitor plus rituximab | 2.08 (0.35, 12.21) | 1.56 (0.18, 13.33) | 4.76 (0.41, 55.53) | 0.14 (0.02, 1.22) | 0.09 (0.01, 1.53) | 0.08 (0.01, 1.25) |
| 0.93 (0.26, 3.27) | 0.69 (0.20, 2.41) | 0.42 (0.10, 1.86) | 0.48 (0.08, 2.82) | Mycophenolate mofetil | 0.75 (0.09, 6.47) | 2.29 (0.22, 24.30) | 0.07 (0.01, 0.53) | 0.04 (0.00, 0.68) | 0.04 (0.00, 0.55) |
| 1.24 (0.22, 7.12) | 0.91 (0.14, 6.01) | 0.56 (0.08, 4.11) | 0.64 (0.08, 5.47) | 1.33 (0.15, 11.51) | Mizoribine | 3.05 (0.20, 47.61) | 0.09 (0.01, 1.09) | 0.06 (0.00, 1.26) | 0.05 (0.00, 1.04) |
| 0.41 (0.05, 3.38) | 0.30 (0.04, 2.22) | 0.18 (0.02, 1.67) | 0.21 (0.02, 2.45) | 0.44 (0.04, 4.65) | 0.33 (0.02, 5.12) | Azathioprine | 0.03 (0.00, 0.39) | 0.02 (0.00, 0.45) | 0.02 (0.00, 0.37) |
| 13.57 (2.34, 78.59) | 10.03 (2.00, 50.25) | 6.17 (0.97, 39.31) | 7.02 (0.82, 60.37) | 14.61 (1.89, 112.74) | 10.96 (0.92, 130.68) | 33.42 (2.56, 436.71) | Steroids | 0.64 (0.10, 3.94) | 0.56 (0.03, 9.69) |
| 21.11 (1.69, 263.35) | 15.59 (1.38, 176.34) | 9.60 (0.72, 128.09) | 10.92 (0.66, 182.01) | 22.73 (1.48, 348.93) | 17.05 (0.79, 367.43) | 51.98 (2.24, 1206.92) | 1.56 (0.25, 9.53) | Placebo | 0.87 (0.03, 25.52) |
| 24.36 (2.08, 284.87) | 18.00 (1.70, 190.24) | 11.08 (0.88, 138.80) | 12.61 (0.80, 198.23) | 26.23 (1.81, 379.36) | 19.68 (0.96, 402.18) | 59.99 (2.72, 1322.73) | 1.80 (0.10, 31.23) | 1.15 (0.04, 34.00) | Non immunosuppressive therapy |

The table shows comparisons of partial remission at 6 months among different treatment strategies. Data are odds ratio with 95% confidence interval within bracket. The table should be read from left to right. Risk estimate is for the column-defining treatment compared to the row-defining treatment. An odds ratio <1 indicates the column treatment is associated with lower odds of proteinuria improvement than the row treatment.

**Supplementary Table 8.4 Network estimates (odds ratios and 95% CI) of effects of treatment on Kidney Failure**

| Cyclophosphamide | 0.20 (0.04, 0.95) | 1.42 (0.33, 6.25) | 0.21 (0.01, 4.59) | 0.33 (0.01, 8.22) | 1.26 (0.11, 14.32) | 3.19 (0.12, 84.43) | 1.68 (0.47, 5.97) | 4.54 (0.93, 22.15) | 1.32 (0.32, 5.41) |
| --- | --- | --- | --- | --- | --- | --- | --- | --- | --- |
| 4.99 (1.06, 23.56) | Chlorambucil | 7.10 (1.66, 30.47) | 1.06 (0.03, 33.14) | 1.62 (0.05, 58.40) | 6.27 (0.53, 74.03) | 15.92 (0.42, 597.16) | 8.37 (1.28, 54.87) | 22.65 (3.04, 168.92) | 6.57 (1.95, 22.18) |
| 0.70 (0.16, 3.08) | 0.14 (0.03, 0.60) | Calcineurin inhibitor | 0.15 (0.00, 4.51) | 0.23 (0.01, 7.96) | 0.88 (0.08, 9.30) | 2.24 (0.06, 81.44) | 1.18 (0.21, 6.54) | 3.19 (0.54, 18.72) | 0.92 (0.29, 2.97) |
| 4.71 (0.22, 101.87) | 0.94 (0.03, 29.58) | 6.71 (0.22, 203.28) | Rituximab | 1.53 (0.02, 132.37) | 5.92 (0.12, 298.47) | 15.04 (0.17, 1342.59) | 7.90 (0.28, 219.76) | 21.39 (0.67, 679.57) | 6.21 (0.21, 182.78) |
| 3.07 (0.12, 77.50) | 0.62 (0.02, 22.15) | 4.38 (0.13, 152.41) | 0.65 (0.01, 56.26) | Calcineurin inhibitor plus rituximab | 3.86 (0.07, 219.88) | 9.81 (0.10, 974.19) | 5.15 (0.16, 165.34) | 13.95 (0.38, 508.57) | 4.05 (0.12, 137.20) |
| 0.80 (0.07, 9.07) | 0.16 (0.01, 1.89) | 1.13 (0.11, 11.95) | 0.17 (0.00, 8.52) | 0.26 (0.00, 14.76) | Mycophenolate mofetil | 2.54 (0.04, 150.27) | 1.33 (0.09, 18.92) | 3.61 (0.23, 56.07) | 1.05 (0.11, 9.77) |
| 0.31 (0.01, 8.28) | 0.06 (0.00, 2.36) | 0.45 (0.01, 16.21) | 0.07 (0.00, 5.93) | 0.10 (0.00, 10.13) | 0.39 (0.01, 23.27) | ACTH | 0.53 (0.02, 17.61) | 1.42 (0.04, 54.07) | 0.41 (0.01, 14.60) |
| 0.60 (0.17, 2.12) | 0.12 (0.02, 0.78) | 0.85 (0.15, 4.72) | 0.13 (0.00, 3.52) | 0.19 (0.01, 6.23) | 0.75 (0.05, 10.62) | 1.90 (0.06, 63.79) | Steroid | 2.71 (0.84, 8.76) | 0.79 (0.14, 4.51) |
| 0.22 (0.05, 1.07) | 0.04 (0.01, 0.33) | 0.31 (0.05, 1.84) | 0.05 (0.00, 1.48) | 0.07 (0.00, 2.61) | 0.28 (0.02, 4.29) | 0.70 (0.02, 26.73) | 0.37 (0.11, 1.20) | Placebo | 0.29 (0.04, 1.88) |
| 0.76 (0.18, 3.11) | 0.15 (0.05, 0.51) | 1.08 (0.34, 3.47) | 0.16 (0.01, 4.74) | 0.25 (0.01, 8.38) | 0.95 (0.10, 8.89) | 2.42 (0.07, 85.72) | 1.27 (0.22, 7.30) | 3.45 (0.53, 22.36) | Non immunosuppressive therapy |

Abbreviations: ACTH = adrenocorticotropic hormone

The table shows comparisons of kidney failure among different treatment strategies. Data are odds ratio with 95% confidence interval within bracket. The table should be read from left to right. Risk estimate is for the column-defining treatment compared to the row-defining treatment. An odds ratio <1 indicates the column treatment is associated with lower odds of kidney failure than the row treatment.

### Supplementary Table 8.5 Network estimates (odds ratios and 95% CI) of effects of treatment on doubling of serum creatinine

| Cyclophosphamide | 1.59 (0.19, 13.25) | 1.01 (0.14, 7.24) | 0.99 (0.02, 39.43) | 2.35 (0.23, 23.67) | 3.04 (0.17, 52.90) | 4.80 (0.58, 39.84) |
| --- | --- | --- | --- | --- | --- | --- |
| 0.63 (0.08, 5.23) | Chlorambucil | 0.63 (0.03, 11.56) | 0.62 (0.01, 43.77) | 1.48 (0.06, 35.38) | 1.91 (0.05, 71.47) | 3.02 (0.15, 60.02) |
| 0.99 (0.14, 7.14) | 1.58 (0.09, 28.82) | Calcineurin inhibitor | 0.98 (0.02, 39.13) | 2.34 (0.31, 17.59) | 3.02 (0.22, 41.44) | 4.77 (0.62, 36.74) |
| 1.01 (0.03, 40.51) | 1.61 (0.02, 113.81) | 1.02 (0.03, 40.81) | Mycophenolate mofetil | 2.39 (0.04, 136.17) | 3.08 (0.04, 245.60) | 4.87 (0.09, 265.05) |
| 0.42 (0.04, 4.27) | 0.68 (0.03, 16.16) | 0.43 (0.06, 3.22) | 0.42 (0.01, 23.92) | Steroid | 1.29 (0.26, 6.50) | 2.04 (0.14, 29.20) |
| 0.33 (0.02, 5.72) | 0.52 (0.01, 19.54) | 0.33 (0.02, 4.55) | 0.32 (0.00, 25.83) | 0.77 (0.15, 3.89) | Placebo | 1.58 (0.07, 36.92) |
| 0.21 (0.03, 1.73) | 0.33 (0.02, 6.59) | 0.21 (0.03, 1.62) | 0.21 (0.00, 11.19) | 0.49 (0.03, 7.02) | 0.63 (0.03, 14.82) | Non immunosuppressive therapy |

The table shows comparisons of doubling of serum creatinine among different treatment strategies. Data are odds ratio with 95% confidence interval within bracket. The table should be read from left to right. Risk estimate is for the column-defining treatment compared to the row-defining treatment. An odds ratio <1 indicates the column treatment is associated with lower odds of doubling of serum creatinine than the row treatment.

**Supplementary Table 8.6 Network estimates (odds ratios and 95% CI) of effects of treatment on glomerular filtration rate**

| Calcineurin inhibitor | 0.86 (0.16,4.69) | 0.49 (0.21,1.14) | 0.38 (0.09,1.56) | 0.25 (0.05,1.29) |
| --- | --- | --- | --- | --- |
| 1.16 (0.21,6.29) | Steroid | 0.56 (0.13,2.42) | 0.44 (0.07,2.78) | 0.29 (0.18,0.46) |
| 2.06 (0.88,4.84) | 1.78 (0.41,7.66) | Azathioprine | 0.78 (0.25,2.41) | 0.52 (0.13,2.08) |
| 2.63 (0.64,10.80) | 2.27 (0.36,14.35) | 1.28 (0.42,3.93) | Non immunosuppressive therapy | 0.67 (0.11,3.96) |
| 3.96 (0.78,20.15) | 3.42 (2.15,5.43) | 1.92 (0.48,7.68) | 1.50 (0.25,8.95) | Placebo |

The table shows comparisons of glomerular filtration rate among different treatment strategies. Data are odds ratio with 95% confidence interval within bracket. The table should be read from left to right. Risk estimate is for the column-defining treatment compared to the row-defining treatment. An odds ratio <1 indicates the column treatment is associated with lower odds of decline in glomerular filtration rate than the row treatment.

**Supplementary Table 8.7** **Network estimates (odds ratios and 95% CI) of effects of treatment on end of treatment proteinuria**

| Cyclophosphamide | 1.35 (0.00, 2166.55) | 0.45 (0.03, 5.84) | 1.04 (0.00, 413.95) | 0.97 (0.02, 43.40) | 0.22 (0.00, 919997.50) | 1.60 (0.03, 96.03) | 1.60 (0.03, 80.21) | 1.24 (0.05, 32.81) |
| --- | --- | --- | --- | --- | --- | --- | --- | --- |
| 0.74 (0.00, 1188.72) | Chlorambucil | 0.33 (0.00, 792.90) | 0.77 (0.00, 10147.05) | 0.72 (0.00, 2897.62) | 0.16 (0.00, 3.46e+06) | 1.18 (0.00, 3970.14) | 1.18 (0.00, 3742.39) | 0.92 (0.00, 2394.51) |
| 2.22 (0.17, 28.73) | 3.00 (0.00, 7114.15) | Calcineurin inhibitor | 2.32 (0.01, 836.11) | 2.15 (0.02, 210.55) | 0.48 (0.00, 1.89e+06) | 3.55 (0.04, 292.31) | 3.55 (0.05, 246.88) | 2.75 (0.07, 107.33) |
| 0.96 (0.00, 379.62) | 1.29 (0.00, 16960.18) | 0.43 (0.00, 155.76) | Mycophenolate mofetil | 0.93 (0.00, 1112.42) | 0.21 (0.00, 2.33e+06) | 1.53 (0.00, 1841.32) | 1.53 (0.00, 1660.77) | 1.19 (0.00, 919.98) |
| 1.03 (0.02, 46.09) | 1.39 (0.00, 5607.76) | 0.46 (0.00, 45.41) | 1.08 (0.00, 1288.03) | Mizoribine | 0.22 (0.00, 1.51e+06) | 1.65 (0.01, 439.84) | 1.65 (0.01, 386.02) | 1.28 (0.01, 192.93) |
| 4.63 (0.00, 1.98e+07) | 6.26 (0.00, 1.35e+08) | 2.09 (0.00, 8.25e+06) | 4.84 (0.00, 5.45e+07) | 4.50 (0.00, 3.06e+07) | Azathioprine | 7.41 (0.00, 3.46e+07) | 7.41 (0.00, 3.29e+07) | 5.74 (0.00, 2.37e+07) |
| 0.63 (0.01, 37.55) | 0.84 (0.00, 2829.79) | 0.28 (0.00, 23.22) | 0.65 (0.00, 785.21) | 0.61 (0.00, 161.99) | 0.13 (0.00, 630064.59) | Steroid | 1.00 (0.28, 3.54) | 0.78 (0.06, 10.24) |
| 0.63 (0.01, 31.37) | 0.84 (0.00, 2667.29) | 0.28 (0.00, 19.61) | 0.65 (0.00, 708.17) | 0.61 (0.00, 142.16) | 0.13 (0.00, 599682.91) | 1.00 (0.28, 3.54) | Placebo | 0.78 (0.08, 7.44) |
| 0.81 (0.03, 21.36) | 1.09 (0.00, 2841.38) | 0.36 (0.01, 14.19) | 0.84 (0.00, 653.13) | 0.78 (0.01, 118.30) | 0.17 (0.00, 717596.42) | 1.29 (0.10, 17.06) | 1.29 (0.13, 12.38) | Non immunosuppressive therapy |

The table shows comparisons of proteinuria (grams per day) among different treatment strategies. Data are odds ratio with 95% confidence interval within bracket. The table should be read from left to right. Risk estimate is for the column-defining treatment compared to the row-defining treatment. An odds ratio <1 indicates the column treatment is associated with lower levels of proteinuria than the row treatment.

**Supplementary Table 8.8 Network estimates (odds ratios and 95% CI) of effects of discontinuation of treatment**

| Cyclophosphamide | 6.05 (1.86, 19.70) | 1.86 (0.81, 4.27) | 0.29 (0.05, 1.71) | 1.91 (0.33, 11.08) | 0.94 (0.10, 9.15) | 1.63 (0.57, 4.69) | 2.16 (0.61, 7.64) | 0.89 (0.37, 2.10) |
| --- | --- | --- | --- | --- | --- | --- | --- | --- |
| 0.17 (0.05, 0.54) | Chlorambucil | 0.31 (0.05, 1.97) | 0.05 (0.01, 0.40) | 0.31 (0.05, 1.97) | 0.16 (0.01, 1.87) | 0.27 (0.06, 1.20) | 0.36 (0.07, 1.86) | 0.15 (0.04, 0.49) |
| 0.54 (0.23, 1.23) | 3.25 (0.78, 13.50) | Calcineurin inhibitor | 0.16 (0.03, 0.75) | 1.02 (0.15, 6.94) | 0.51 (0.05, 5.13) | 0.88 (0.24, 3.27) | 1.16 (0.27, 5.09) | 0.48 (0.15, 1.48) |
| 3.45 (0.58, 20.39) | 20.88 (2.51, 173.76) | 6.42 (1.33, 30.84) | Rituximab | 6.58 (0.55, 78.13) | 3.26 (0.20, 53.32) | 5.63 (0.73, 43.65) | 7.45 (0.86, 64.34) | 3.06 (0.44, 21.23) |
| 0.52 (0.09, 3.05) | 3.18 (0.51, 19.83) | 0.98 (0.14, 6.61) | 0.15 (0.01, 1.81) | Mycophenolate mofetil | 0.50 (0.03, 7.84) | 0.86 (0.11, 6.43) | 1.13 (0.13, 9.55) | 0.47 (0.09, 2.29) |
| 1.06 (0.11, 10.26) | 6.41 (0.53, 76.93) | 1.97 (0.20, 19.87) | 0.31 (0.02, 5.02) | 2.02 (0.13, 31.92) | Azathioprine | 1.73 (0.16, 18.31) | 2.29 (0.21, 25.31) | 0.94 (0.10, 9.18) |
| 0.61 (0.21, 1.76) | 3.71 (0.83, 16.49) | 1.14 (0.31, 4.24) | 0.18 (0.02, 1.38) | 1.17 (0.16, 8.76) | 0.58 (0.05, 6.13) | Steroid | 1.32 (0.67, 2.59) | 0.54 (0.14, 2.04) |
| 0.46 (0.13, 1.64) | 2.80 (0.54, 14.62) | 0.86 (0.20, 3.77) | 0.13 (0.02, 1.16) | 0.88 (0.10, 7.43) | 0.44 (0.04, 4.84) | 0.76 (0.39, 1.48) | Placebo | 0.41 (0.09, 1.82) |
| 1.13 (0.48, 2.67) | 6.82 (2.03, 22.94) | 2.10 (0.67, 6.52) | 0.33 (0.05, 2.27) | 2.15 (0.44, 10.57) | 1.06 (0.11, 10.41) | 1.84 (0.49, 6.90) | 2.44 (0.55, 10.81) | Non immunosuppressive therapy |

The table shows comparisons of discontinuation of therapy among different treatment strategies. Data are odds ratio with 95% confidence interval within bracket. The table should be read from left to right. Risk estimate is for the column-defining treatment compared to the row-defining treatment. An odds ratio <1 indicates the column treatment is associated with lower odds of discontinuation of therapy than the row treatment.

**Supplementary Table 8.9 Network estimates (odds ratios and 95% CI) of effects of treatment on serious infection**

| Cyclophosphamide | 13.24 (0.18, 990.45) | 0.99 (0.02, 46.12) | 0.29 (0.00, 39.90) | 43.77 (0.13, 14803.36) | 0.83 (0.14, 4.81) | 2.94 (0.02, 446.55) | 2.33 (0.00, 3480.91) | 0.32 (0.01, 9.50) | 0.57 (0.02, 15.53) | 14.53 (0.02, 11560.92) |
| --- | --- | --- | --- | --- | --- | --- | --- | --- | --- | --- |
| 0.08 (0.00, 21.97) | Chlorambucil | 0.06 (0.00, 6.64) | 0.02 (0.00, 5.87) | 0.04 (0.00, 9.87) | 0.09 (0.00, 21.97) | 0.06 (0.00, 26.96) | 0.29 (0.00, 184.86) | 0.01 (0.00, 6.11) | 0.01 (0.00, 8.78) | 0.02 (0.00, 10.00) |
| 1.01 (0.02, 47.51) | 15.88 (0.15, 1673.24) | Calcineurin inhibitor | 0.39 (0.01, 17.47) | 0.68 (0.02, 28.80) | 1.43 (0.03, 65.78) | 0.99 (0.01, 99.76) | 4.56 (0.05, 404.96) | 0.20 (0.00, 23.42) | 0.08 (0.00, 44.69) | 0.29 (0.00, 39.89) |
| 3.50 (0.03, 489.10) | 40.97 (0.17, 9847.57) | 2.58 (0.06, 116.32) | Rituximab | 1.76 (0.02, 199.37) | 3.70 (0.03, 447.65) | 2.56 (0.01, 591.46) | 11.78 (0.03, 4230.87) | 0.52 (0.00, 135.79) | 0.22 (0.00, 217.62) | 0.74 (0.00, 225.52) |
| 0.02 (0.00, 7.73) | 23.28 (0.10, 5348.11) | 1.47 (0.03, 61.91) | 0.57 (0.01, 64.38) | Calcineurin inhibitor plus rituximab | 2.10 (0.02, 241.53) | 1.46 (0.01, 321.11) | 6.69 (0.02, 2304.91) | 0.29 (0.00, 73.80) | 0.12 (0.00, 119.30) | 0.42 (0.00, 122.71) |
| 1.20 (0.21, 6.92) | 11.08 (0.05, 2697.28) | 0.70 (0.02, 32.04) | 0.27 (0.00, 32.75) | 0.48 (0.00, 54.72) | Mycophenolate mofetil | 0.69 (0.00, 162.02) | 3.18 (0.01, 1157.87) | 0.14 (0.00, 37.19) | 0.06 (0.00, 59.45) | 0.20 (0.00, 61.74) |
| 0.34 (0.00, 51.49) | 15.99 (0.04, 6895.98) | 1.01 (0.01, 101.22) | 0.39 (0.00, 90.14) | 0.69 (0.00, 151.56) | 1.44 (0.01, 337.54) | Mizoribine | 4.60 (0.01, 2856.84) | 0.20 (0.00, 94.30) | 0.09 (0.00, 136.30) | 0.29 (0.00, 154.43) |
| 0.43 (0.00, 639.86) | 3.48 (0.01, 2237.15) | 0.22 (0.00, 19.44) | 0.08 (0.00, 30.51) | 0.15 (0.00, 51.48) | 0.31 (0.00, 114.15) | 0.22 (0.00, 135.18) | Azathioprine | 0.04 (0.00, 30.44) | 0.02 (0.00, 41.33) | 0.06 (0.00, 49.44) |
| 3.10 (0.11, 91.03) | 79.16 (0.16, 38293.66) | 4.99 (0.04, 582.28) | 1.93 (0.01, 507.05) | 3.40 (0.01, 853.44) | 7.14 (0.03, 1898.18) | 4.95 (0.01, 2310.54) | 22.76 (0.03, 15762.22) | Steroid | 0.42 (0.01, 24.93) | 1.42 (0.00, 854.17) |
| 1.76 (0.06, 48.04) | 187.03 (0.11, 307139.54) | 11.78 (0.02, 6201.78) | 4.57 (0.00, 4536.08) | 8.03 (0.01, 7701.26) | 16.88 (0.02, 16940.70) | 11.69 (0.01, 18642.04) | 53.76 (0.02, 119449.05) | 2.36 (0.04, 139.15) | Placebo | 3.36 (0.00, 6618.24) |
| 0.07 (0.00, 54.78) | 55.58 (0.10, 30890.72) | 3.50 (0.03, 488.95) | 1.36 (0.00, 415.14) | 2.39 (0.01, 699.60) | 5.02 (0.02, 1553.62) | 3.48 (0.01, 1865.34) | 15.98 (0.02, 12620.78) | 0.70 (0.00, 421.10) | 0.30 (0.00, 584.50) | Non immunosuppressive therapy |

The table shows comparisons of serious infection among different treatment strategies. Data are odds ratio with 95% confidence interval within bracket. The table should be read from left to right. Risk estimate is for the column-defining treatment compared to the row-defining treatment. An odds ratio <1 indicates the column treatment is associated with lower odds of serious infection than the row treatment.

**Supplementary Table 8.10 Network estimates (odds ratios and 95% CI) of effects of treatment on bone marrow suppression**

| Cyclophosphamide | 8.20 (0.95, 71.18) | 2.88 (0.06, 137.62) | 1.11 (0.02, 50.67) | 2.04 (0.08, 55.63) | 0.11 (0.01, 0.92) | 1.76 (0.01, 613.53) | 0.07 (0.00, 1.34) | 0.04 (0.01, 0.14) | 0.60 (0.00, 87.24) | 0.32 (0.01, 8.23) |
| --- | --- | --- | --- | --- | --- | --- | --- | --- | --- | --- |
| 0.12 (0.01, 1.06) | Chlorambucil | 0.01 (0.00, 0.28) | 0.01 (0.00, 0.33) | 0.00 (0.00, 0.06) | 0.04 (0.00, 1.93) | 0.02 (0.00, 0.81) | 0.19 (0.00, 13.40) | 0.02 (0.00, 0.95) | 0.23 (0.00, 85.73) | 0.02 (0.00, 0.71) |
| 0.35 (0.01, 16.61) | 73.23 (3.57, 1501.57) | Calcineurin inhibitor | 0.64 (0.02, 23.63) | 0.35 (0.03, 4.07) | 2.88 (0.06, 137.61) | 1.38 (0.03, 57.73) | 13.59 (0.66, 281.86) | 1.57 (0.04, 67.70) | 16.61 (0.10, 2749.92) | 1.11 (0.02, 50.67) |
| 0.90 (0.02, 41.47) | 113.64 (3.01, 4291.31) | 1.55 (0.04, 56.909) | Rituximab | 0.55 (0.02, 13.04) | 4.47 (0.06, 349.86) | 2.13 (0.03, 149.03) | 21.09 (0.19, 2336.22) | 2.43 (0.03, 174.18) | 25.77 (0.05, 13360.76) | 1.72 (0.02, 129.44) |
| 0.49 (0.02, 13.32) | 206.63 (17.23, 2477.82) | 2.82 (0.25, 32.40) | 1.82 (0.08, 43.10) | Calcineurin inhibitor plus rituximab | 8.12 (0.25, 259.54) | 3.88 (0.14, 107.19) | 38.34 (0.78, 1878.89) | 4.43 (0.16, 126.13) | 46.85 (0.16, 13481.83) | 3.12 (0.10, 95.09) |
| 8.93 (1.08, 73.69) | 25.44 (0.52, 1249.65) | 0.35 (0.01, 16.60) | 0.22 (0.00, 17.53) | 0.12 (0.00, 3.93) | Mycophenolate mofetil | 0.48 (0.01, 41.86) | 4.72 (0.03, 642.43) | 0.54 (0.01, 48.87) | 5.77 (0.01, 3497.07) | 0.38 (0.00, 36.22) |
| 0.57 (0.00, 197.83) | 53.24 (1.23, 2298.50) | 0.73 (0.02, 30.51) | 0.47 (0.01, 32.70) | 0.26 (0.01, 7.12) | 2.09 (0.02, 183.35) | Mizoribine | 9.88 (0.08, 1214.49) | 1.14 (0.01, 91.49) | 12.07 (0.02, 6771.92) | 0.80 (0.01, 67.90) |
| 13.85 (0.75, 256.48) | 5.39 (0.07, 389.10) | 0.07 (0.00, 1.53) | 0.05 (0.00, 5.25) | 0.03, (0.00, 1.28) | 0.21 (0.00, 28.84) | 0.10 (0.00, 12.45) | Azathioprine | 0.12 (0.00, 14.51) | 1.22 (0.02, 74.70) | 0.08 (0.00, 10.71) |
| 25.19 (7.39, 85.82) | 46.68 (1.05, 2071.56) | 0.64 (0.01, 27.50) | 0.41 (0.01, 29.38) | 0.23 (0.01, 6.44) | 1.83 (0.02, 164.53) | 0.88 (0.01, 70.33) | 8.66 (0.07, 1088.07) | ACTH | 10.58 (0.02, 6035.78) | 0.70 (0.01, 60.94) |
| 1.68 (0.01, 246.03) | 4.41 (0.01, 1667.22) | 0.06 (0.00, 9.97) | 0.04 (0.00, 20.12) | 0.02 (0.00, 6.14) | 0.17 (0.00, 105.11) | 0.08 (0.00, 46.47) | 0.82 (0.01, 50.02) | 0.09 (0.00, 53.88) | Placebo | 0.07 (0.00, 39.35) |
| 3.10 (0.12, 79.18) | 66.25 (1.41, 3122.36) | 0.90 (0.02, 41.47) | 0.58 (0.01, 44.00) | 0.32 (0.01, 9.78) | 2.60 (0.03, 245.70) | 1.24 (0.01, 105.17) | 12.29 (0.09, 1619.17) | 1.42 (0.02, 122.79) | 15.02 (0.03, 8882.01) | Non immunosuppressive therapy |

Abbreviations: ACTH = adrenocorticotropic hormone

The table shows comparisons of bone marrow suppression among different treatment strategies. Data are odds ratio with 95% confidence interval within bracket. The table should be read from left to right. Risk estimate is for the column-defining treatment compared to the row-defining treatment. An odds ratio <1 indicates the column treatment is associated with lower odds of bone marrow suppression than the row treatment.

**Supplementary Table 9 Estimated treatment effects and ratings of evidence certainty**

**Supplementary Table 9.1 Complete remission**

|  |  |  | **Direct estimate** |  | **Indirect estimate** |  | **Network estimate** |  |
| --- | --- | --- | --- | --- | --- | --- | --- | --- |
| **Intervention** | **Comparator** | **Number of trials (number of participants)** | **Odds ratio (95% confidence interval)** | **Rating** | **Odds ratio (95% confidence interval)** | **Rating** | **Odds ratio (95% confidence interval)** | **Rating** |
| Cyclophosphamide | Chlorambucil | 3 (127) | 1.91 (0.84 to 4.35) | Low | 2.05 (0.56 to 7.56) | Low | 1.13 (0.46 to 2.75) | Low |
| Cyclophosphamide | Calcineurin inhibitor | 3 (225) | 0.94 (0.23 to 3.77) | Low | 0.46 (0.11 to 1.90) | Low | 1.26 (0.61 to 2.63) | Low |
| Cyclophosphamide | Rituximab | 1 (57) | 0.75 (0.26 to 2.19) | Low | 10.22 (1.40 to 74.31) | Moderate | 0.35 (0.10 to 1.24) | Low |
| Cyclophosphamide | Calcineurin inhibitor plus rituximab | 1 (86) | 4.45 (1.78 to 11.14) | Low | … | … | 4.45 (1.04 to 19.10) | Moderate |
| Cyclophosphamide | Steroid | 3 (123) | 5.04 (1.40 to 18.08) | Low | … | … | 2.31 (0.62 to 8.52) | Low |
| Cyclophosphamide | Mycophenolate mofetil | 3 (78) | 0.64 (0.21 to 1.96) | Low | 4.67 (1.34 to 16.23) | Low | 1.81 (0.69 to 4.71) | Low |
| Cyclophosphamide | Mizoribine | 1 (55) | 1.97 (0.63 to 6.11) | Low | … | … | 1.97 (0.40 to 9.75) | Low |
| Cyclophosphamide | ACTH | 1 (32) | 0.33 (0.07 to 1.49) | Low | … | … | 0.33 (0.05 to 2.18) | Low |
| Cyclophosphamide | Leflunomide | 1 (48) | 4.20 (0.97 to 18.18) | Very low | … | … | 4.20 (0.66 to 26.74) | Very low |
| Cyclophosphamide | Non-immunosuppressive therapy | 2 (158) | 1.95 (0.89 to 4.31) | Low | 0.24 (0.09 to 0.64) | Low | 3.14 (1.46 to 6.79) | Moderate |

… Not estimable

**Supplementary Table 9.2 Partial remission**

|  |  |  | **Direct estimate** |  | **Indirect estimate** |  | **Network estimate** |  |
| --- | --- | --- | --- | --- | --- | --- | --- | --- |
| **Intervention** | **Comparator** | **Number of trials (number of participants)** | **Odds ratio (95% confidence interval)** | **Rating** | **Odds ratio (95% confidence interval)** | **Rating** | **Odds ratio (95% confidence interval)** | **Rating** |
| Cyclophosphamide | Chlorambucil | 3 (127) | 1.55 (0.22 to 10.81) | Low | 0.53 (0.16 to 1.81) | Low | 1.51 (0.68 to 3.35) | Low |
| Cyclophosphamide | Calcineurin inhibitor | 3 (225) | 1.62 (0.92 to 2.87) | Low | 1.77 (0.63 to 4.96) | Low | 1.15 (0.61 to 2.16) | Low |
| Cyclophosphamide | Rituximab | 1 (57) | 1.12 (0.39 to 3.21) | Low | 0.99 (0.33 to 3.00) | Low | 1.04 (0.45 to 2.40) | Low |
| Cyclophosphamide | Calcineurin inhibitor plus rituximab | 1 (86) | 0.63 (0.24 to 1.63) | Low | … | … | 0.63 (0.17 to 2.30) | Low |
| Cyclophosphamide | Mycophenolate mofetil | 3 (78) | 1.79 (0.56 to 5.67) | Low | 1.29 (0.39 to 4.21) | Low | 1.18 (0.51 to 2.76) | Low |
| Cyclophosphamide | Mizoribine | 1 (55) | 1.91 (0.29 to 12.44) | Low | … | … | 1.91 (0.24 to 15.16) | Low |
| Cyclophosphamide | ACTH | 1 (32) | 1.67 (0.41 to 6.62) | Low | … | … | 1.67 (0.32 to 8.79) | Low |
| Cyclophosphamide | Non-immunosuppressive therapy | 2 (158) | 1.43 (0.31 to 6.64) | Low | 0.32 (0.17 to 0.17) | Low | 2.17 (1.06 to 4.45) | High |

… Not estimable

**Supplementary Table 9.3 Kidney failure**

|  |  |  | **Direct estimate** |  | **Indirect estimate** |  | **Network estimate** |  |
| --- | --- | --- | --- | --- | --- | --- | --- | --- |
| **Intervention** | **Comparator** | **Number of trials (number of participants)** | **Odds ratio (95% confidence interval)** | **Rating** | **Odds ratio (95% confidence interval)** | **Rating** | **Odds ratio (95% confidence interval)** | **Rating** |
| Cyclophosphamide | Chlorambucil | 1 (18) | 6.40 (0.55, 74.89) | Low | 4.23 (0.57 to 31.30) | Low | 4.99 (1.06 to 23.56) | Low |
| Cyclophosphamide | Calcineurin inhibitor | 2 (117) | 1.02 (0.06 to 16.66) | Very low | 1.65 (0.29 to 9.39) | Very low | 0.70 (0.16 to 3.08) | Low |
| Cyclophosphamide | Rituximab | 1 (70) | 4.71 (0.22 to 101.87) | Very low | … | … | 4.71 (0.22 to 101.87) | Low |
| Cyclophosphamide | Calcineurin inhibitor plus rituximab | 1 (88) | 3.07 (0.12 to 77.50) | Very low | … | … | 3.07 (0.12 to 77.50) | Low |
| Cyclophosphamide | Steroid | 2 (97) | 0.79 (0.20 to 3.12) | Low | 8.04 (0.31 to 209.58) | Very low | 0.60 (0.17 to 2.12) | Very low |
| Cyclophosphamide | Mycophenolate mofetil | 1 (59) | 1.04 (0.02 to 53.95) | Very low | 1.47 (0.07 to 32.34) | Very low | 0.80 (0.07 to 9.07) | Very low |
| Cyclophosphamide | ACTH | 1 (34) | 0.31 (0.01, 8.28) | Very low | … | … | 0.31 (0.01 to 8.28) | Very low |
| Cyclophosphamide | Non-immunosuppressive therapy | 2 (140) | 0.23 (0.02 to 2.15) | Low | 0.60 (0.10 to 3.69) | Low | 0.76 (0.18 to 3.11) | Low |

… Not estimable

**Supplementary Table 9.4 Doubling of serum creatinine**

|  |  |  | **Direct estimate** |  | **Indirect estimate** |  | **Network estimate** |  |
| --- | --- | --- | --- | --- | --- | --- | --- | --- |
| **Intervention** | **Comparator** | **Number of trials (number of participants)** | **Odds ratio (95% confidence interval)** | **Rating** | **Odds ratio (95% confidence interval)** | **Rating** | **Odds ratio (95% confidence interval)** | **Rating** |
| Cyclophosphamide | Chlorambucil | 2 (45) | 0.69 (0.06, 8.33) | Low | … | … | 0.63 (0.08 to 5.23) | Low |
| Cyclophosphamide | Calcineurin inhibitor | 2 (117) | 1.02 (0.06 to 16.66) | Low | 1.05 (0.06 to 19.25) | Low | 0.99 (0.14 to 7.14) | Low |
| Cyclophosphamide | Steroid | 1 (73) | 0.13 (0.01 to 2.56) | Very low | 0.67 (0.03 to 15.42) | Low | 0.42 (0.04 to 4.27) | Low |
| Cyclophosphamide | Mycophenolate mofetil | 1 (59) | 1.04 (0.02 to 53.95) | Very low | 1.10 (0.00 to 6486.58) | Very low | 1.01 (0.03 to 40.51) | Low |
| Cyclophosphamide | Non-immunosuppressive therapy | 1 (54) | 0.48 (0.04 to 5.64) | Very low | 18.45 (0.64 to 534.52) | Very low | 0.21 (0.03 to 1.73) | Low |

… Not estimable

**Supplementary Table 9.5 End of treatment proteinuria**

|  |  |  | **Direct estimate** |  | **Indirect estimate** |  | **Network estimate** |  |
| --- | --- | --- | --- | --- | --- | --- | --- | --- |
| **Intervention** | **Comparator** | **Number of trials (number of participants)** | **Mean difference grams/24 hours (95% confidence interval)** | **Rating** | **Mean difference grams/24 hours (95% confidence interval)** | **Rating** | **Mean difference grams/24 hours (95% confidence interval)** | **Rating** |
| Cyclophosphamide | Chlorambucil | 1 (87) | 1.52 (0.0005 to 4629.137) | Low | 0.70 (0.00 to >1000) | Very low | 2.19 (0.00 to >1000) | Very low |
| Cyclophosphamide | Calcineurin inhibitor | 5 (259) | 0.46 (0.03 to 6.24) | Low | 0.30 (0.00 to >1000) | Very low | 1.53 (0.00 to >1000) | Very low |
| Cyclophosphamide | Steroid | 1 (11) | 0.12 (0.00 to >1000) | Very low | 1.65 (0.03 to 101.68) | Very low | 0.07 (0.00 to >1000) | Very low |
| Cyclophosphamide | Mycophenolate mofetil | 1 (57) | 0.78 (0.00 to >1000) | Very low | 0.88 (0.00 to >1000) | Very low | 1.25 (0.00 to >1000) | Very low |
| Cyclophosphamide | Non-immunosuppressive therapy | 1 (20) | 1.61 (0.04 to 69.81) | Very low | 0.40 (0.00 to >1000) | Very low | 4.12 (0.00 to >1000) | Very low |

… Not estimable

**Supplementary Table 9.6 Serious adverse events**

|  |  |  | **Direct estimate** |  | **Indirect estimate** |  | **Network estimate** |  |
| --- | --- | --- | --- | --- | --- | --- | --- | --- |
| **Intervention** | **Comparator** | **Number of trials (number of participants)** | **Odds ratio (95% confidence interval)** | **Rating** | **Odds ratio (95% confidence interval)** | **Rating** | **Odds ratio (95% confidence interval)** | **Rating** |
| Cyclophosphamide | Chlorambucil | 1 (95) | 0.34 (0.07 to 1.78) | Low | 1.32 (0.23 to 7.71) | Low | 0.57 (0.15 to 2.20) | Low |
| Cyclophosphamide | Calcineurin inhibitor | 3 (191) | 0.50 (0.05 to 5.20) | Low | 0.85 (0.16 to 4.36) | Low | 1.46 (0.54 to 3.98) | Low |
| Cyclophosphamide | Rituximab | 1 (74) | 0.67 (0.19 to 2.34) | Low | 0.27 (0.05 to 1.30) | Low | 1.87 (0.54 to 6.51) | Low |
| Cyclophosphamide | Calcineurin inhibitor plus rituximab | 1 (86) | 1.41 (0.44 to 4.47) | Low | … | … | 1.41 (0.22 to 9.23) | Low |
| Cyclophosphamide | Steroid | 1 (90) | 0.91 (0.23 to 3.62) | Low | 1.91 (0.00 to >1000) | Very low | 0.91 (0.12 to 6.88) | Low |
| Cyclophosphamide | Mycophenolate mofetil | 1 (60) | 1.50 (0.54 to 4.17) | Low | 0.23 (0.03 to 1.59) | Low | 2.48 (0.67 to 9.13) | Low |
| Cyclophosphamide | Mizoribine | 1 (55) | 1.45 (0.41 to 5.18) | Low | … | … | 1.45 (0.21 to 10.25) | Low |
| Cyclophosphamide | Non-immunosuppressive therapy | 2 (242) | 13.09 (2.40 to 71.26) | Low | 0.53 (0.17 to 1.66) | Low | 3.36 (1.09 to 10.35) | Moderate |

… Not estimable

**Supplementary Table 9.7 Discontinuation of treatment**

|  |  |  | **Direct estimate** |  | **Indirect estimate** |  | **Network estimate** |  |
| --- | --- | --- | --- | --- | --- | --- | --- | --- |
| **Intervention** | **Comparator** | **Number of trials (number of participants)** | **Odds ratio (95% confidence interval)** | **Rating** | **Odds ratio (95% confidence interval)** | **Rating** | **Odds ratio (95% confidence interval)** | **Rating** |
| Cyclophosphamide | Chlorambucil | 1 (95) | 2.93 (0.55 to 15.55) | Low | 0.08 (0.02 to 0.43) | Moderate | 0.17 (0.05 to 0.54) | Moderate |
| Cyclophosphamide | Calcineurin inhibitor | 3 (243) | 2.05 (0.85 to 4.97) | Low | 0.79 (0.06 to 10.51) | Low | 0.54 (0.23 to 1.23) | Low |
| Cyclophosphamide | Steroid | 1 (90) | 2.23 (0.76 to 6.52) | Low | 0.26 (0.02 to 3.71) | Low | 0.61 (0.21 to 1.76) | Low |
| Cyclophosphamide | Non-immunosuppressive therapy | 2 (72) | 0.77 (0.27 to 2.24) | Low | 1.26 (0.23 to 6.76) | Low | 1.13 (0.48 to 2.67) | Low |

… Not estimable

**Supplementary 9.8 Serious infection**

|  |  |  | **Direct estimate** |  | **Indirect estimate** |  | **Network estimate** |  |
| --- | --- | --- | --- | --- | --- | --- | --- | --- |
| **Intervention** | **Comparator** | **Number of trials (number of participants)** | **Odds ratio (95% confidence interval)** | **Rating** | **Odds ratio (95% confidence interval)** | **Rating** | **Odds ratio (95% confidence interval)** | **Rating** |
| Cyclophosphamide | Chlorambucil | 1 (97) | 0.08 (0.00 to 1.38) | Low | 0.56 (0.09 to 3.41) | Low | 0.08 (0.00 to 21.97) | Low |
| Cyclophosphamide | Calcineurin inhibitor | 6 (379) | 1.40 (0.44 to 4.45) | Low | 1.24 (0.17 to 9.33) | Low | 1.01 (0.02 to 47.51) | Low |
| Cyclophosphamide | Rituximab | 1 (68) | 3.10 (0.36 to 9.37) | Very low | 1.22 (0.15 to 10.10) | Low | 3.50 (0.03 to 489.10) | Low |
| Cyclophosphamide | Calcineurin inhibitor plus rituximab | 1 (86) | 1.76 (0.75 to 4.14) | Low | … | … | 0.02 (0.00 to 7.73) | Low |
| Cyclophosphamide | Steroid | 1 (90) | 5.99 (0.28 to 128.33) | Very low | 1.45 (0.00 to >1000) | Very low | 1.20 (0.21 to 6.92) | Very low |
| Cyclophosphamide | Mycophenolate mofetil | 1 (60) | 0.84 (0.26 to 2.70) | Low | 0.66 (0.07 to 5.96) | Low | 0.34 (0.00 to 51.49) | Low |
| Cyclophosphamide | Mizoribine | 1 (55) | 1.21 (0.07 to 20.35) | Very low | … | … | 0.43 (0.00 to 639.86) | Very low |
| Cyclophosphamide | Non-immunosuppressive therapy | 1 (22) | 1.00 (0.02 to 55.27) | Low | 0.70 (0.12 to 4.28) | Low | 0.07 (0.00 to 54.78) | Low |

…Not estimable

**Supplementary Table 9.9 Bone marrow suppression**

|  |  |  | **Direct estimate** |  | **Indirect estimate** |  | **Network estimate** |  |
| --- | --- | --- | --- | --- | --- | --- | --- | --- |
| **Intervention** | **Comparator** | **Number of trials (number of participants)** | **Odds ratio (95% confidence interval)** | **Rating** | **Odds ratio (95% confidence interval)** | **Rating** | **Odds ratio (95% confidence interval)** | **Rating** |
| Cyclophosphamide | Chlorambucil | 2 (117) | 0.12 (0.01 to 1.06) | Low | 0.24 (0.05 to 1.19) | Low | 0.12 (0.01 to 1.06) | Low |
| Cyclophosphamide | Calcineurin inhibitor | 4 (212) | 6.85 (1.46 to 32.19) | Low | 5.91 (0.77 to 45.11) | Low | 0.35 (0.01 to 16.61) | Low |
| Cyclophosphamide | Rituximab | 1 (70) | 13.85 (0.75 to 256. 48) | Very low | 8.53 (0.13 to 551.68) | Very low | 0.90 (0.02 to 41.47) | Very low |
| Cyclophosphamide | Calcineurin inhibitor plus rituximab | 1 (86) | 25.19 (7.39 to 85.82) | Low | … | … | 0.49 (0.02 to 13.32) | Low |
| Cyclophosphamide | Mycophenolate mofetil | 2 (84) | 3.10 (0.12 to 79.23) | Low | 7.02 (0.73 to 67.57) | Low | 8.93 (1.08 to 73.69) | Moderate |
| Cyclophosphamide | Mizoribine | 1 (57) | 6.49 (0.30 to 141.71) | Very low | … | … | 0.57 (0.00 to 197.83) | Very low |
| Cyclophosphamide | ACTH | 1 (34) | 5.69 (0.25 to 128.50) | Very low | … | … | 25.19 (7.39 to 85.82) | Moderate |
| Cyclophosphamide | Non-immunosuppressive therapy | 1 (22) | 1.00 (0.02, 55.27) | Low | 7.36 (1.45 to 37.40) | Moderate | 3.10 (0.12 to 79.18) | Low |

… Not estimable

## Supplementary Figure 1: Networks of secondary outcomes

### Supplementary Figure 1.1. Complete remission and partial remission at 6 months


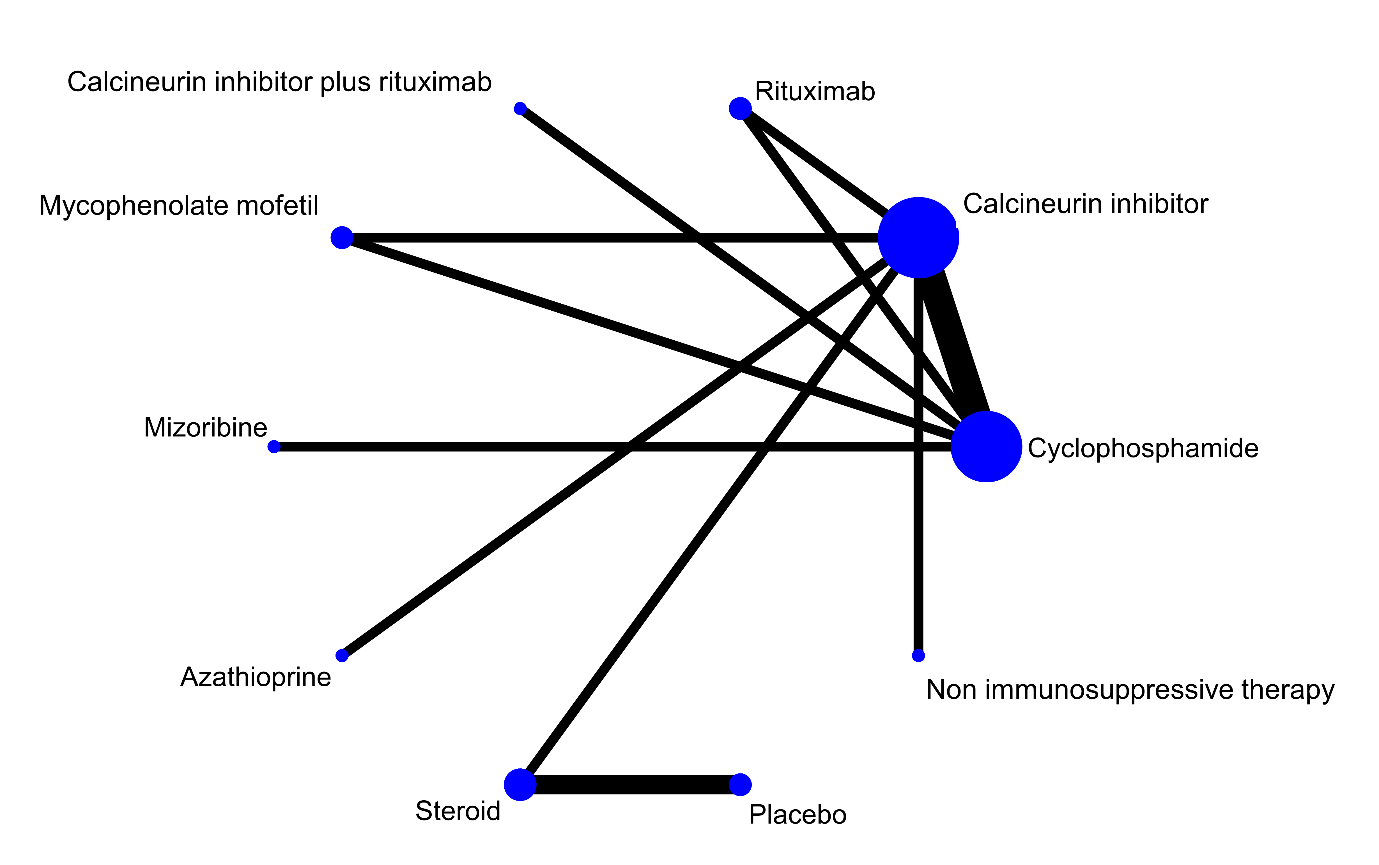


The size of the node corresponds to the number of trials. The thickness of the line connecting two treatments corresponds to the number of patients. Numbers next to each line represent the number of participants and number of trials that compared the connected treatments.

**Supplementary Figure 1.2. Kidney Failure**


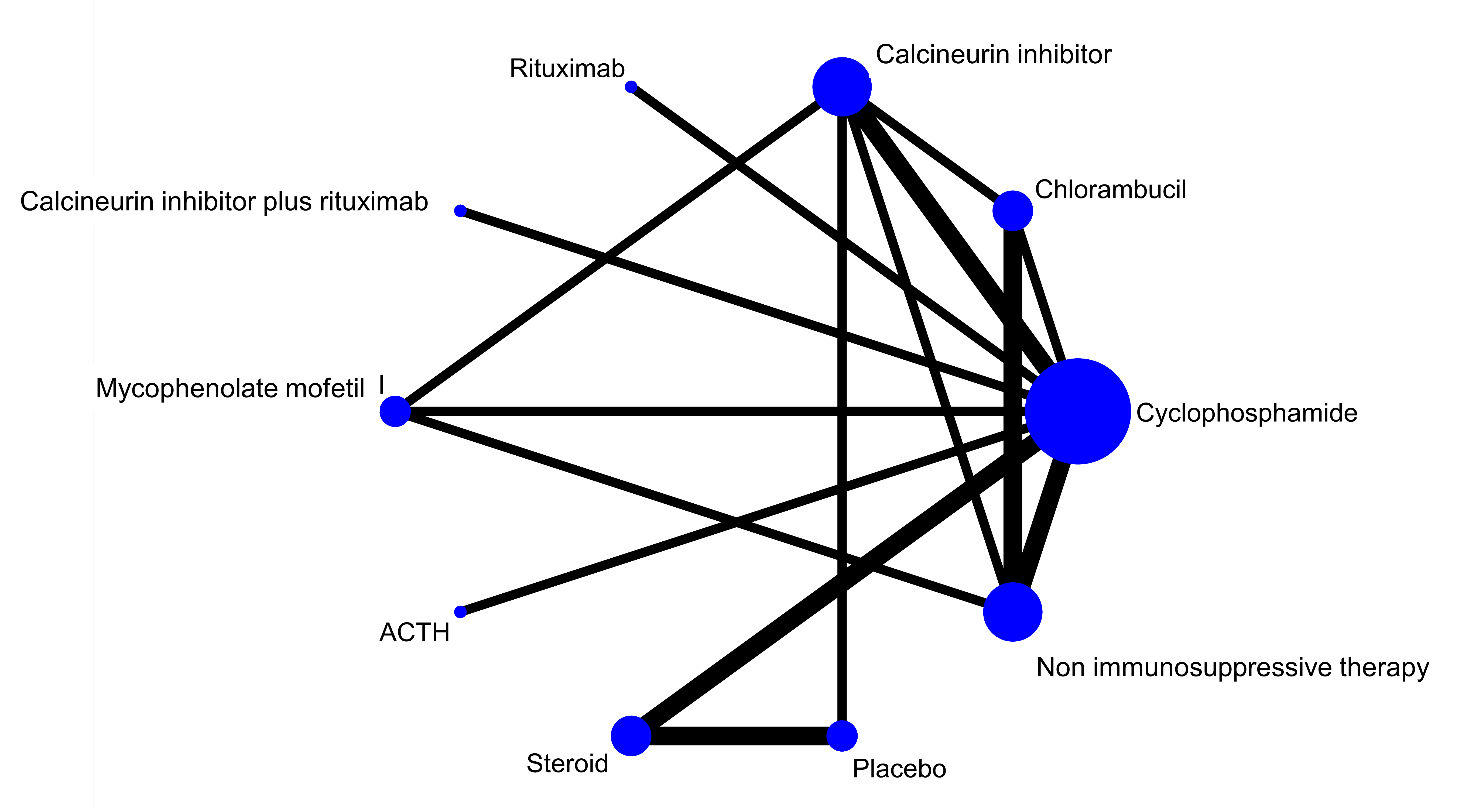


The size of the node corresponds to the number of trials. The thickness of the line connecting two treatments corresponds to the number of patients. Numbers next to each line represent the number of participants and number of trials that compared the connected treatments. Abbreviations: ACTH = adrenocorticotropic hormone

**Supplementary Figure 1.3. Doubling of Creatinine**


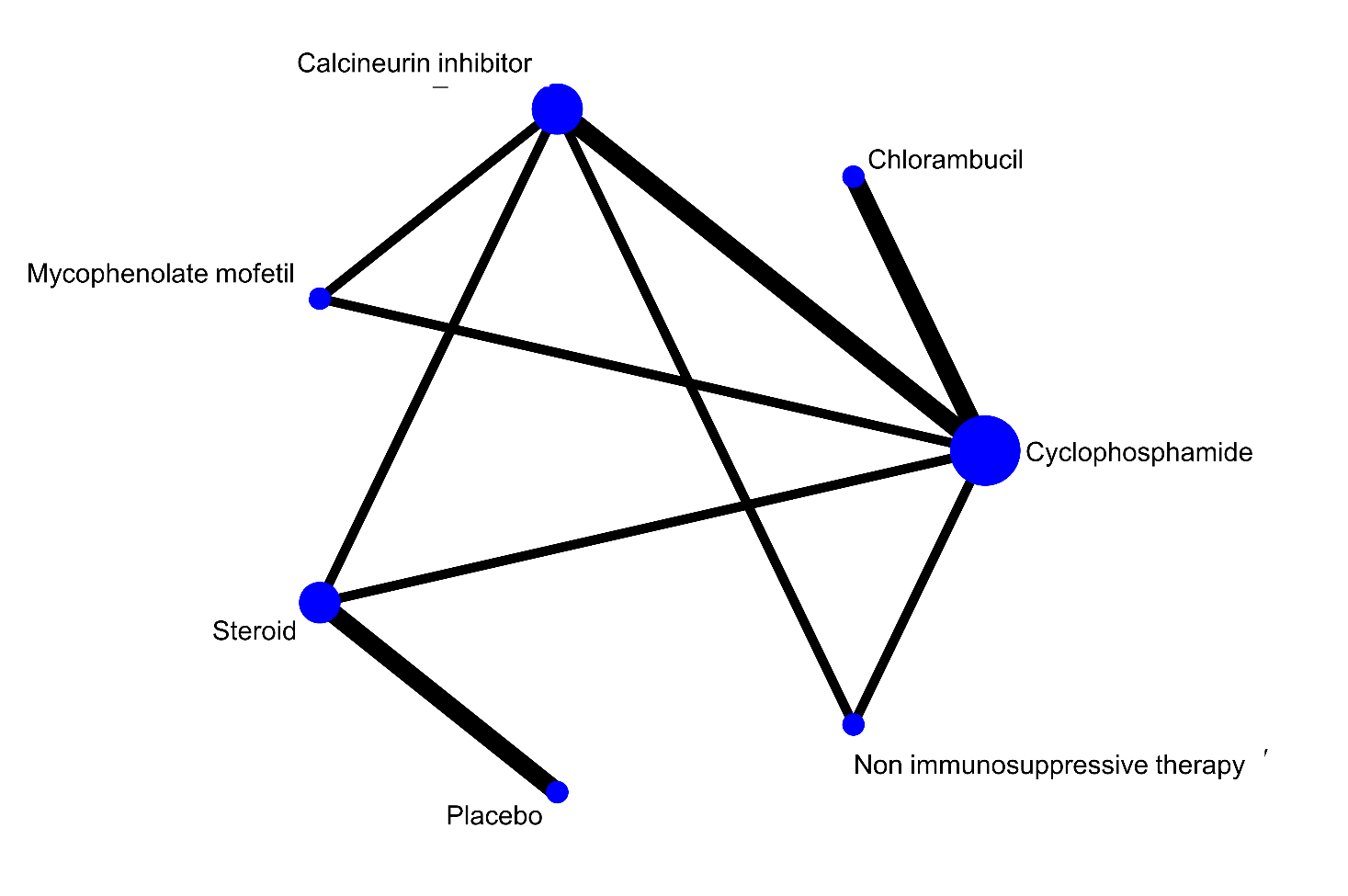


The size of the node corresponds to the number of trials. The thickness of the line connecting two treatments corresponds to the number of patients. Numbers next to each line represent the number of participants and number of trials that compared the connected treatments.

**Supplementary Figure 1.4. End of treatment glomerular filtration rate**

Calcineurin inhibitor

Azathioprine

Steroid

Placebo

Non immunosuppressive therapy

The size of the node corresponds to the number of trials. The thickness of the line connecting two treatments corresponds to the number of patients. Numbers next to each line represent the number of participants and number of trials that compared the connected treatments.

**Supplementary Figure 1.5. End of treatment proteinuria**


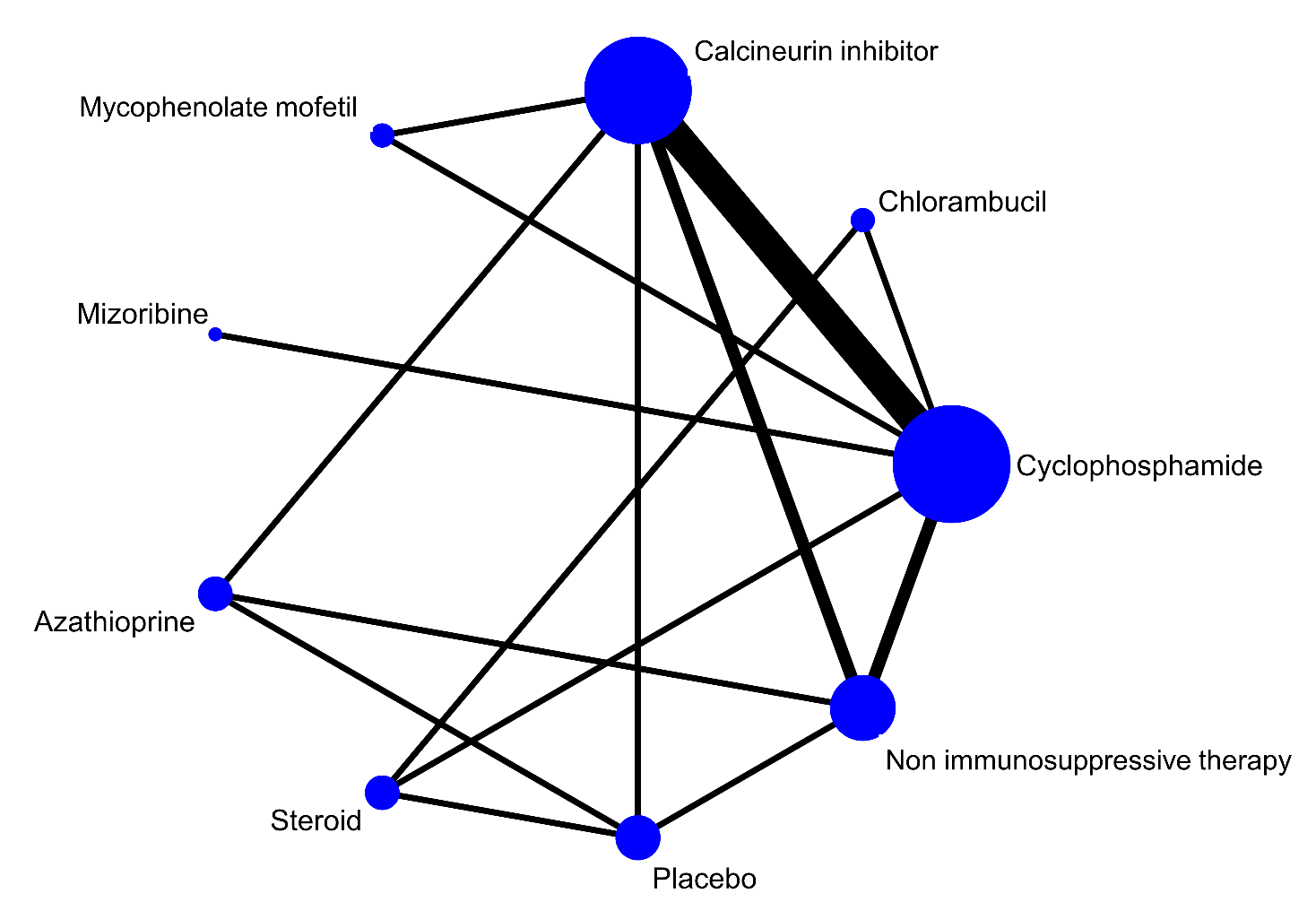


The size of the node corresponds to the number of trials. The thickness of the line connecting two treatments corresponds to the number of patients. Numbers next to each line represent the number of participants and number of trials that compared the connected treatments.

**Supplementary Figure 1.6. Serious Adverse Events**


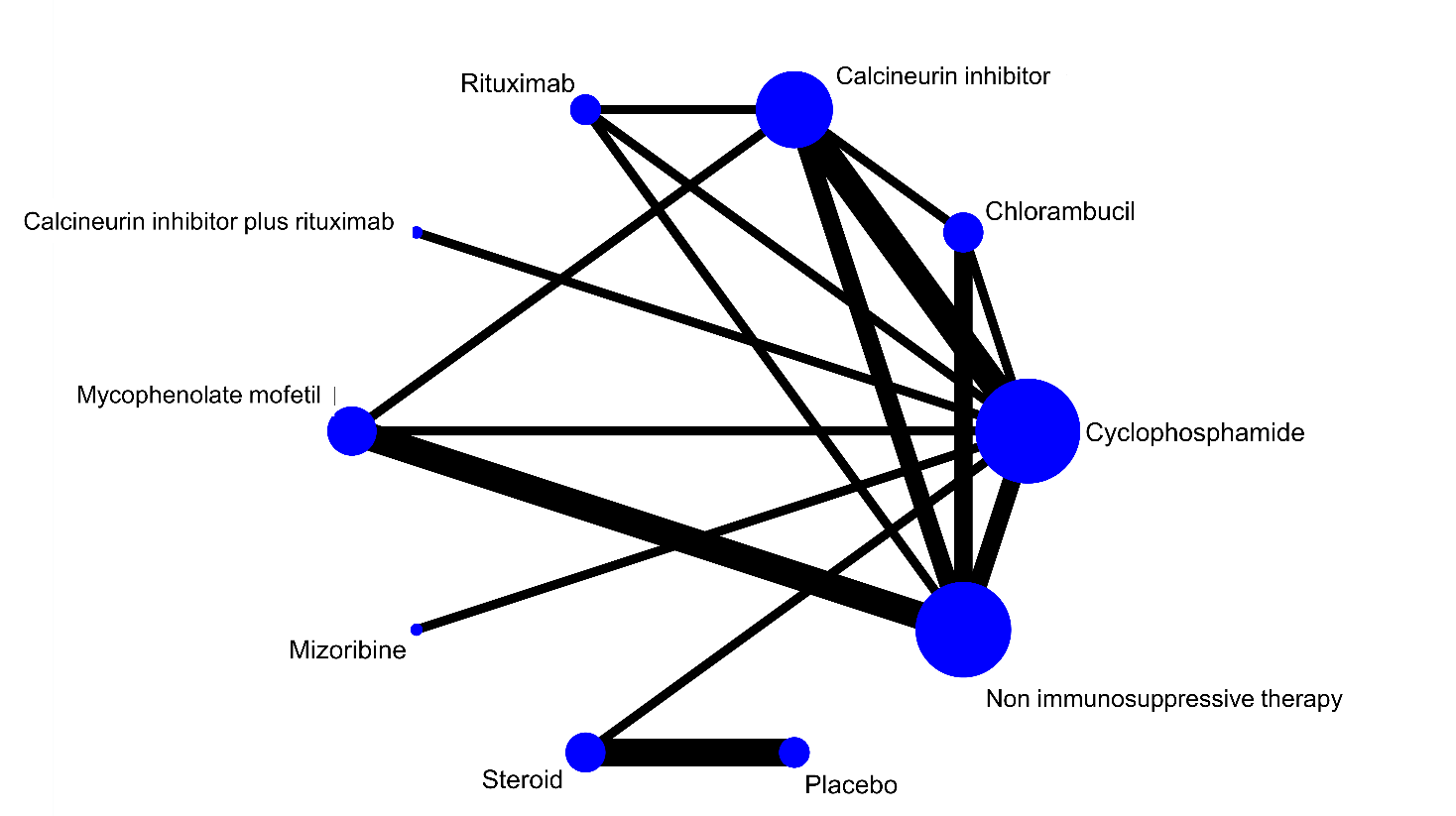


The size of the node corresponds to the number of trials. The thickness of the line connecting two treatments corresponds to the number of patients. Numbers next to each line represent the number of participants and number of trials that compared the connected treatments.

**Supplementary Figure 1.7. Discontinuation of treatment**


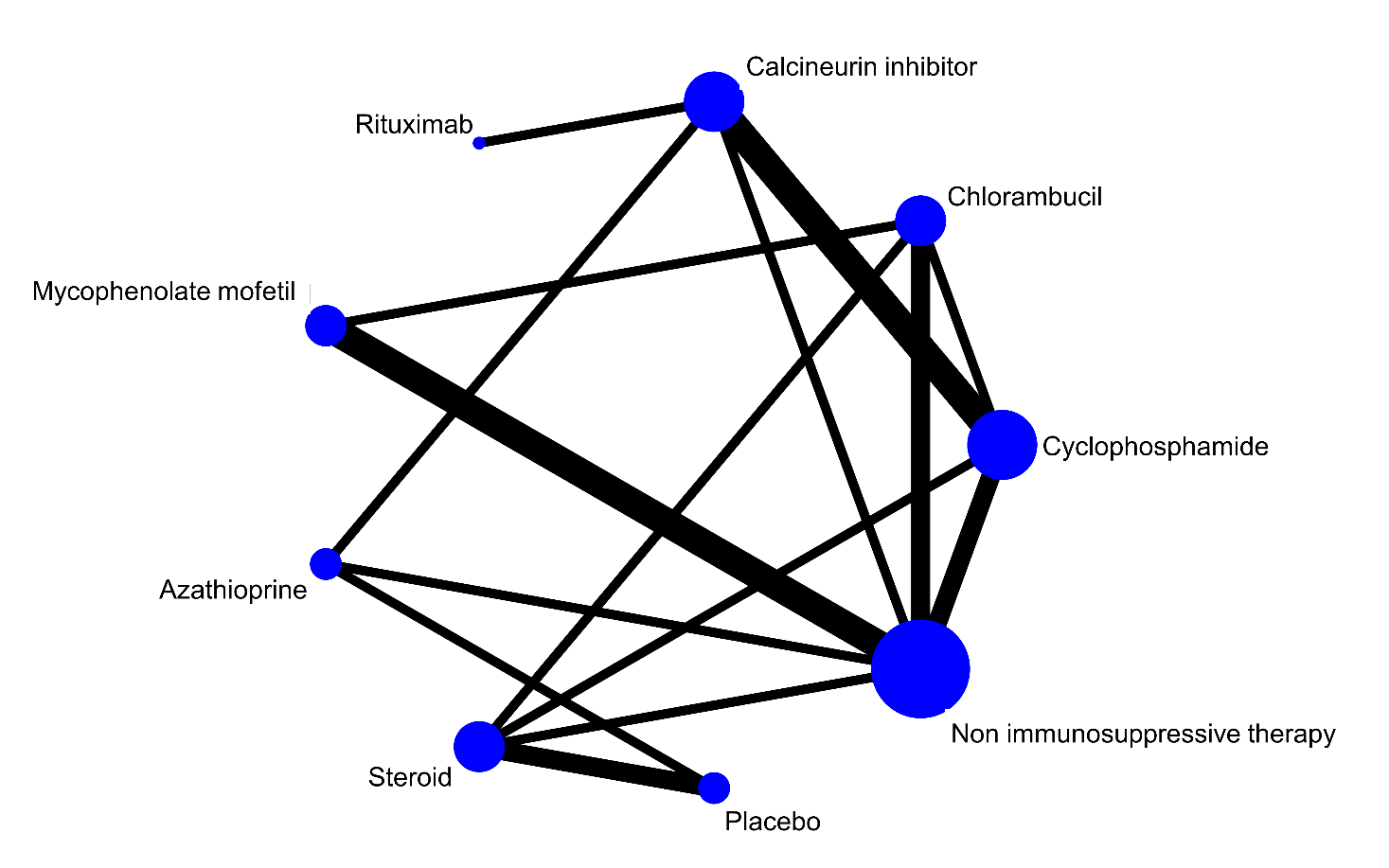


The size of the node corresponds to the number of trials. The thickness of the line connecting two treatments corresponds to the number of patients. Numbers next to each line represent the number of participants and number of trials that compared the connected treatments.

**Supplementary Figure 1.8. Serious Infections**


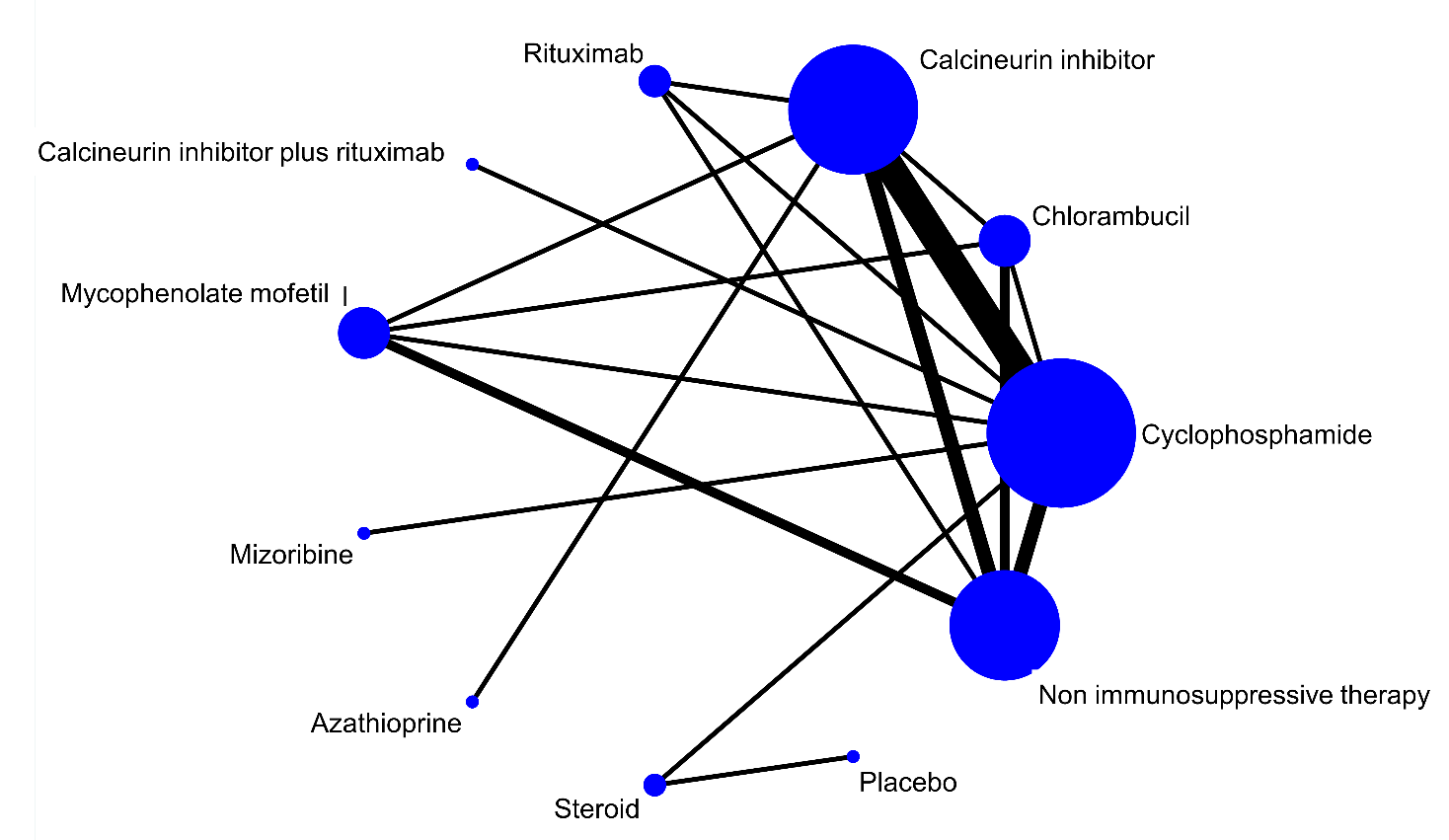


The size of the node corresponds to the number of trials. The thickness of the line connecting two treatments corresponds to the number of patients. Numbers next to each line represent the number of participants and number of trials that compared the connected treatments.

**Supplementary Figure 1.9. Bone marrow suppression**


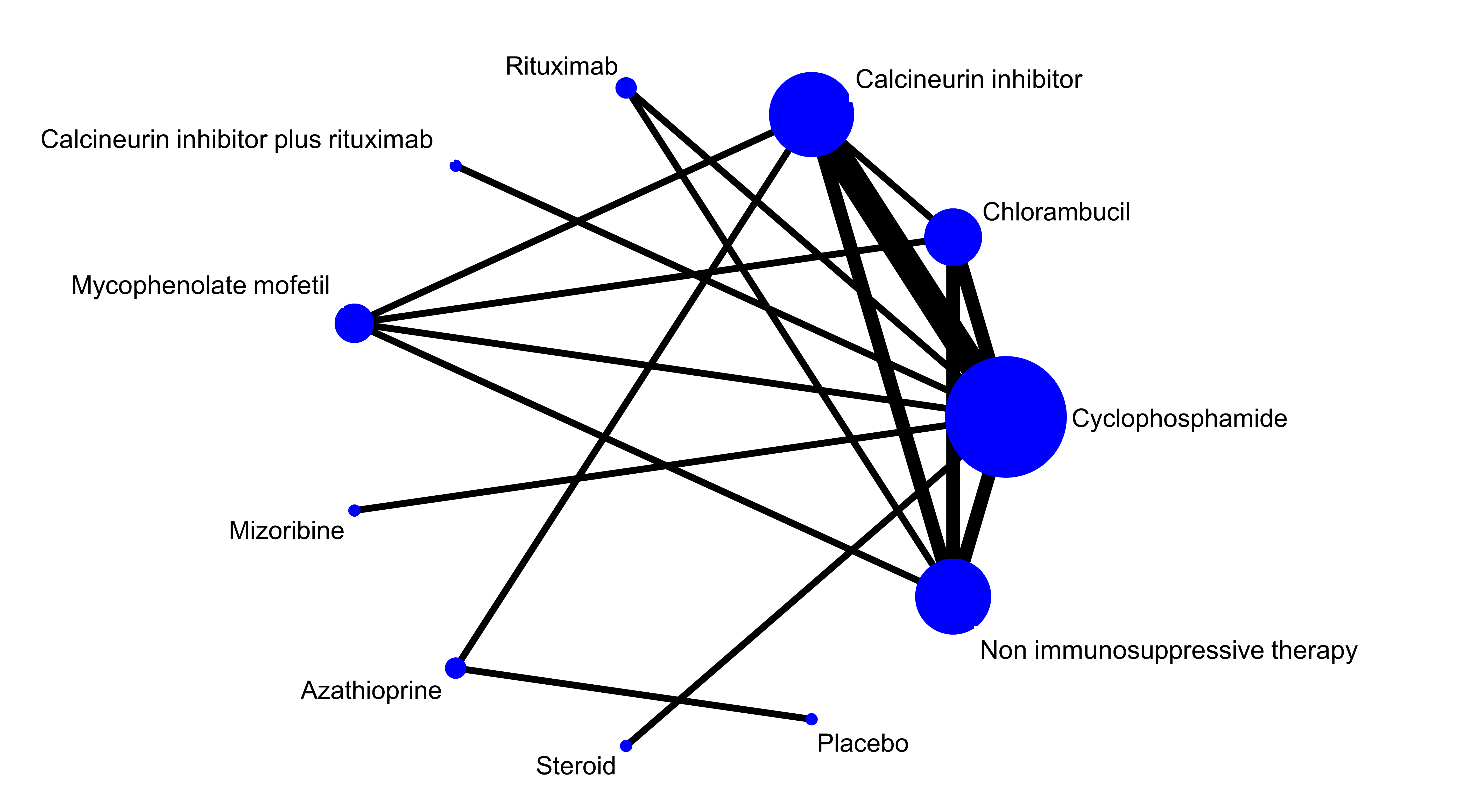


The size of the node corresponds to the number of trials. The thickness of the line connecting two treatments corresponds to the number of patients. Numbers next to each line represent the number of participants and number of trials that compared the connected treatments.

**Supplementary Figure 2: Rankograms**

**Supplementary Figure 2.1. Complete remission**

**
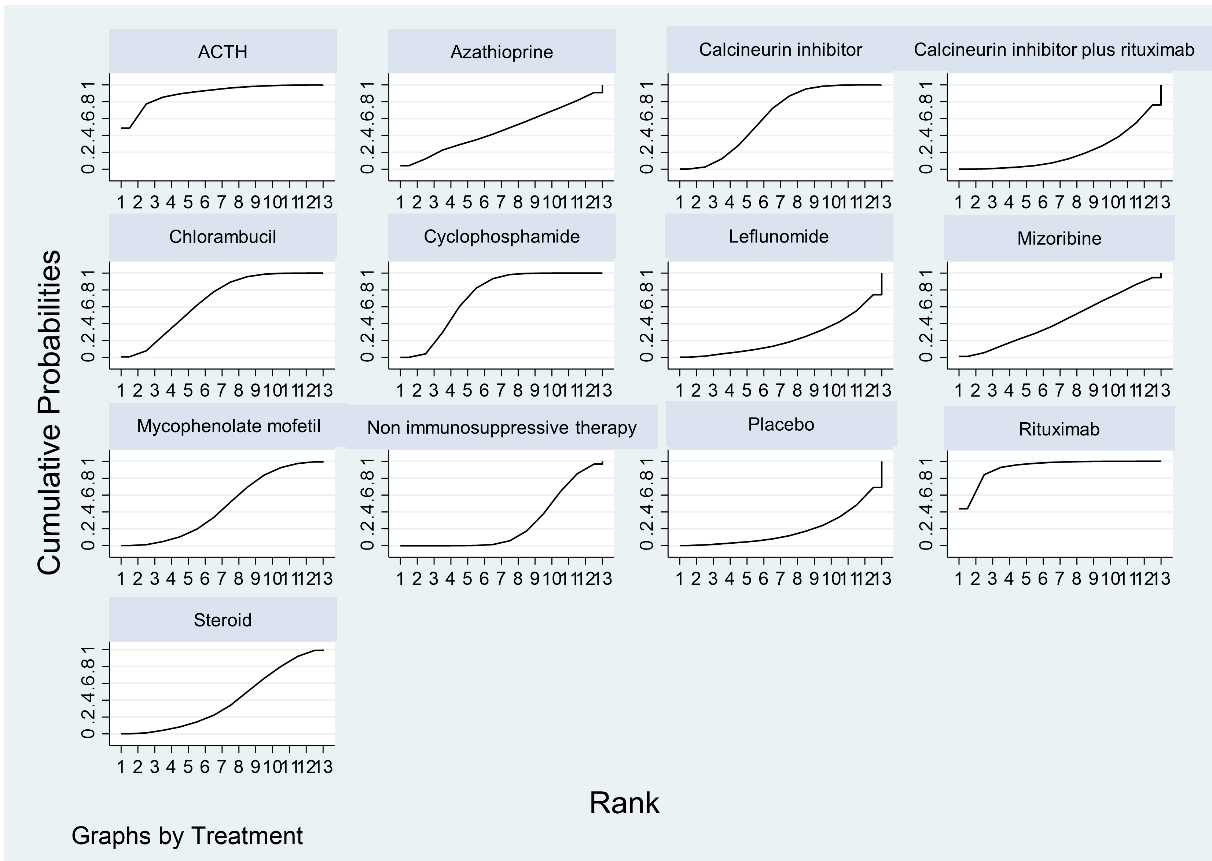
**

Abbreviations: ACTH = adrenocorticotropic hormone

**Supplementary Figure 2.2 Complete remission at 6 months**

**
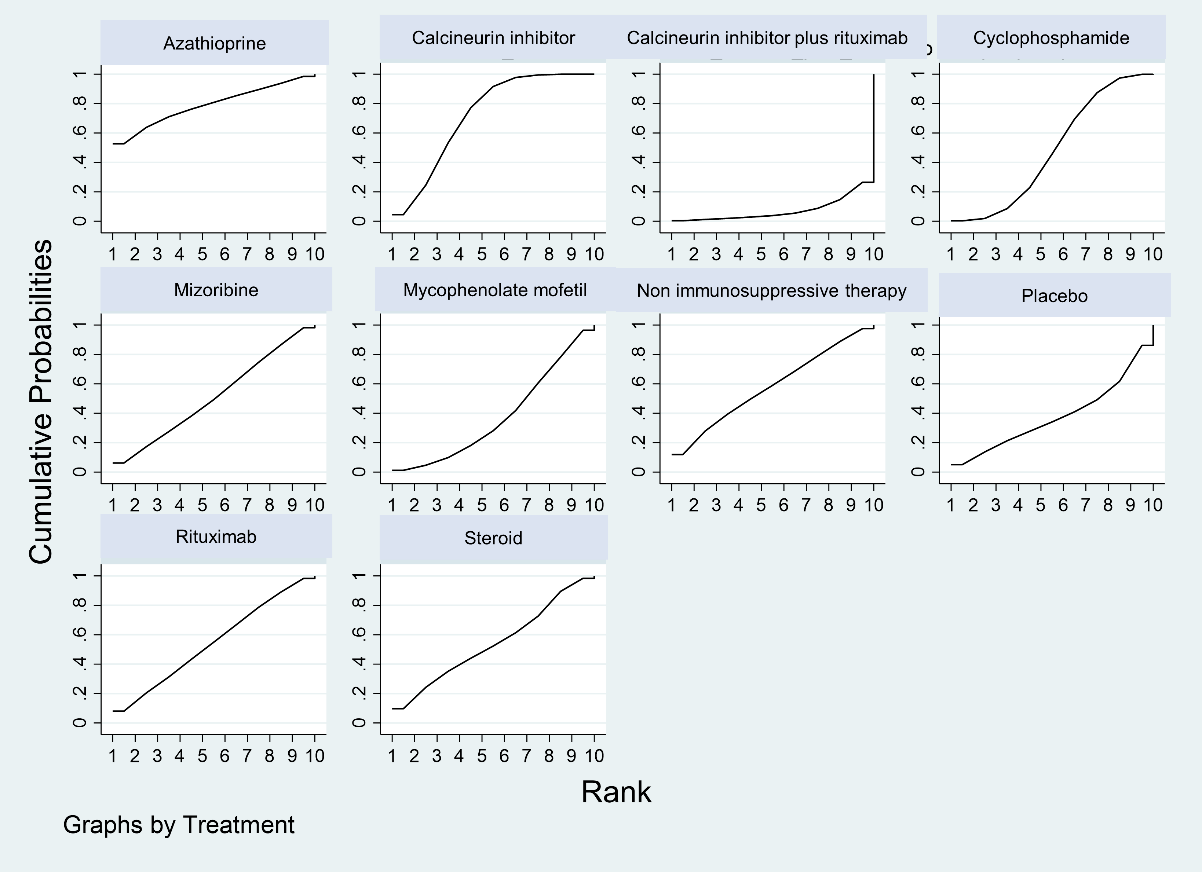
**

**Supplementary Figure 2.3. Partial remission**

**
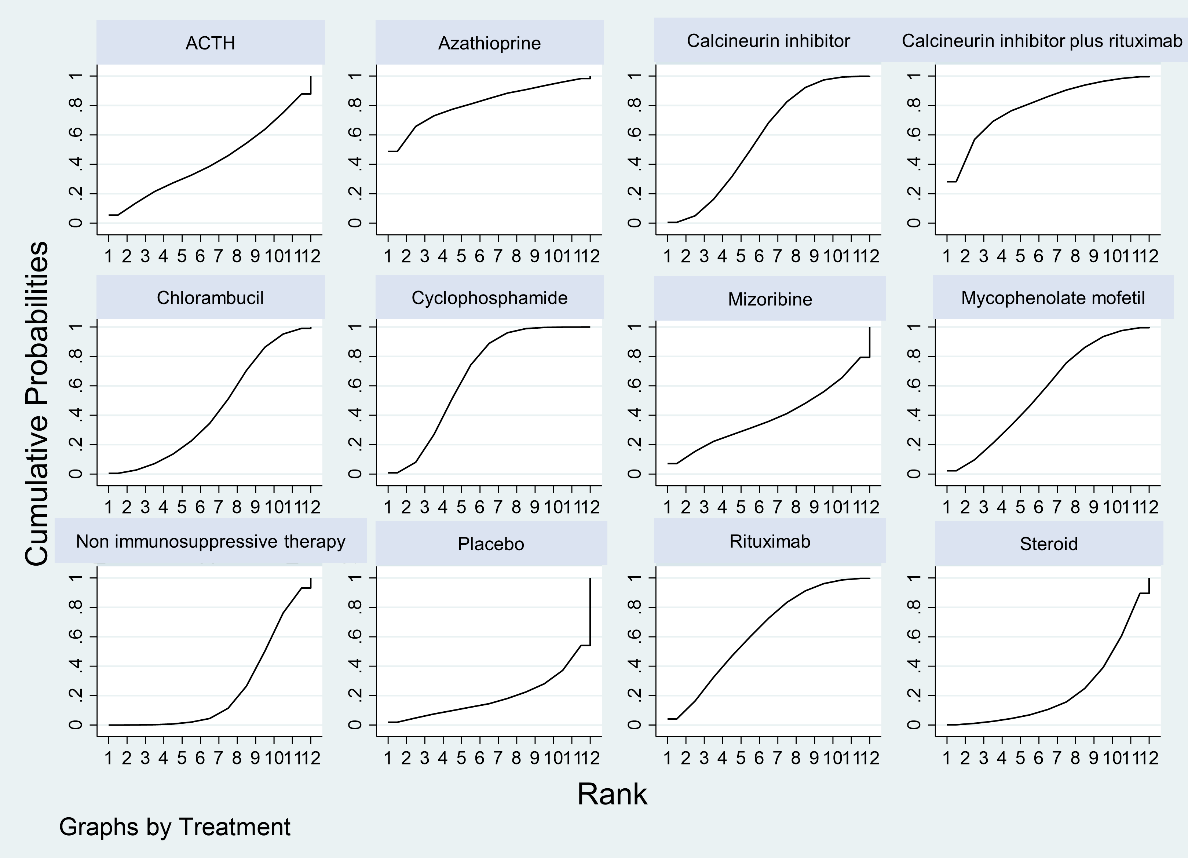
**

Abbreviations: ACTH = adrenocorticotropic hormone

**Supplementary Figure 2.4. Partial remission at 6 months**

**
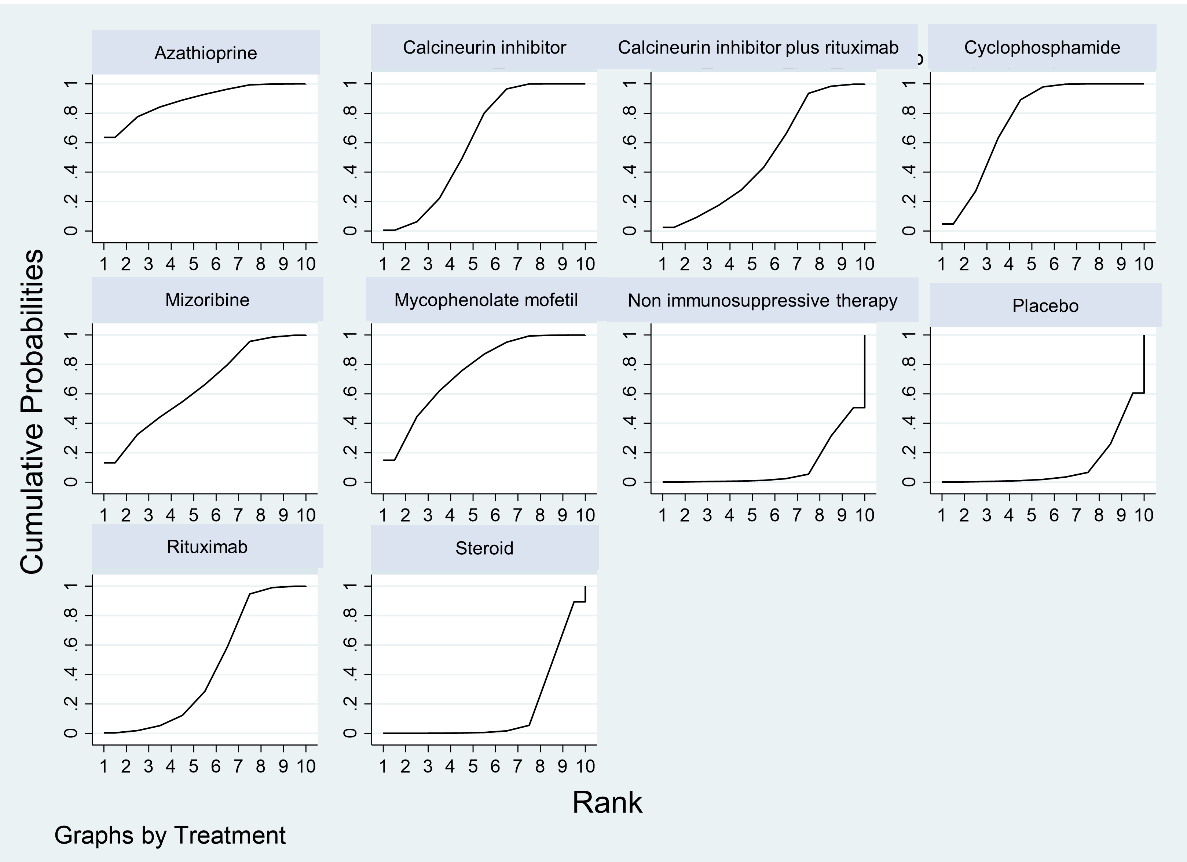
**

**Supplementary Figure 2.5. Kidney failure**

Abbreviations: ACTH = adrenocorticotropic hormone

**Supplementary Figure 2.6. Doubling of serum creatinine**

**Supplementary Figure 2.7. Glomerular Filtration Rate**

0

.

2

.

4

.

6

.

8

1

0

.

2

.

4

.

6

.

8

1

0

.

2

.

4

.

6

.

8

1

0

.

2

.

4

.

6

.

8

1

0

.

2

.

4

.

6

.

8

1

1

2

3

4

5

1

2

3

4

5

1

2

3

4

5

1

2

3

4

5

1

2

3

4

5

Azathioprine

Calcineurin inhibitor

Placebo

Non immunosuppressive therapy

Steroid

P

r

o

b

a

b

i

l

i

t

i

e

s

Rank

Graphs by Treatment

**Supplementary Figure 2.8. Proteinuria**

**Supplementary Figure 2.9. Serious Adverse Events**

**Supplementary Figure 2.10. Discontinuation of treatment**

**Supplementary Figure 2.11 Serious Infections**

**Supplementary Figure 2.12. Bone marrow suppression**

0

.2

.4

.6

.8

1

0

.2

.4

.6

.8

1

0

.2

.4

.6

.8

1

0

.2

.4

.6

.8

1

0

.2

.4

.6

.8

1

0

.2

.4

.6

.8

1

0

.2

.4

.6

.8

1

0

.2

.4

.6

.8

1

0

.2

.4

.6

.8

1

0

.2

.4

.6

.8

1

1

2

3

4

5

6

7

8

9

10

1

2

3

4

5

6

7

8

9

10

1

2

3

4

5

6

7

8

9

10

1

2

3

4

5

6

7

8

9

10

1

2

3

4

5

6

7

8

9

10

1

2

3

4

5

6

7

8

9

10

1

2

3

4

5

6

7

8

9

10

1

2

3

4

5

6

7

8

9

10

1

2

3

4

5

6

7

8

9

10

1

2

3

4

5

6

7

8

9

10

ACTH

Alkylating_agent

Azathioprine

Calcineurin inhibitor

Calcineurin inhibitor plus rituximab

Mycophenolate mofetil

Mizoribine

Placebo

Rituximab

Non immunosuppressive therapy

Probabilities

Rank

Graphs by Treatment

0

.2

.4

.6

.8

1

0

.2

.4

.6

.8

1

0

.2

.4

.6

.8

1

0

.2

.4

.6

.8

1

0

.2

.4

.6

.8

1

0

.2

.4

.6

.8

1

0

.2

.4

.6

.8

1

0

.2

.4

.6

.8

1

0

.2

.4

.6

.8

1

0

.2

.4

.6

.8

1

1

2

3

4

5

6

7

8

9

10

1

2

3

4

5

6

7

8

9

10

1

2

3

4

5

6

7

8

9

10

1

2

3

4

5

6

7

8

9

10

1

2

3

4

5

6

7

8

9

10

1

2

3

4

5

6

7

8

9

10

1

2

3

4

5

6

7

8

9

10

1

2

3

4

5

6

7

8

9

10

1

2

3

4

5

6

7

8

9

10

1

2

3

4

5

6

7

8

9

10

ACTH

Alkylating_agent

Azathioprine

Calcineurin inhibitor

Calcineurin inhibitor plus rituximab

Mycophenolate mofetil

Mizoribine

Placebo

Rituximab

Non immunosuppressive therapy

Probabilities

Rank

Graphs by Treatment

0

.2

.4

.6

.8

1

0

.2

.4

.6

.8

1

0

.2

.4

.6

.8

1

0

.2

.4

.6

.8

1

0

.2

.4

.6

.8

1

0

.2

.4

.6

.8

1

0

.2

.4

.6

.8

1

0

.2

.4

.6

.8

1

0

.2

.4

.6

.8

1

0

.2

.4

.6

.8

1

1

2

3

4

5

6

7

8

9

10

1

2

3

4

5

6

7

8

9

10

1

2

3

4

5

6

7

8

9

10

1

2

3

4

5

6

7

8

9

10

1

2

3

4

5

6

7

8

9

10

1

2

3

4

5

6

7

8

9

10

1

2

3

4

5

6

7

8

9

10

1

2

3

4

5

6

7

8

9

10

1

2

3

4

5

6

7

8

9

10

1

2

3

4

5

6

7

8

9

10

ACTH

Alkylating_agent

Azathioprine

Calcineurin inhibitor

Calcineurin inhibitor plus rituximab

Mycophenolate mofetil

Mizoribine

Placebo

Rituximab

Non immunosuppressive therapy

Probabilities

Rank

Graphs by Treatment

0

.2

.4

.6

.8

1

0

.2

.4

.6

.8

1

0

.2

.4

.6

.8

1

0

.2

.4

.6

.8

1

0

.2

.4

.6

.8

1

0

.2

.4

.6

.8

1

0

.2

.4

.6

.8

1

0

.2

.4

.6

.8

1

0

.2

.4

.6

.8

1

0

.2

.4

.6

.8

1

1

2

3

4

5

6

7

8

9

10

1

2

3

4

5

6

7

8

9

10

1

2

3

4

5

6

7

8

9

10

1

2

3

4

5

6

7

8

9

10

1

2

3

4

5

6

7

8

9

10

1

2

3

4

5

6

7

8

9

10

1

2

3

4

5

6

7

8

9

10

1

2

3

4

5

6

7

8

9

10

1

2

3

4

5

6

7

8

9

10

1

2

3

4

5

6

7

8

9

10

ACTH

Alkylating_agent

Azathioprine

Calcineurin inhibitor

Calcineurin inhibitor plus rituximab

Mycophenolate mofetil

Mizoribine

Placebo

Rituximab

Non immunosuppressive therapy

Probabilities

Rank

Graphs by Treatment

**Supplementary Figure 3: Comparison adjusted funnel plot**

**Supplementary Figure 3.1. Complete remission**

Abbreviations: A = Cyclophosphamide; B = Chlorambucil; C = Calcineurin inhibitor; D = Rituximab; E = Calcineurin inhibitor plus rituximab; F = Mycophenolate mofetil; G = Mizoribine; H = Azathioprine; I = Adrenocorticotrophic hormone; J = Leflunomide; K = Steroids; L = Placebo; M = Non immunosuppressive therapy

**Supplementary Figure 3.2. Complete remission at 6 months**

Abbreviations: A = Cyclophosphamide; B = Calcineurin inhibitor; C = Rituximab; D = Calcineurin inhibitor plus rituximab; E = Mycophenolate mofetil; F = Mizoribine; G = Azathioprine; H = Steroid; I = Placebo; J = Non immunosuppressive therapy

**Supplementary Figure 3.3. Partial Remission**

Abbreviations: A = Cyclophosphamide; B = Chlorambucil; C = Calcineurin inhibitor; D = Rituximab; E = Calcineurin inhibitor plus rituximab; F = Mycophenolate mofetil; G = Mizoribine; H = Azathioprine; I = Adrenocorticotrophic hormone; J = Steroids; K = Placebo; L = Non immunosuppressive therapy

**Supplementary Figure 3.4. Partial remission at 6 months**

Abbreviations: A = Cyclophosphamide; B = Calcineurin inhibitor; C = Rituximab; D = Calcineurin inhibitor plus rituximab; E = Mycophenolate mofetil; F = Mizoribine; G = Azathioprine; H = Steroid; I = Placebo; J = Non immunosuppressive therapy

**Supplementary Figure 3.5. Kidney Failure**

Abbreviations: A = Cyclophosphamide; B = Chlorambucil; C = Calcineurin inhibitor; D = Rituximab; E = Calcineurin inhibitor plus rituximab; F = Mycophenolate mofetil; G = Adrenocorticotrophic hormone; H = Steroid; I = Placebo; J = Non immunosuppressive therapy

**Supplementary Figure 3.6. Doubling of Creatinine**

Abbreviations: A = Cyclophosphamide; B = Chlorambucil; C = Calcineurin inhibitor; D = Mycophenolate; E = Steroid; F = Placebo; G = Non-immunosuppressive therapy

**Supplementary Figure 3.7. Glomerular Filtration Rate**

Abbreviations: A = Calcineurin inhibitor; B = Steroid; C = Azathioprine; D = Non immunosuppressive therapy; E = Placebo

**Supplementary Figure 3.8. Proteinuria**

Abbreviations: A = Cyclophosphamide agent; B = Chlorambucil; C = Calcineurin inhibitor; D = Mycophenolate; E = Mizoribine; F = Azathioprine; G = Steroid; H = Placebo; I = Non immunosuppressive therapy

**Supplementary Figure 3.9. Serious Adverse Events**

Abbreviations: A = Cyclophosphamide; B = Chlorambucil; C = Calcineurin inhibitor; D = Rituximab; E = Calcineurin inhibitor plus rituximab; F = Mycophenolate mofetil; G = Mizoribine; H = Steroids; I = Placebo; J = Non immunosuppressive therapy

**Supplementary Figure 3.10. Discontinuation of Treatment**

Abbreviations: A = Cyclophosphamide; B = Chlorambucil; C = Calcineurin inhibitor; D = Rituximab; E = Mycophenolate; F = Azathioprine; G = Steroid; H = Placebo; I = Non immunosuppressive therapy

**Supplementary Figure 3.11. Serious Infection**

Abbreviations: A = Cyclophosphamide; B = Chlorambucil; C = Calcineurin inhibitor; D = Rituximab; D = Steroid; E = Calcineurin inhibitor plus rituximab; **F = Mycophenolate mofetil; G = Mizoribine; H = Azathioprine; I = Steroid; J = Placebo; K = Non immunosuppressive therapy**

**Supplementary Figure 3.12. Bone marrow Suppression**

Abbreviations: A = Cyclophosphamide; B = Chlorambucil; C = Calcineurin inhibitor; D = Rituximab; E = Calcineurin inhibitor plus rituximab; F = Mycophenolate mofetil; G = Mizoribine; H = Azathioprine; I = Adrenocorticotrophic hormone; J = placebo; K = Non immunosuppressive therapy

**Supplementary Figure 4.1 Complete remission paired meta-analysis versus cyclophosphamide**

**
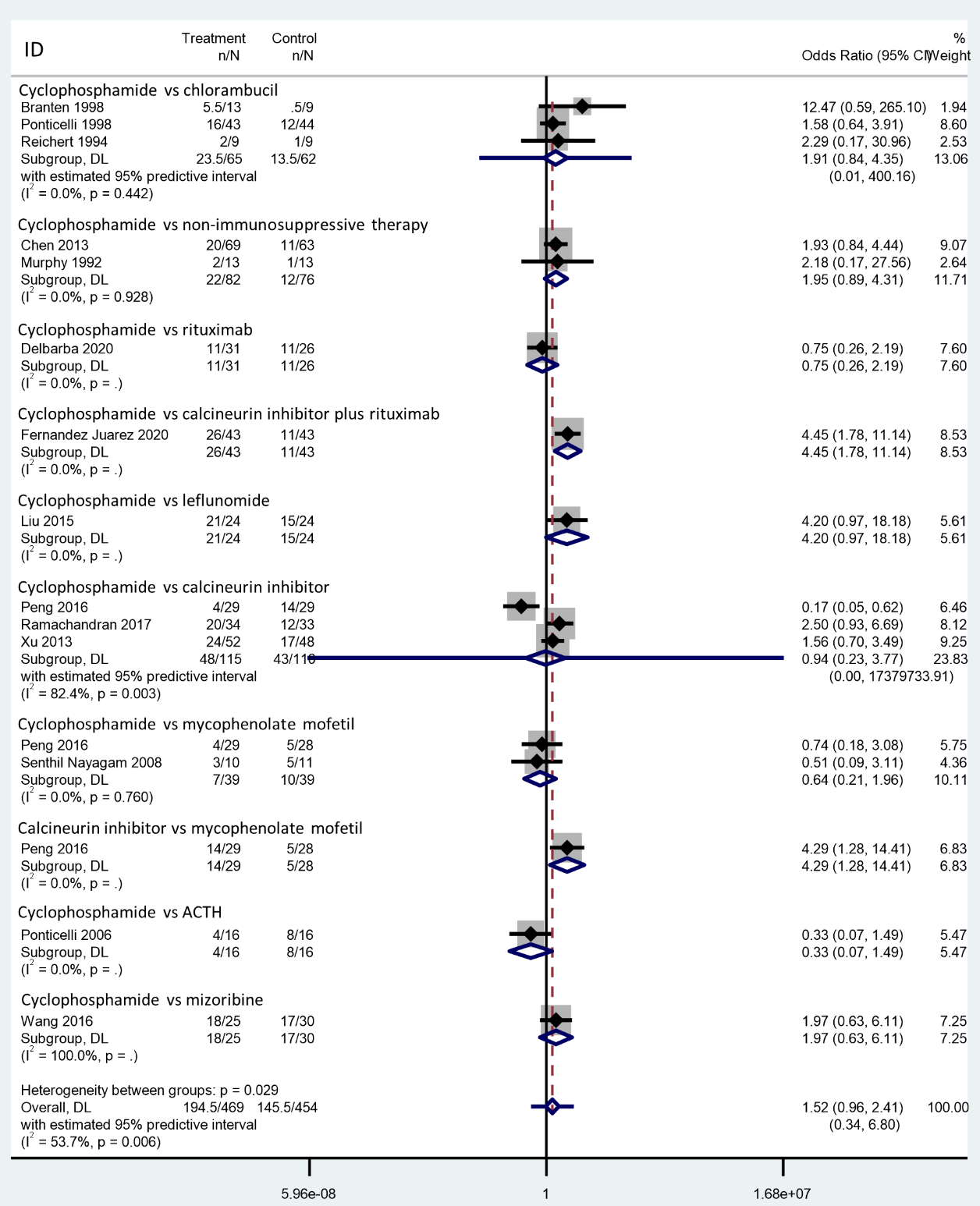
**

Abbreviations: ACTH = adrenocorticotropic hormone

**Supplementary Figure 4.2. Complete remission at 6 months paired meta-analysis versus cyclophosphamide**

**
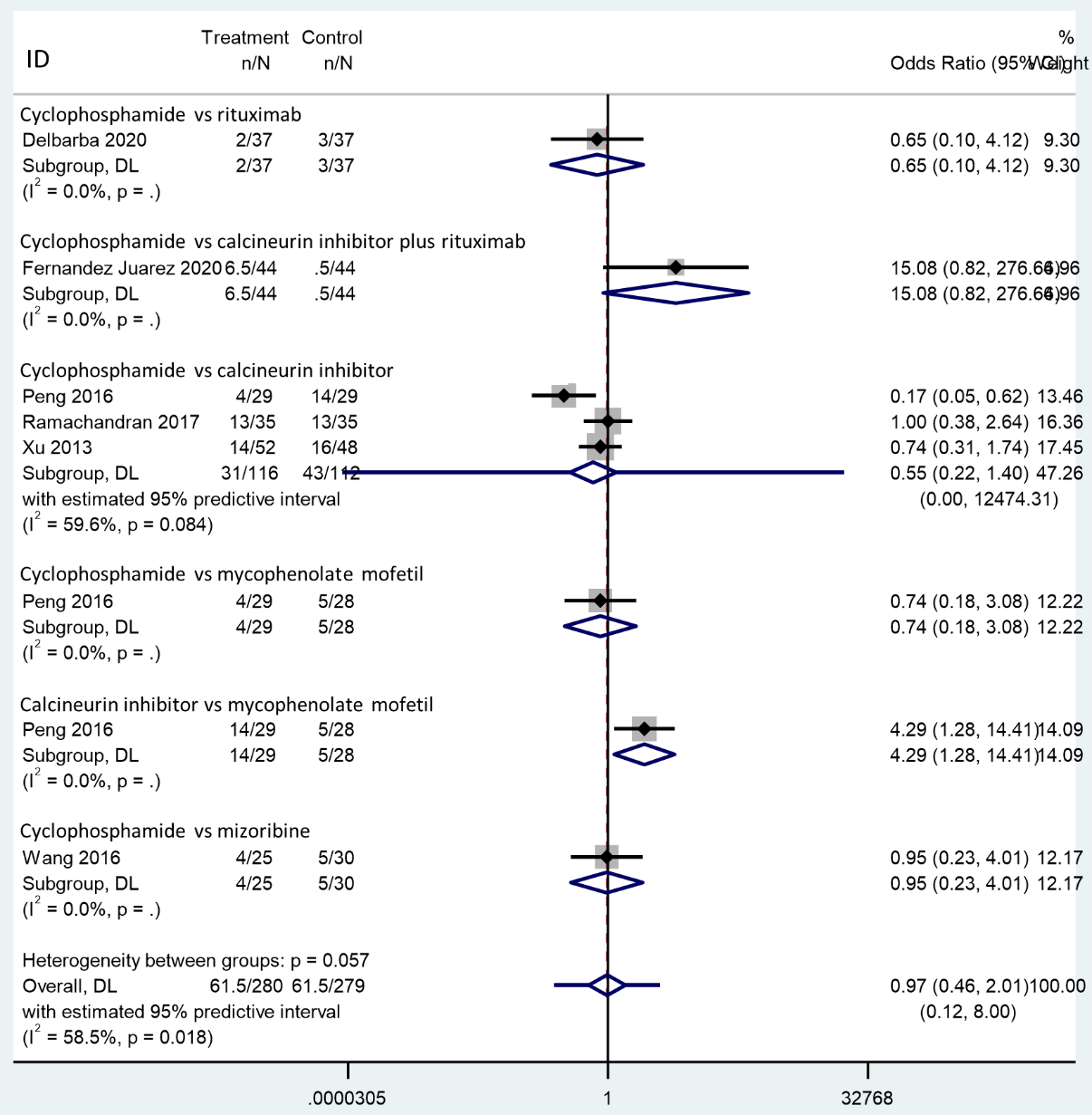
**

**Supplementary Figure 4.3. Partial remission paired meta-analysis versus cyclophosphamide**

**
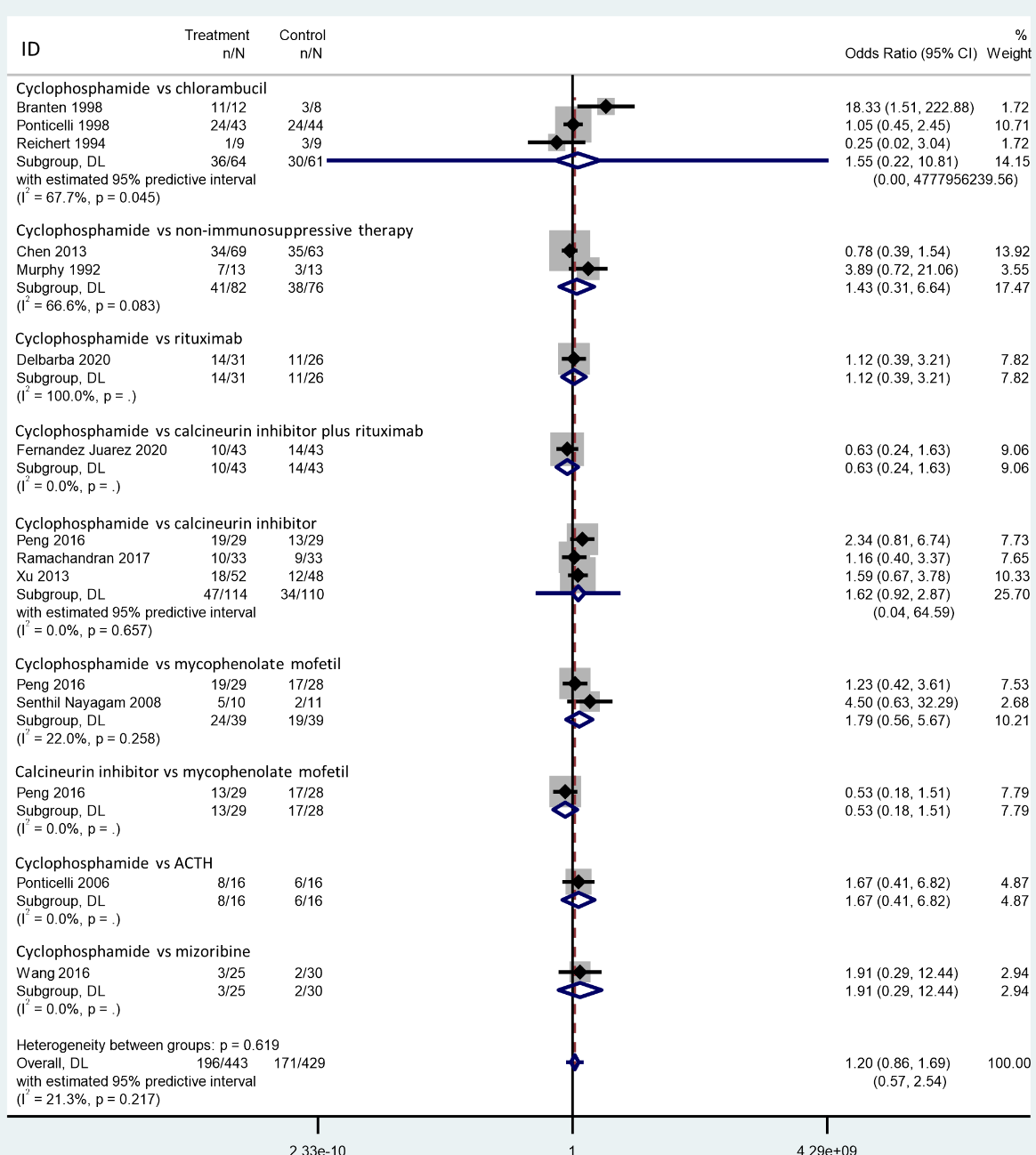
**

Abbreviations: ACTH = adrenocorticotropic hormone

**Supplementary Figure 4.4. Partial remission at 6 months paired meta-analysis versus cyclophosphamide**

**
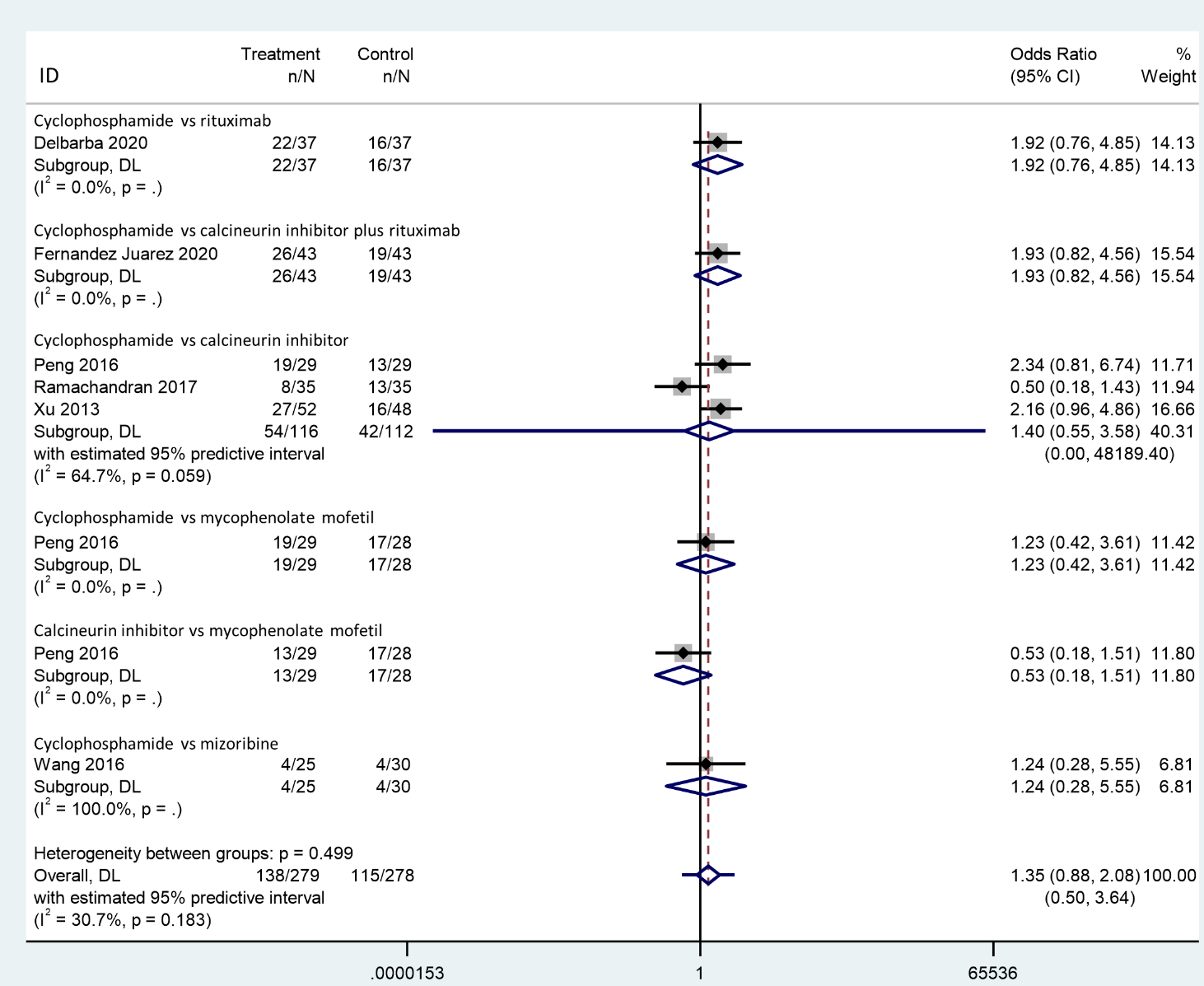
**

**Supplementary Figure 4.5 Kidney failure paired meta-analysis versus cyclophosphamide**

**
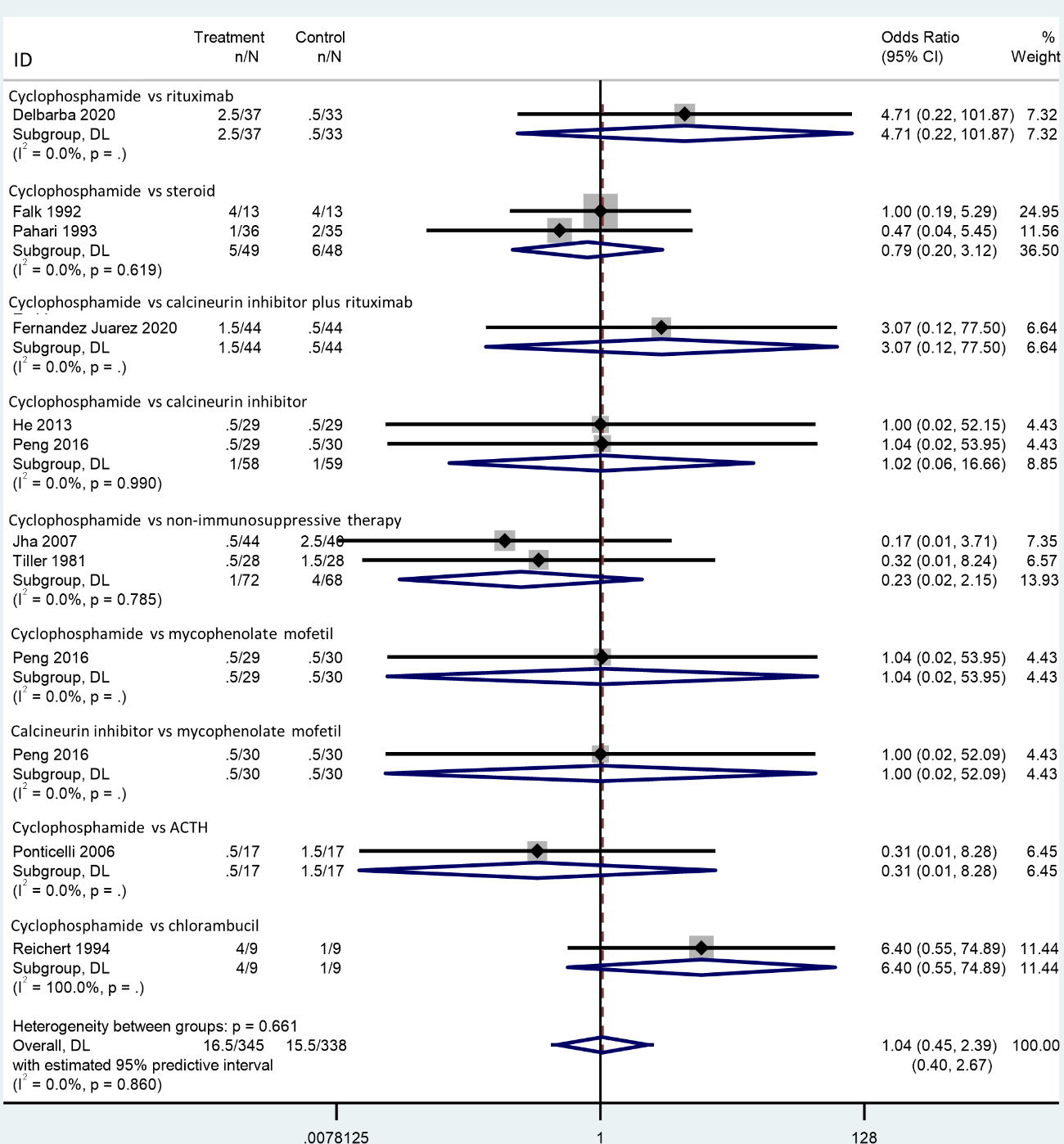
**

Abbreviations: ACTH = adrenocorticotropic hormone;

**Supplementary Figure 4.6 Doubling of serum creatinine paired meta-analysis versus cyclophosphamide**

**
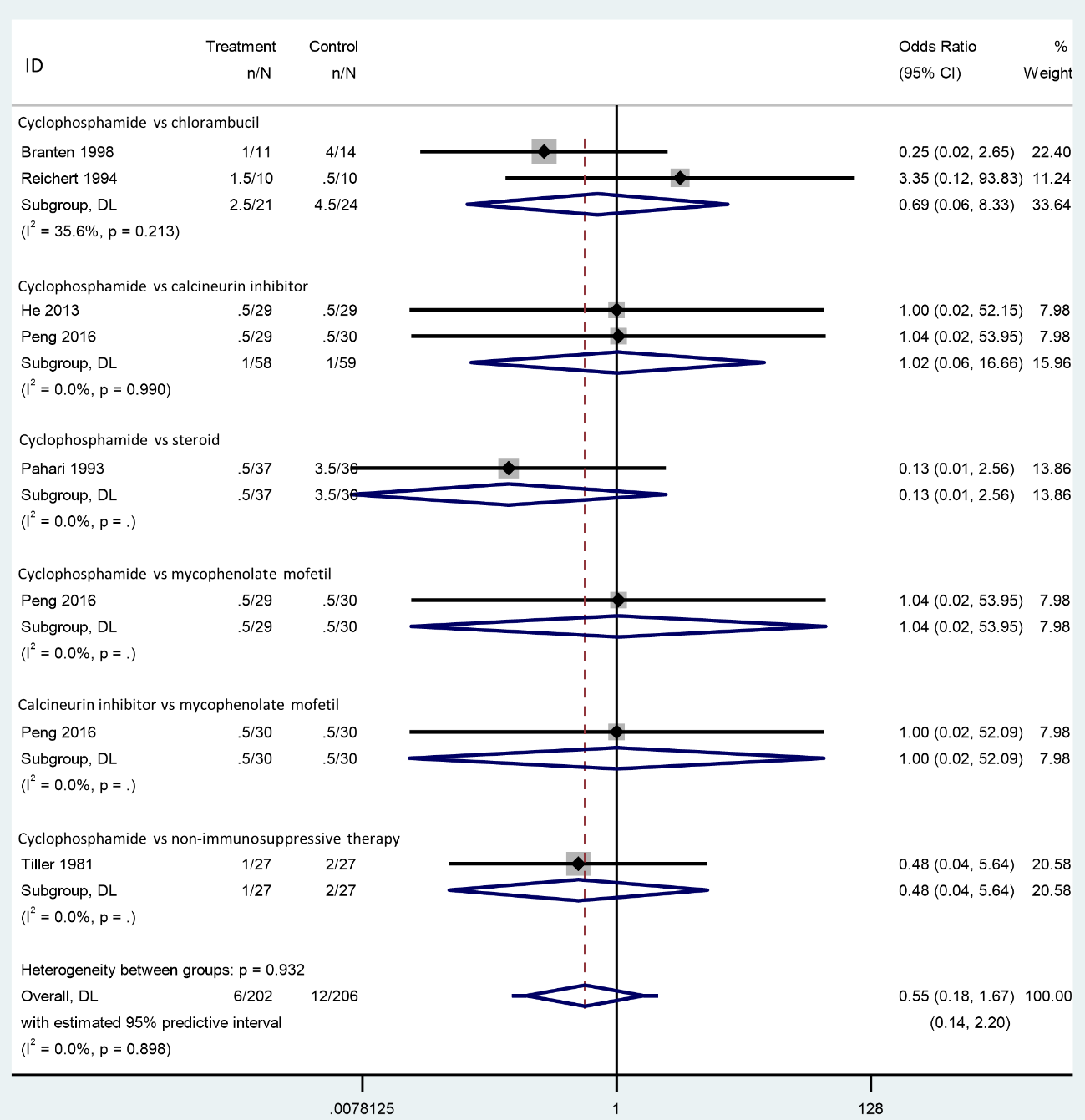
**

**Supplementary Figure 4.7 Proteinuria paired meta-analysis versus cyclophosphamide**

**
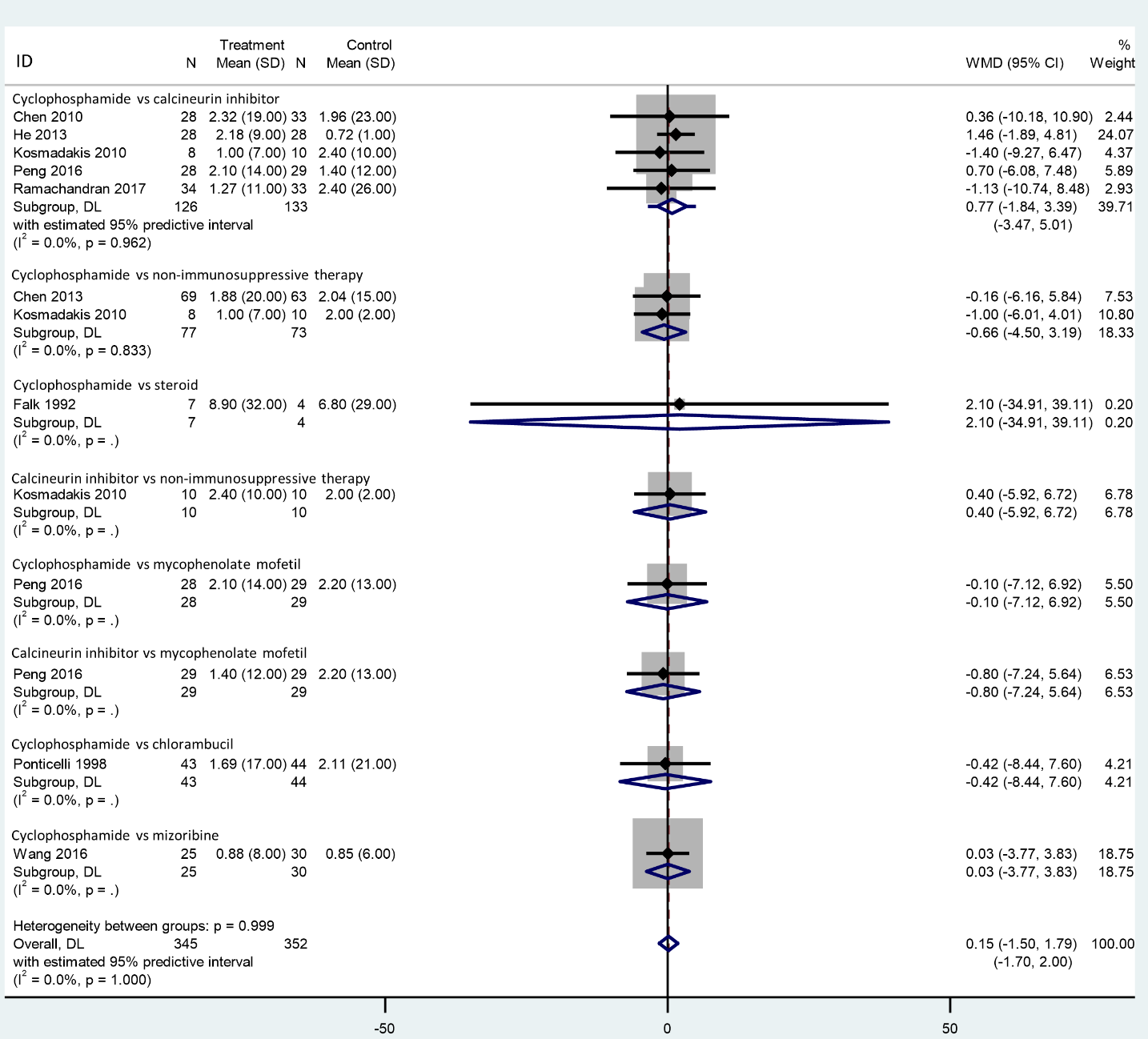
**

**Supplementary Figure 4.8 Discontinuation of treatment paired meta-analysis versus cyclophosphamide**

**
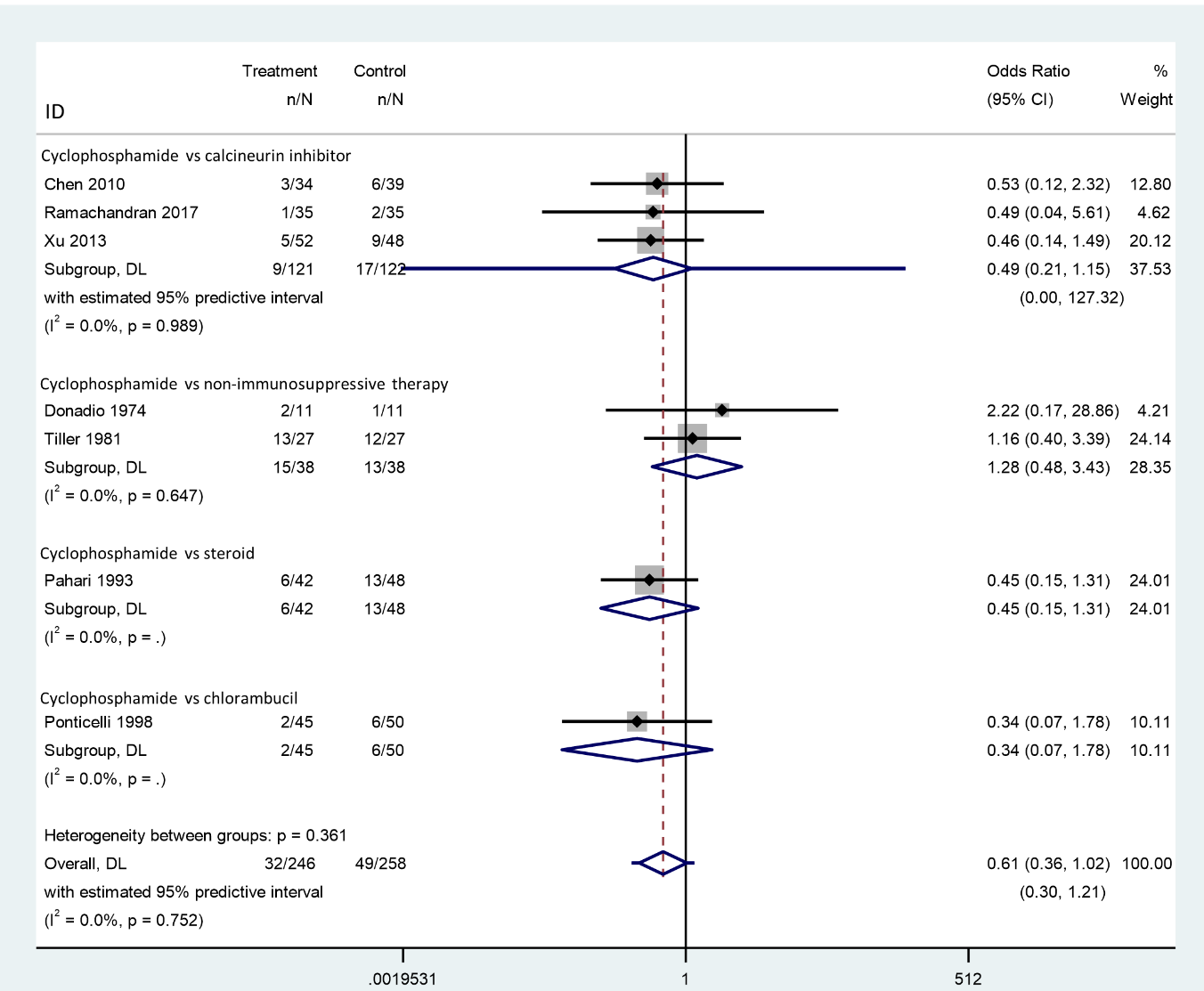
**

**Supplementary Figure 4.9 Serious infection paired meta-analysis versus cyclophosphamide**

**
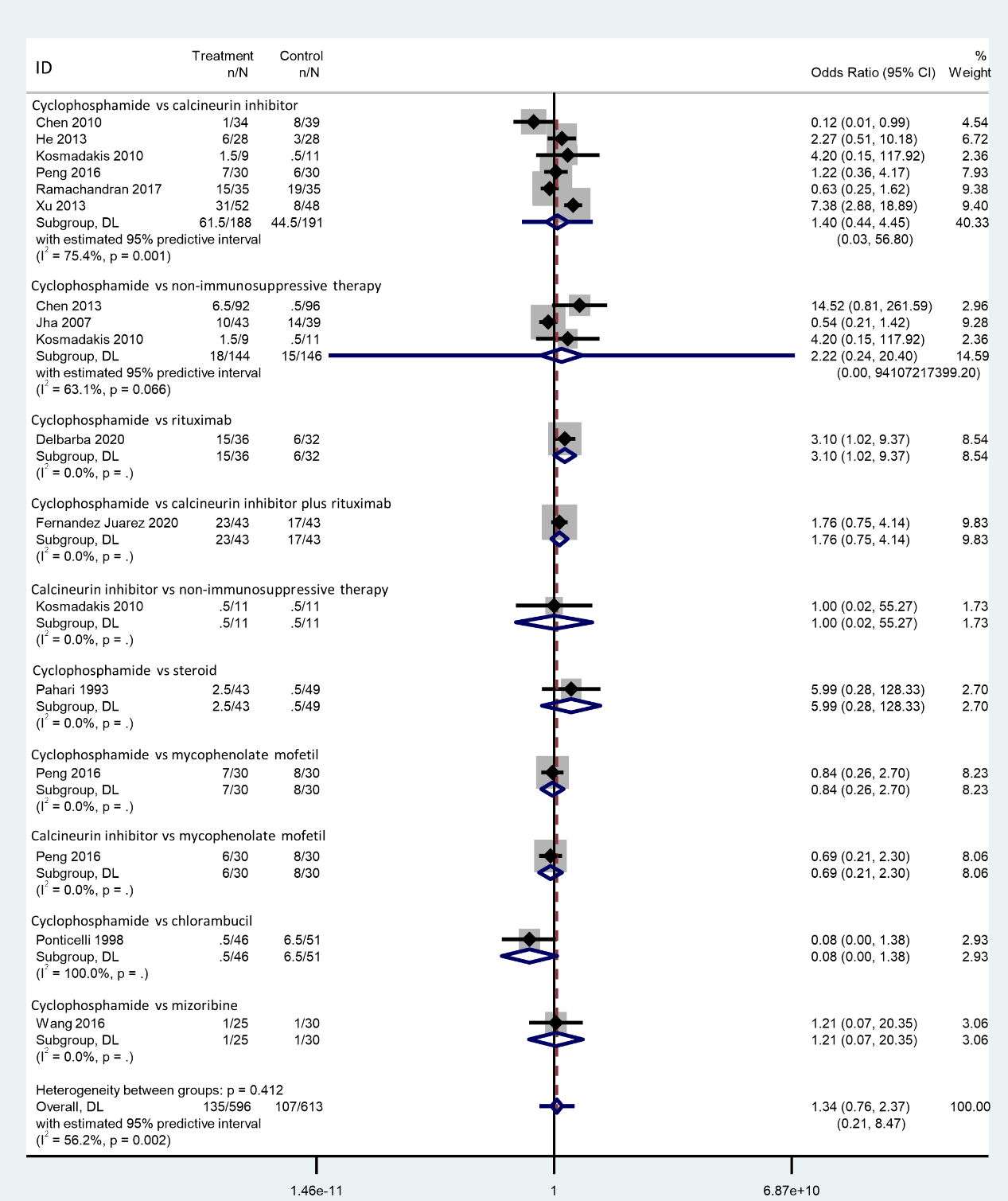
**

**Supplementary Figure 4.10 Bone marrow suppression paired meta-analysis versus cyclophosphamide**

**
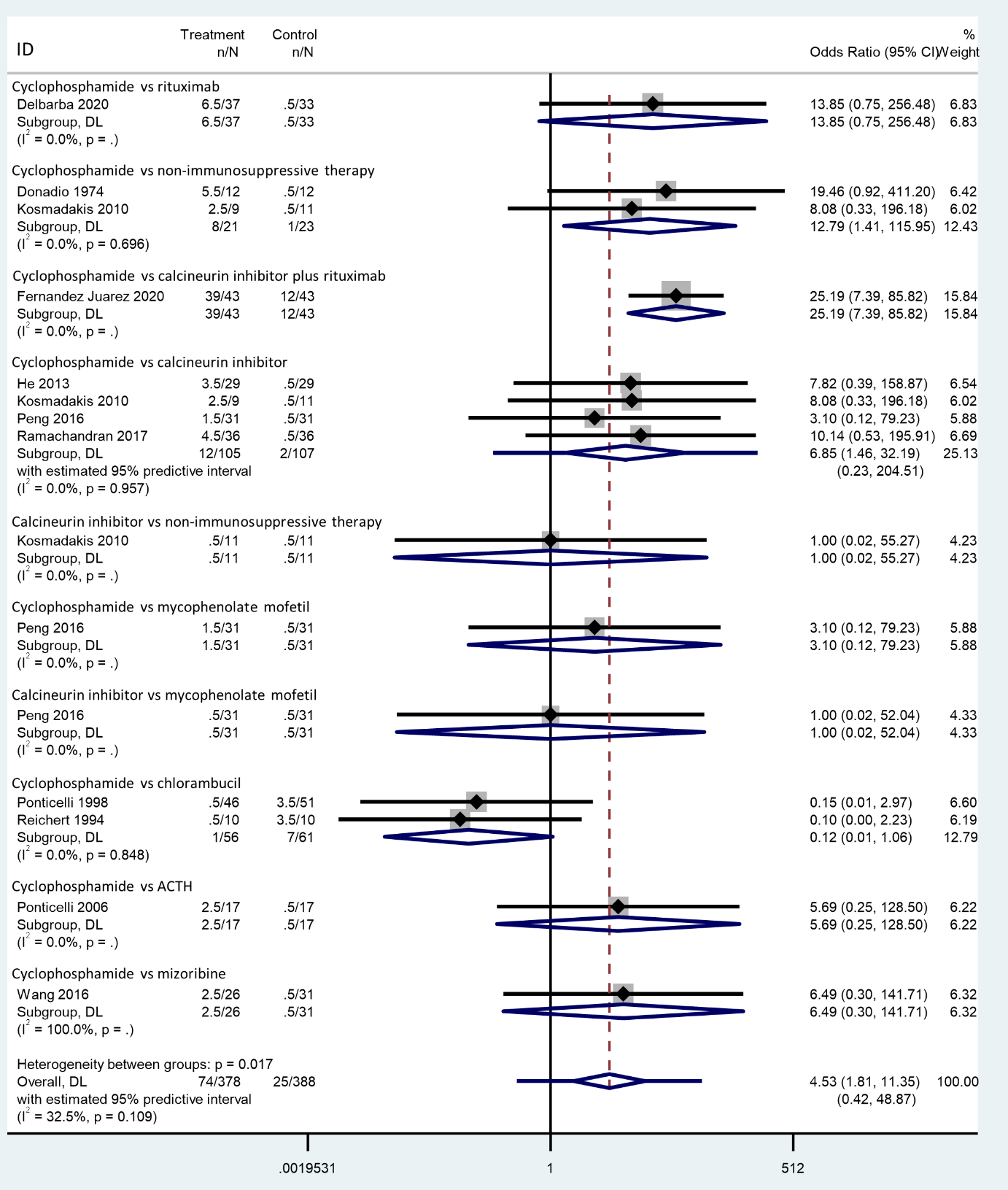
**

Abbreviations: ACTH = adrenocorticotropic hormone

**References:**

1. Caldwell DM, Ades AE, Higgins JP. Simultaneous comparison of multiple treatments: combining direct and indirect evidence. *BMJ.* 2005;331(7521):897-900.

2. Lu G, Ades AE. Combination of direct and indirect evidence in mixed treatment comparisons. *Stat Med.* 2004;23(20):3105-3124.

3. Salanti G, Higgins JP, Ades AE, Ioannidis JP. Evaluation of networks of randomized trials. *Stat Methods Med Res.* 2008;17(3):279-301.

4. Higgins JP, Jackson D, Barrett JK, Lu G, Ades AE, White IR. Consistency and inconsistency in network meta-analysis: concepts and models for multi-arm studies. *Res Synth Methods.* 2012;3(2):98-110.

5. Chaimani A, Higgins JP, Mavridis D, Spyridonos P, Salanti G. Graphical tools for network meta-analysis in STATA. *PLoS One.* 2013;8(10):e76654.

6. White I. Multivariate random-effects meta-regression: Updates to mvmeta. *The Strata Journal.* 2011;11(2):255-270.

7. White I. Multivariate random-effects meta-analysis. *The Strata Journal.* 2009;9(1):40-56.

8. Salanti G, Ades AE, Ioannidis JP. Graphical methods and numerical summaries for presenting results from multiple-treatment meta-analysis: an overview and tutorial. *J Clin Epidemiol.* 2011;64(2):163-171.

9. Spiegelhalter DJ AK, Myles J. *Beyesian approaches to clinical trials and health-care evaluation.* John Wiley and Sons Ltd; 2004.

10. Bucher HC, Guyatt GH, Griffith LE, Walter SD. The results of direct and indirect treatment comparisons in meta-analysis of randomized controlled trials. *J Clin Epidemiol.* 1997;50(6):683-691.

11. Veroniki AA, Vasiliadis HS, Higgins JP, Salanti G. Evaluation of inconsistency in networks of interventions. *Int J Epidemiol.* 2013;42(1):332-345.

12. Hutton B, Salanti G, Caldwell DM, et al. The PRISMA extension statement for reporting of systematic reviews incorporating network meta-analyses of health care interventions: checklist and explanations. *Ann Intern Med.* 2015;162(11):777-784.

13. Brignardello-Petersen R, Bonner A, Alexander PE, et al. Advances in the GRADE approach to rate the certainty in estimates from a network meta-analysis. *J Clin Epidemiol.* 2018;93:36-44.

14. Controlled trial of azathioprine and prednisone in chronic renal disease. Report by Medical Research Council Working Party. *British medical journal.* 1971;2(5756):239-241.

15. Silverberg DS, Atkins EL, Ballon HC. Controlled trial of azathioprine in the nephrotic syndrome secondary to idiopathic membranous glomerulonephritis. *Canadian Medical Association Journal.* 1976;115(12):1209-1210.

16. Coggins CH. A controlled study of short-term prednisone treatment in adults with membranous nephropathy. Collaborative study of the adult idiopathic nephrotic syndrome. *N Engl J Med.* 1979;301(24):1301-1306.

17. Badri S, Dashti-Khavidaki S, Ahmadi F, Mahdavi-Mazdeh M, Abbasi MR, Khalili H. Effect of add-on pentoxifylline on proteinuria in membranous glomerulonephritis: A 6-month placebo-controlled trial. *Clinical Drug Investigation.* 2013;33(3):215-222.

18. Cattran DC, Appel GB, Hebert LA, et al. Cyclosporine in patients with steroid-resistant membranous nephropathy: a randomized trial. *Kidney International.* 2001;59(4):1484-1490.

19. Xu J, Zhang W, Xu Y, et al. Tacrolimus combined with corticosteroids in idiopathic membranous nephropathy: a randomized, prospective, controlled trial. *Contrib Nephrol.* 2013;181:152-162.

20. Howman A, Chapman TL, Langdon MM, et al. Immunosuppression for progressive membranous nephropathy: A UK randomised controlled trial. *The Lancet.* 2013;381(9868):744-751.

21. Naumovic R, Jovanovic D, Pavlovic S, Stosovic M, Marinkovic J, Basta-Jovanovic G. Cyclosporine versus azathioprine therapy in high-risk idiopathic membranous nephropathy patients: A 3-year prospective study. *Biomed Pharmacother.* 2011;65(2):105-110.

22. Kosmadakis G, Filiopoulos V, Smirloglou D, Skarlas P, Georgoulias C, Michail S. Comparison of immunosuppressive therapeutic regimens in patients with nephrotic syndrome due to idiopathic membranous nephropathy. *Ren Fail.* 2010;32(5):566-571.

23. Chen M, Li H, Li XY, et al. Tacrolimus combined with corticosteroids in treatment of nephrotic idiopathic membranous nephropathy: a multicenter randomized controlled trial. *Am J Med Sci.* 2010;339(3):233-238.

24. Senthil Nayagam L, Ganguli A, Rathi M, et al. Mycophenolate mofetil or standard therapy for membranous nephropathy and focal segmental glomerulosclerosis: a pilot study. *Nephrology Dialysis Transplantation.* 2008;23(6):1926-1930.

25. Dussol B, Morange S, Burtey S, et al. Mycophenolate mofetil monotherapy in membranous nephropathy: a 1-year randomized controlled trial. *American Journal of Kidney Diseases.* 2008;52(4):699-705.

26. Praga M, Barrio V, Juarez GF, Luno J, Grupo Espanol de Estudio de la Nefropatia M. Tacrolimus monotherapy in membranous nephropathy: a randomized controlled trial. *Kidney International.* 2007;71(9):924-930.

27. Jha V, Ganguli A, Saha TK, et al. A randomized, controlled trial of steroids and cyclophosphamide in adults with nephrotic syndrome caused by idiopathic membranous nephropathy. *J Am Soc Nephrol.* 2007;18(6):1899-1904.

28. Chan TM, Lin AW, Tang SC, et al. Prospective controlled study on mycophenolate mofetil and prednisolone in the treatment of membranous nephropathy with nephrotic syndrome. *Nephrology.* 2007;12(6):576-581.

29. Ponticelli C, Altieri P, Scolari F, et al. A randomized study comparing methylprednisolone plus chlorambucil versus methylprednisolone plus cyclophosphamide in idiopathic membranous nephropathy. *J Am Soc Nephrol.* 1998;9(3):444-450.

30. Branten AJ, Reichert LJ, Koene RA, Wetzels JF. Oral cyclophosphamide versus chlorambucil in the treatment of patients with membranous nephropathy and renal insufficiency. *Qjm.* 1998;91(5):359-366.

31. Austin HA VE, Boumpas DT, Balow JE. Randomized Trial of pulse cyclophosphamide and prednisolone vs. prednisolone alone in idiopathic membranous nephropathy. [abstract no: A0410]. *Journal of the AMerican Society of Nephrology: JASN.* 1996;7:1327.

32. Ponticelli C, Zucchelli P, Passerini P, et al. A 10-year follow-up of a randomized study with methylprednisolone and chlorambucil in membranous nephropathy. *Kidney international.* 1995;48(5):1600-1604.

33. Cattran DC, Greenwood C, Ritchie S, et al. A controlled trial of cyclosporine in patients with progressive membranous nephropathy. Canadian Glomerulonephritis Study Group. *Kidney International.* 1995;47(4):1130-1135.

34. Reichert LJ, Huysmans FT, Assmann K, Koene RA, Wetzels JF. Preserving renal function in patients with membranous nephropathy: daily oral chlorambucil compared with intermittent monthly pulses of cyclophosphamide. *Annals of Internal Medicine.* 1994;121(5):328-333.

35. Falk RJ, Hogan SL, Muller KE, Jennette JC. Treatment of progressive membranous glomerulopathy. A randomized trial comparing cyclophosphamide and corticosteroids with corticosteroids alone. The Glomerular Disease Collaborative Network. *Annals of Internal Medicine.* 1992;116(6):438-445.

36. Cameron JS, Healy MJR, Adu D. The Medical Research Council Trial of short-term high-dose alternate day prednisolone in idiopathic membranous nephropathy with nephrotic syndrome in adults. *Quarterly Journal of Medicine.* 1990;74(274):133-156.

37. Cattran DC, Delmore T, Roscoe J, et al. A randomized controlled trial of prednisone in patients with idiopathic membranous nephropathy. *N Engl J Med.* 1989;320(4):210-215.

38. Donadio JV, Jr., Holley KE, Anderson CF, Taylor WF. Controlled trial of cyclophosphamide in idiopathic membranous nephropathy. *Kidney International.* 1974;6(6):431-439.

39. Murphy BF, McDonald I, Fairley KF, Kincaid-Smith PS. Randomized controlled trial of cyclophosphamide, warfarin and dipyridamole in idiopathic membranous glomerulonephritis. *Clinical Nephrology.* 1992;37(5):229-234.

40. Ahmed S, Rahman M, Alam MR, et al. Methyl prednisolone plus chlorambucil as compared with prednisolone alone for the treatment of idiopathic membranous nephropathy - A preliminary study. *Bangladesh Renal Journal.* 1994;13(2):51-54.

41. Pahari DK, Das S, Dutta BN, Banerjee D. Prognosis and management of membraneous nephropathy. *J Assoc Physicians India.* 1993;41(6):350-351.

42. Tiller DJ CA, Mathew T, Thompson N, Row G, Lauer C et al. A prospective randomised trial of cyclophosphamide, dipyridamole and warfarin in membranous and mesangiocapillary glomerulonephritis. Paper presented at: Advanced in Basic and Clinical Nephrology 8th International Congress of Nephrology1981.

43. Kibriya MG, Tishkov I, Nikolov D. Immunosuppressive therapy with cyclophosphamide and prednisolone in severe idiopathic membranous nephropathy. *Nephrology Dialysis Transplantation.* 1994;9(2):138-143.

44. Peng L, Wei SY, Li LT, He YX, Li B. Comparison of different therapies in high-risk patients with idiopathic membranous nephropathy. *J Formos Med Assoc.* 2016;115(1):11-18.

45. Ramachandran R, Kumar V, Nada R, Kohli HS, Jha V, Gupta KL. Tacrolimus with corticosteroids is inferior to modified ponticelli regimen in the management of primary membranous nephropathy: Results at 2 years of randomization. *Nephrology Dialysis Transplantation.* 2016;1):i77.

46. Liu S. Clinical trial of treatment for idiopathic membranous nephropathy with leflunomide combined with cyclophosphamide and glucocorticoid. *Hong Kong Journal of Nephrology.* 2015;1):S64.

47. Omrani H, Golmohamadi S, Hichi F, Sadeghi M. Comparison of the efficacy of tacrolimus versus cyclosporine in the treatment of idiopathic membranous nephropathy. *Nephro-Urology Monthly.* 2017;9 (1) (no pagination)(e42473).

48. Yuan H, Liu N, Sun GD, Jia Y, Luo P, Miao LN. Effect of prolonged tacrolimus treatment in idiopathic membranous nephropathy with nephrotic syndrome. *Pharmacology.* 2013;91(5-6):259-266.

49. Sun Z, Ren M, Wu Q, Du X. Co-administration of Wuzhi capsules and tacrolimus in patients with idiopathic membranous nephropathy: clinical efficacy and pharmacoeconomics. *Int Urol Nephrol.* 2014;46(10):1977-1982.

50. Dede F, Ayli D, Sahiner S. Effective treatment administration of cyclophosphamide in membranous nephropathy. *Journal of Nephrology.* 2008;21(4):560-565.

51. Gopal KA, Sahay M, Raman A, Narayen G. Ponticelli regime for membranous nephropathy - do Indians respond differently? *Nephrology dialysis transplantation.* 2003;18(Suppl 4):615.

52. Hasegawa H, Mitarai T, Tomino Y, et al. Clinical advantage of concomitant use of mizoribine and prednisolone on primary membranous nephropathy in the elderly. *Nephrology Dialysis Transplantation.* 2017;32 (Supplement 3):iii509.

53. He L, Peng Y, Liu H, et al. Treatment of idiopathic membranous nephropathy with combination of low-dose tacrolimus and corticosteroids. *Journal of Nephrology.* 2013;26(3):564-571.

54. Hofstra JM, Branten AJW, Wirtz JJJM, Noordzij TC, Du Buf-Vereijken PWG, Wetzels JFM. Early versus late start of immunosuppressive therapy in idiopathic membranous nephropathy: A randomized controlled trial. *Nephrology Dialysis Transplantation.* 2010;25(1):129-136.

55. Saito T, Iwano M, Matsumoto K, et al. Significance of combined cyclosporine-prednisolone therapy and cyclosporine blood concentration monitoring for idiopathic membranous nephropathy with steroid-resistant nephrotic syndrome: a randomized controlled multicenter trial. *Clin Exp Nephrol.* 2014;18(5):784-794.

56. Dahan K, Debiec H, Plaisier E, et al. Rituximab for Severe Membranous Nephropathy: A 6-Month Trial with Extended Follow-Up. *J Am Soc Nephrol.* 2017;28(1):348-358.

57. Fervenza FC, Appel GB, Barbour SJ, et al. Rituximab or Cyclosporine in the Treatment of Membranous Nephropathy. *N Engl J Med.* 2019;381(1):36-46.

58. Chen Y, Deng Y, Ni Z, et al. Efficacy and safety of traditional chinese medicine (Shenqi particle) for patients with idiopathic membranous nephropathy: a multicenter randomized controlled clinical trial. *American Journal of Kidney Diseases.* 2013;62(6):1068-1076.

59. Dussol B, Sichez H, Burtey S, et al. Mycophenolate mofetil (MMF) in patients with idiopathic membranous nephropathy with nephrotic syndrome: a multicenter randomized trial. *Journal of the american society of nephrology : JASN.* 2006;17(Abstracts):566A.

60. Li QH, Yang ZJ, Li L, et al. Comparison of efficacy and safety between tacrolimus and cyclosporine combined with corticosteroids in patients with idiopathic membranous nephropathy: A randomized controlled trial. *International Journal of Clinical and Experimental Medicine.* 2017;10(6):9764-9770.

61. Ponticelli C, Zucchelli P, Imbasciati E, et al. Controlled trial of monthly alternated courses of steroid and chlorambucil for idiopathic membranous nephropathy. *Proc Eur Dial Transplant Assoc.* 1983;19:717-723.

62. Ponticelli C, Passerini P, Salvadori M, et al. A randomized pilot trial comparing methylprednisolone plus a cytotoxic agent versus synthetic adrenocorticotropic hormone in idiopathic membranous nephropathy. *American Journal of Kidney Diseases.* 2006;47(2):233-240.

63. Li MX, Yu YW, Zhang ZY, Zhao HD, Xiao FL. Administration of low-dose cyclosporine alone for the treatment of elderly patients with membranous nephropathy. *Genet Mol Res.* 2015;14(1):2665-2673.

64. Jurubita R, Ismail G, Bobeica R, et al. Efficacy and safety of triple therapy with MMF, cyclosporine and prednisolone versus cyclosporine and prednisolone in adult patients with idiopathic membranous nephropathy and persistent heavy proteinuria. *Nephrology Dialysis Transplantation.* 2012;27(24).

65. Yao X, Chen H, Wang Q, et al. Cyclosporin A treatment for idiopathic membranous nephropathy. *Chin Med J.* 2001;114(12):1305-1308.

66. Saito T, Iwano M, Matsumoto K, et al. Mizoribine therapy combined with steroids and mizoribine blood concentration monitoring for idiopathic membranous nephropathy with steroid-resistant nephrotic syndrome. *Clin Exp Nephrol.* 2017;21(6):961-970.

67. Wang X, Song X, Liu Y, Zhang W, An W, Tu Y. Treatment of membranous nephropathy with mizoribine: A control trial. *Life Sciences.* 2016;154:75-78.

68. Nikolopoulou A, Condon M, Turner-Stokes T, et al. Mycophenolate mofetil and tacrolimus versus tacrolimus alone for the treatment of idiopathic membranous glomerulonephritis: a randomised controlled trial. *BMC Nephrol.* 2019;20(1):352.

69. Fernandez-Juarez G, Rojas-Rivera J, Logt AV, et al. The STARMEN trial indicates that alternating treatment with corticosteroids and cyclophosphamide is superior to sequential treatment with tacrolimus and rituximab in primary membranous nephropathy. *Kidney Int.* 2020.

70. Scolari FD, E.; Santor, D.; Gesualdo, L.; Pani, A.; Dallera, N.; Mani, L.; Santostefano, M.; Feriozzi, S.; Quaglia, M.; Boscutti, G.; Passerini, P.; Magistroni, R.; Alberici, F.; Ghiggeri, G.; Ponticelli, c.; Ravani, P. Rituximab versus cyclophosphamide in the treatment of membranous nephropathy. Paper presented at: ASN 20202020.
